# Mechanoactivated amorphization and photopolymerization of styryldipyryliums

**DOI:** 10.1038/s43246-024-00539-8

**Published:** 2024-06-08

**Authors:** Junichi Usuba, Zhenhuan Sun, Han P. Q. Nguyen, Cijil Raju, Klaus Schmidt-Rohr, Grace G. D. Han

**Affiliations:** Department of Chemistry, Brandeis University, 415 South Street, Waltham, MA 02453 USA

**Keywords:** Materials chemistry, Polymers

## Abstract

Conventional topochemical photopolymerization reactions occur exclusively in precisely-engineered photoactive crystalline states, which often produces high-insoluble polymers. To mitigate this, here, we report the mechanoactivation of photostable styryldipyrylium-based monomers, which results in their amorphization-enabled solid-state photopolymerization and produces soluble and processable amorphous polymers. A combination of solid-state nuclear magnetic resonance, X-ray diffraction, and absorption/fluorescence spectroscopy reveals the crucial role of a mechanically-disordered monomer phase in yielding polymers via photo-induced [2 + 2] cycloaddition reaction. Hence, mechanoactivation and amorphization can expand the scope of topochemical polymerization conditions to open up opportunities for generating polymers that are otherwise difficult to synthesize and analyze.

## Abstract (web summary)

Mechanical grinding of crystals aids in converting photostable polymorph to a photoactive one but is not widely applied to organic polymers. Here, mechanoactivation and amorphization of photostable styryldipyrylium ionic monomers are demonstrated.

## Introduction

Solvent- and catalyst-free topochemical polymerizations (TCP)^1–8^ of various solid-state monomers have emerged as a sustainable alternative to traditional solution-state polymerizations. The polymer products generally exhibit high crystallinity and large molecular weights^9–11^, resulting from the efficient single-crystal-to-single-crystal polymerization, and their depolymerization has also been demonstrated by the exposure to external stimuli^12–16^. TCPs have been generally achieved through judicious designs of monomer structures and precise engineering of their packing in crystals. For example, [2 + 2] photocycloadditions between monomers are viable only when the distance between reactive units is less than 4.2 Å, according to Schmidt’s principle^17^. To achieve a desirable arrangement of monomers in crystals, functional groups promoting intermolecular interactions such as hydrogen bonds, ionic interactions, and π-interactions have been integrated into the monomer structures^18–25^. Incorporating photochromes into frameworks has also enabled rigorous control over molecular arrangement and intermolecular interactions^26–32^. However, it is still not trivial to predict and control the photoreactivity of crystals solely based on the design of molecular structures, due to the occurrence of multiple viable polymorphs^33,34^.

**Fig. 1:**
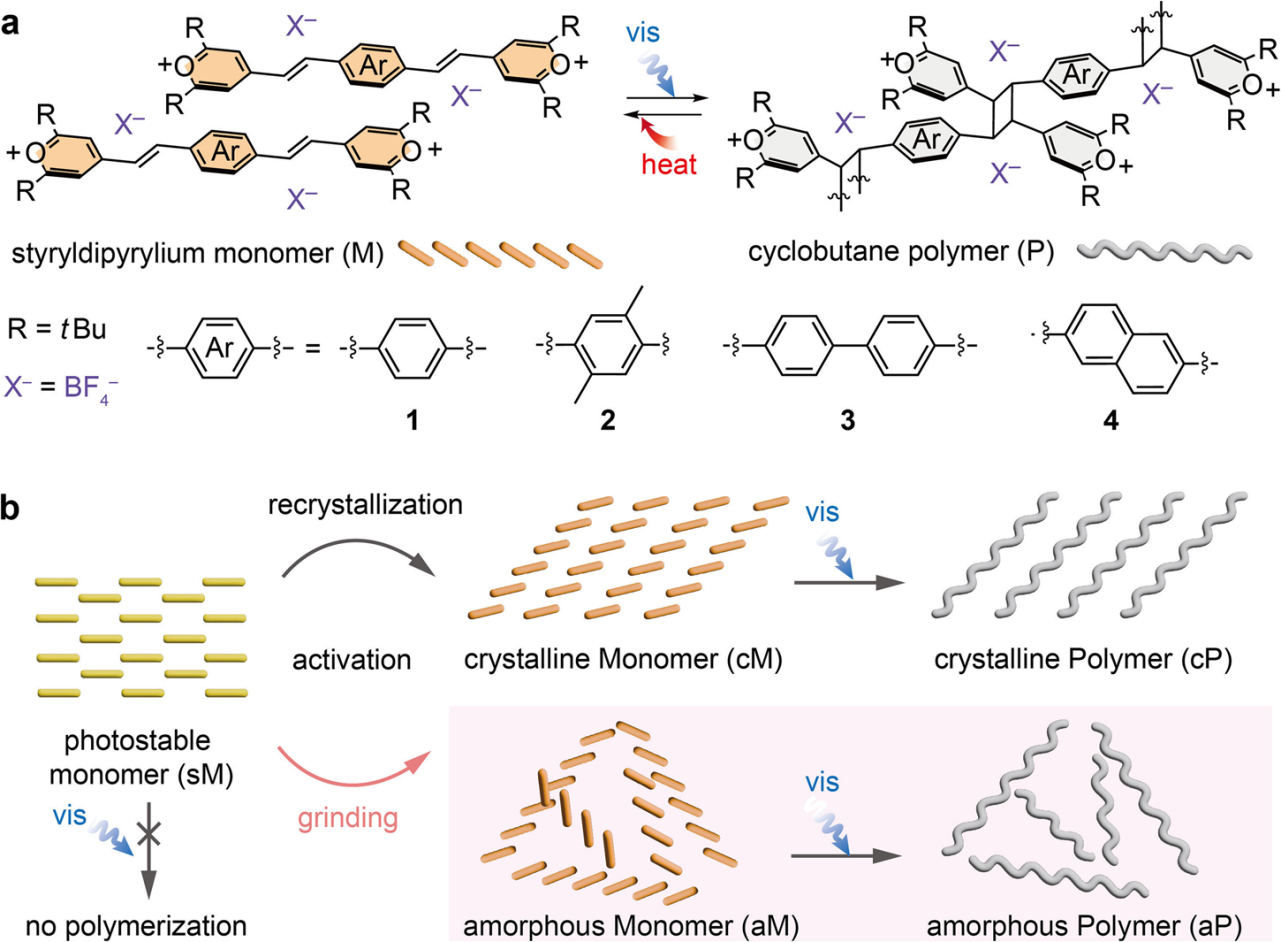
Solid-state polymerization of styryldipyryliums. **a** Chemical structure of the styryldipyrylium monomer and cyclobutane-linked polymer derivatives with varied arene spacers. **b** Schematic illustration of two activation methods of photostable monomer (sM).

One of the methods developed to convert a photostable polymorph to a photoactive one is the mechanical grinding of crystals. Mechanical stimuli can induce translational and/or rotational motions of molecules in the solid state that alter their orientations. Successful examples of such methods include [2 + 2] photocycloaddition reactions that produce metal coordination polymers^35–39^ or dimers within templated cocrystals^40–42^. Furthermore, simultaneous ball milling and irradiation have been studied in a newly developed series of apparatus^43,44^. However, the grinding method has not been widely applied to produce organic polymers. To the best of our knowledge, only (cyanostyryl)benzene^45^ has been mechanoactivated to produce short oligomers upon photoirradiation, which highlights that the applicability of such activation methods is system-dependent and requires further investigation. Herein, we report a successful mechanoactivation and amorphization of photostable ionic monomers, which expands the current scope of mechanoactivatable organic monomers (Fig. 1a). We selected styryldipyrylium derivatives as mechanoactivatable TCP monomer candidates bearing two reactive olefin units per molecule that are capable of [2 + 2] photocycloaddition if favorably arranged in crystals. Despite the electron acceptor-donor-acceptor structure of styryldipyryliums, their crystal packing is unpredictable, resulting in either photostable (X^−^ = BF_4_^−^, ClO_4_^−^, SnCl_6_^2−^) or photoactive (X^−^ = [SnCl_4_ (HCOO)]^−^, [SnCl_4_ (HCOO)(H_2_O)]^−^) crystals, largely depending on the type of counter-anions (X^−^)^46,47^. This molecular system clearly highlights the difficulty in achieving polymerizable monomer crystals, even after careful design and functionalization of monomer structures. To validate the substrate scope of the mechanoactivation method, we designed styryldipyrylium monomers **2–4** with varied solid-state packing and photophysical properties.

All monomers **1–4** are intrinsically photostable (sM), unfavorable for TCP. In principle, they could recrystallize into the photoactive crystalline form (cM), if suitable recrystallization conditions are found, to produce a crystalline polymer (cP) upon irradiation. However, the formation of cM by common recrystallization procedures has remained challenging for monomers **2–4**. Thus, we present an alternative mechanical alteration of monomer assemblies, which reliably produces photoactive amorphous monomers (aM) with short- or medium-range one-dimensional ordered structures that can be irradiated to form amorphous polymers (aP) (Fig. 1b). We hypothesize that pseudo-TCP occurs in a short- or medium-range-ordered amorphous solid^48,49^. This is distinguished from high pressure-induced TCP^50–60^, TCP via pressure-induced transition of solids from a photostable to a photopolymerizable crystalline phase^61,62^, or solid-phase polymerization that proceeds upon the injection of an external reagent^63^. The properties are compared between crystalline and amorphous polymers derived from the same monomer, which demonstrates the impact of activation methods on producing diverse polymers.

## Results and discussion

**Fig. 2:**
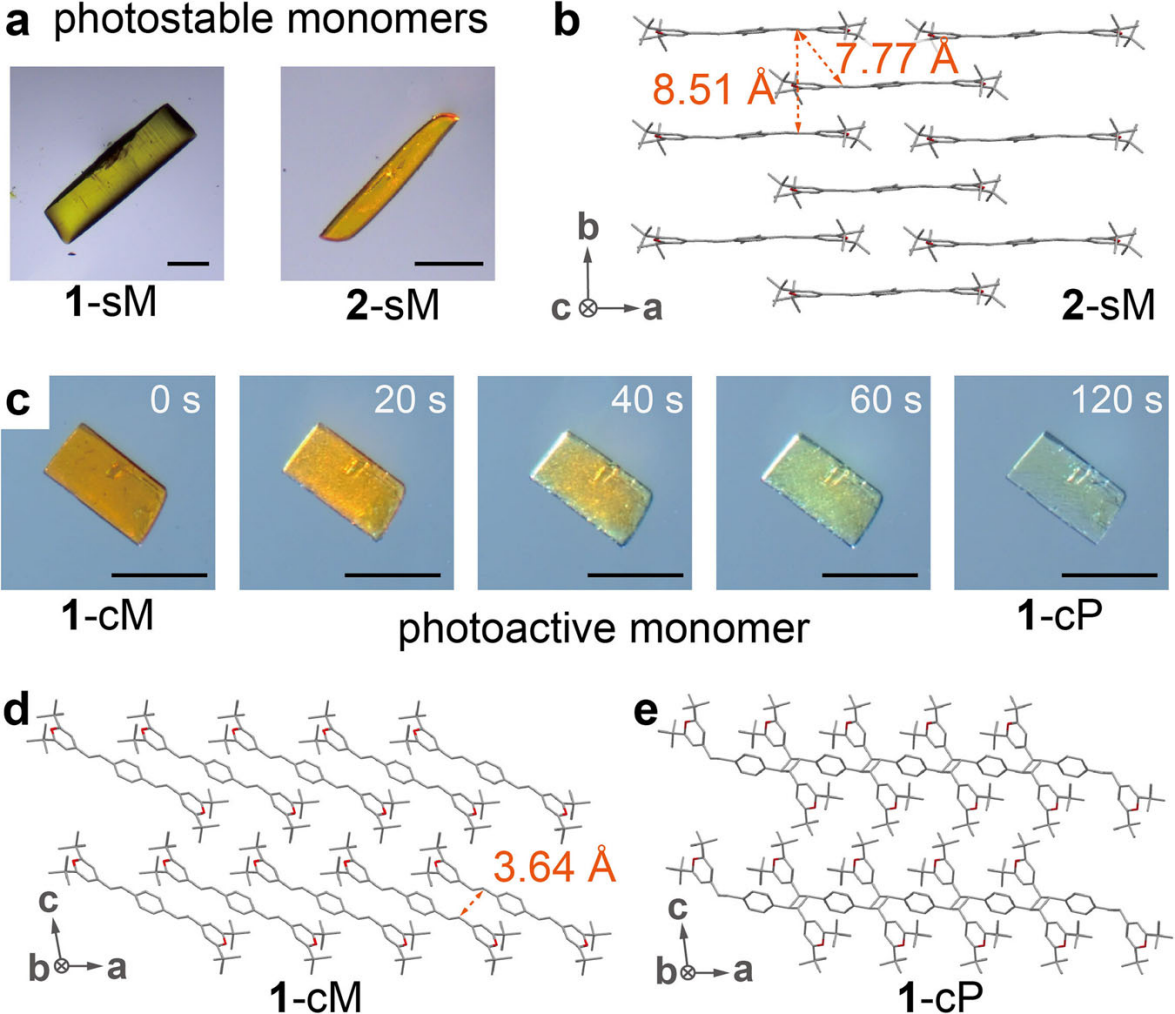
Photostable (sM) and photoactive (cM) polymorphs of monomer 1. **a** Optical microscope images of photostable crystals, **1**-sM and **2**-sM. Scale bar = 50 µm. **b** Crystal structure of **2**-sM. Hydrogen atoms and anions are omitted for clarity. **c** Optical microscope images showing a rapid conversion from **1**-cM to **1**-cP under irradiation by the tungsten–halogen lamp of the microscope. Scale bar = 50 µm. Crystal structures of **d** **1**-cM and **e** **1**-cP. Hydrogen atoms and anions are omitted for clarity.

Styryldipyrylium monomers (**1**–**4**) were synthesized by the trimolecular condensation reaction of pyrylium tetrafluoroborate salt with diformylarenes in ethanol (Supplementary Fig. S1). The products were precipitated as yellow-to-orange powders (labeled as **1**–**4** M initial) and washed with ethanol and diethyl ether for purification. The synthesis procedures were performed in ambient conditions on a gram scale (Supplementary Fig. S2). Single-crystal X-ray crystallography was performed to gain insight into the orientation of the monomers in the solid state. High-quality crystals of monomers **1** and **2** were grown by the slow diffusion of diethyl ether to acetonitrile solutions (Fig. 2a, Supplementary Figs. S3 and S4) and revealed to adopt a 1D columnar packing (Fig. 2b for compound **2**) where the distances between the neighboring olefins are 7.77 Å and 8.51 Å. The 1D columnar crystal structure of compound **1** is reported, showing a similar packing to **2**^46^. The large intermolecular distances exceeding 4.2 Å rationalize the photostability of the crystals^17^, i.e., no [2 + 2] photocycloaddition even after 24 h of irradiation at 470 nm, and such photostable monomers are labeled as **1**-sM and **2**-sM, respectively (Supplementary Fig. S5). A 470 nm LED (21.4 μW mm^–2^) was employed for irradiation, based on the maximum absorption wavelength of **1** at 485 nm in CH_2_Cl_2_ (vide infra). During the crystal growth, monomer **1** also afforded a minor polymorph (around 4%, Supplementary Figs. S6 and S7) that appears as orange crystals and displays rapid and complete photopolymerization under a broadband tungsten–halogen lamp (visible to infrared; used during the optical microscopy of crystals) or a 470 nm LED, accompanied by discoloration of the crystals (Fig. 2c, Supplementary Video 1). The crystal structure of this polymorph shows an offset π-stacked packing and inter-olefin distance of 3.64 Å (Fig. 2d, Supplementary Figs. S8–S10), which allows for facile [2 + 2] photocycloaddition (Fig. 2e). This photoactive crystalline form of compound **1** is labeled as **1**-cM to distinguish it from the photoactive amorphous form of compound **1** (**1**-aM, vide infra). The colorless polymer crystals (**1**-cP) produced by the irradiation were solved to be triclinic *P*-1, identical to the space group of **1**-cM, demonstrating single-crystal-to-single-crystal photochemical conversion with a small volume change of 2% (Supplementary Figs. S11 and S12, Table S1). Thus, **1**-cM is distinguished from a previously-reported photoactive styryldipyrylium (X^–^ = [SnCl_4_(HCOO)]^–^) that underwent an incomplete crystal-to-crystal conversion (80%) to yield short oligomers with less than 5 repeating units^46^.

**Fig. 3:**
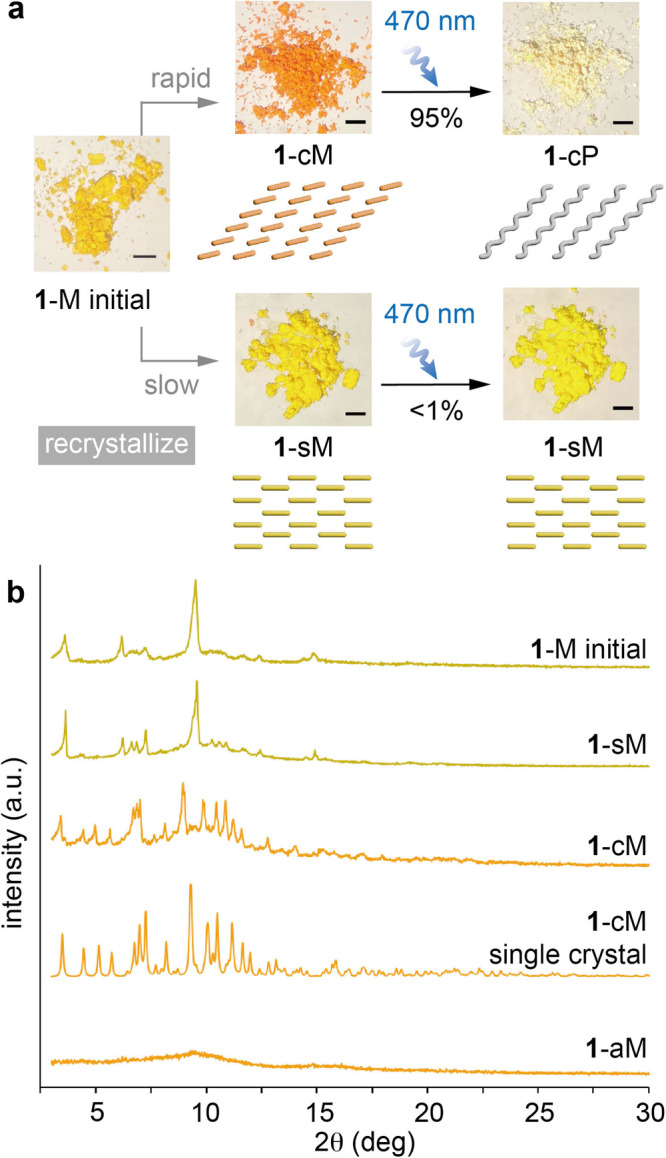
Recrystallization-induced polymorphism of compound 1. **a** The selective preparation of **1**-cM and **1**-sM by the recrystallization of monomer **1** and their response to irradiation (470 nm, 24 h). Scale bar = 1 mm. **b** PXRD patterns of the **1**-M initial, **1**-sM, **1**-cM, **1**-cM single crystal (simulated) and **1**-aM.

As mentioned before, most of the single crystals obtained from the crystallization of monomer **1** were photostable (**1**-sM), with a minor fraction of photoactive crystals (**1**-cM) (Supplementary Figs. S6 and S13). In order to maximize the yield of photoactive **1**-cM, various recrystallization methods were explored, and the rapid addition of diethyl ether to monomer **1** in acetonitrile was discovered to produce photoactive orange powder (Fig. 3a). The vigorous stirring was necessary to prevent the growth of thermodynamically-favored photostable crystals (**1**-sM) and to obtain primarily photoactive crystals (**1**-cM). The monomer conversion (%) was calculated as the ^1^H NMR integral ratio between the residual monomer and an internal standard (1,3,5-trimethoxybenzene). The orange powder undergoes a facile TCP (84% conversion of monomer in 3 h and 95% in 24 h) under 470 nm irradiation (Supplementary Figs. S14 and S15). The powder X-ray diffraction (PXRD) patterns of the orange powder before and after irradiation closely matched the patterns of **1**-cM and **1**-cP, respectively, which were simulated based on the crystallographic data of their single crystals (Fig. 3b, Supplementary Fig. S16). Thus, the orange powder obtained by the rapid recrystallization is considered to adopt the crystal packing of **1**-cM as displayed in Fig. 2d. On the other hand, the slow addition of diethyl ether to monomer **1** in acetonitrile over 30 min primarily afforded photostable yellow powder (Fig. 3a) that is expected to adopt molecular packing of **1**-sM. Thus, we were able to achieve a scalable and selective synthesis of **1**-cM and **1**-sM by controlling the kinetics of recrystallization^64^.

However, the rapid recrystallization method was not as effective in producing photoactive crystals (analogous to **1**-cM) for compounds **2**–**4**. Table 1 shows the low topochemical photo-conversion of recrystallized monomers **2** and **4** (<1% and 7%), measured by ^1^H NMR (Supplementary Figs. S17–S19). The monomer conversion only increased by 1–9% even after the rapid recrystallization, indicating that the majority of molecules adopt a photostable packing (Supplementary Fig. S20). We hypothesize that the structures of monomers **2** and **4** are intrinsically less likely to form a packing analogous to **1**-cM. Monomer **3** exhibits a more substantial increase in photoconversion by 23% upon rapid recrystallization; however, the maximum conversion remains 62%, highlighting the low efficacy of solution-based crystallization in achieving molecular arrangements that are susceptible to [2 + 2] photocycloaddition. The optical images and powder XRD patterns of compounds **1**–**4** prepared by the slow and rapid recrystallization as well as grinding are shown in Supplementary Figs. S21–24. We have also explored other solution-state recrystallization conditions, including the combinations of CHCl_3_, Et_2_O, ethanol, and hexanes, to find that the photostable packing is primarily obtained. Although further extensive screening of recrystallization conditions may be able to alter the crystal packing, herein, we highlight that the mechanoactivation of photostable monomers is an effective and alternative tool that yields higher photoconversion of monomers (Table 1).

**Table 1:** Photopolymerization by different monomer activation methods. Conversion (%) of monomers upon irradiation at 470 nm for 24 h in solid state, analyzed by ^1^H NMR

	Monomer activation		
Compound	Slow recrystallization	Slow recrystallization	Grinding
**1**	<1	95	100
**2**	<1	2	76
**3**	39	62	100
**4**	7	16	58

**Fig. 4:**
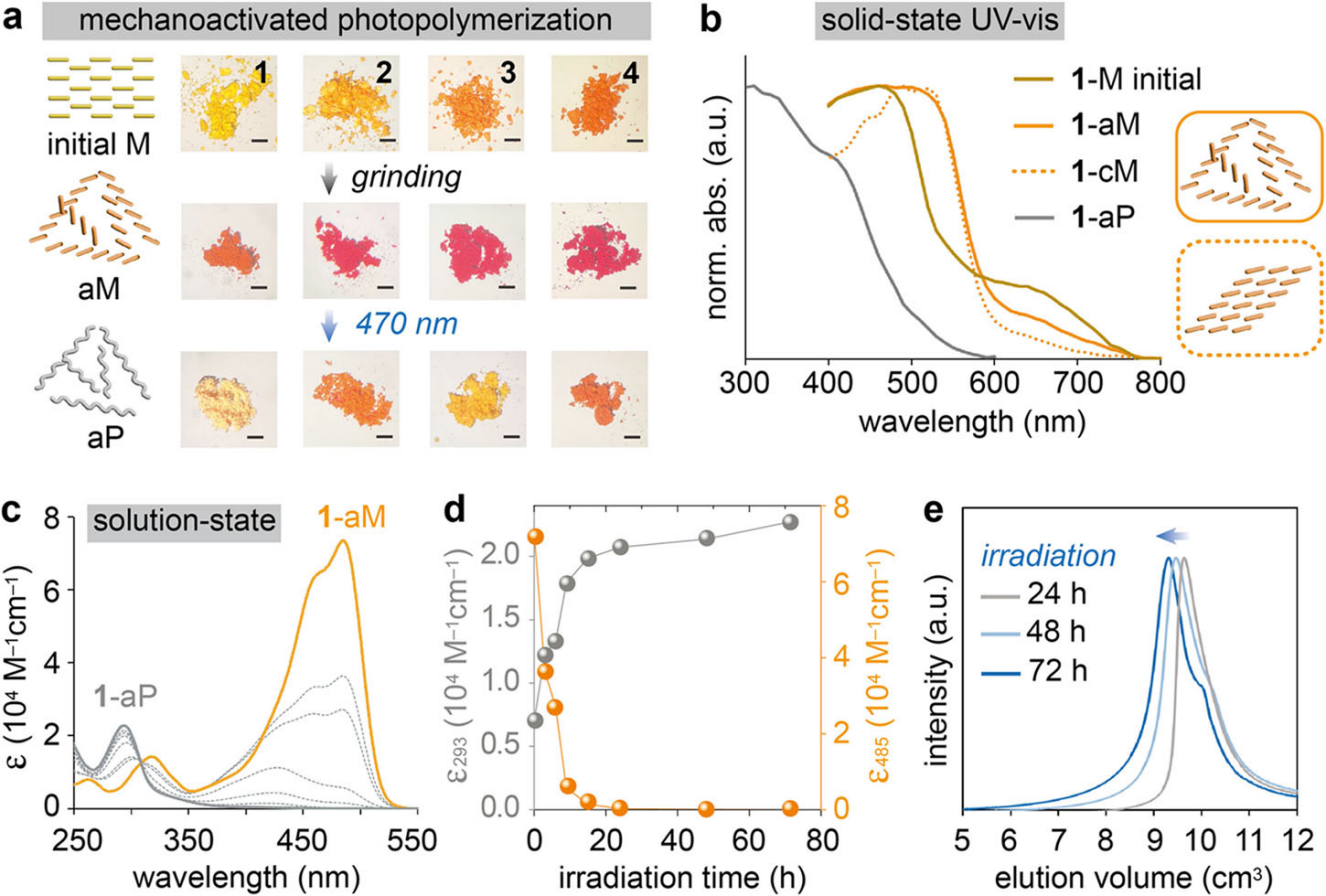
Amorphization-assisted photopolymerization. **a** Optical microscopy images of initial monomers (top), monomers ground in a mortar for 30 min (aM, middle), and those after irradiation at 470 nm for 24 h (aP, bottom). Scale bar = 1 mm. **b** Diffuse reflection spectra of **1**-M initial, **1**-aM (ground monomer), **1**-cM (recrystallized monomer), and **1**-aP (amorphous polymer). **c** UV–vis absorption spectra of CH_2_Cl_2_-dissolved aliquots of **1**-aM before (solid orange), during (dotted gray), and after 72 h irradiation (solid gray) at 470 nm. **d** Change of molar extinction coefficient of **1**-aM at 293 nm (gray) and 485 nm (orange) upon irradiation at 470 nm over time. **e** SEC plots of **1**-aP after increasing irradiation time as indicated, obtained by CH_2_Cl_2_ elution (1 cm^3^ min^−1^).

Monomer **1** changed from yellow crystals to orange amorphous powder, losing diffraction peaks, after 30 min of solvent-free grinding in an agate mortar (Fig. 3b, Fig. 4a, Supplementary Video 2). The amorphous powder (**1**-aM) is photoactive, turning off-white upon 470 nm light irradiation through photopolymerization and loss of conjugation. We monitored the PXRD pattern changes during the gradual dry grinding process (5, 15, and 30 min), which confirmed that mechanical stimulation results in amorphization, rather than a crystal-to-crystal phase transition (Supplementary Fig. S25. The amorphous phase **1**-aM is stable at room temperature, but heating it to 200 °C leads to its phase transition to **1**-sM (Supplementary Fig. S26). In addition, mechanoactivation by ball milling, using a vortex mixer^41,42^, was performed to reveal that the conversion of monomers by ball milling is generally lower than that of the mortar grinding method, without optimization (Supplementary Figs. S27–S29, Table S2). The solid-state diffuse reflectance spectra of **1**-aM and **1**-cM display similar profiles (Fig. 4b, Supplementary Fig. S30), both red-shifted from the spectrum of the initial photostable monomer **1**. The red shift indicates the formation of J-aggregates consisting of offset π-stacked monomers, which is supported by the TD-DFT calculated low-energy electronic transition of two neighboring monomers in **1**-cM crystal structures (Supplementary Fig. S31). In addition, the solid dispersion fluorescence spectrum of **1**-aM (*λ*_em_ = 554 nm) is also red-shifted from the spectrum of initial monomer **1** (*λ*_em_ = 517 nm), corroborating the formation of J-aggregates (Supplementary Fig. S32, vide infra). Compounds **2**–**4** also display the loss of diffraction peaks and red-shifted fluorescence upon grinding. Moreover, matching chemical shifts of **1**-aM and **1**-cM in solid-state ^13^C NMR suggests a similar local molecular environment in both photoactive solids (Supplementary Figs. S33a–c and S34). We note that mechanical grinding does not cause any decomposition of monomers, confirmed by ^1^H NMR analysis (Supplementary Fig. S35).

**1**-aM powder was sandwiched between two glass slides (around 119 µm thickness) and irradiated at 470 nm for photopolymerization (Supplementary Figs. S36). The polymerization of amorphous monomer was monitored by ^1^H NMR, UV–vis absorption spectroscopy, and size exclusion chromatography (SEC) of the produced polymer. ^1^H NMR confirms the complete loss of monomer peaks after 15 h of irradiation, which is accompanied by the appearance of broad polymer signals (Supplementary Fig. S37). We also performed the photopolymerization of **1**-M in CH_2_Cl_2_ solution (1 mM) to find a markedly lower conversion of 63% after 24 h of irradiation, compared to the solid-state polymerization (Supplementary Figs. S38 and S39). Similar ^1^H NMR spectral changes were observed during the photopolymerization of ground monomers **2–4** (Supplementary Figs. S40–S42), and time-dependent monomer conversion (%) under irradiation was monitored (Supplementary Fig. S43). Due to the broad signals, it was challenging to estimate the molecular weight of the polymer by end-group analysis. UV–vis absorption spectra (Fig. 4c, Supplementary Fig. S44), obtained by dissolving aliquots of the solid-state reaction mixture, also show the progress of the reaction. The prominent peak at 485 nm sharply decreased while the peak at 293 nm emerged over reaction time, which resembles the spectral change of styrylpyrylium during solid-state photo-dimerization, as recently reported^65^. The static penetration depth of **1**-aP at 470 nm is estimated to be 7.8 µm (Supplementary Fig. S45), while the sandwiched powder samples with an average thickness of 119 µm (Supplementary Fig. S36) underwent full conversion under the irradiation at 470 nm. This is attributed to the non-uniform and loose nature of powder samples, even in a sandwich, which allows for the deeper penetration of light.

Figure 4d shows the continual growth of the peak at 293 nm beyond 15 h irradiation, which marks the complete consumption of monomers, suggesting the further conversion of oligomers to longer polymers. SEC analysis confirmed the continuous growth of the polymer during the extended irradiation, and the weight average molecular weight (*M*_w_) of the produced **1**-aP after 72 h of irradiation was 1.8 × 10^4^ g mol^−1^ (Fig. 4e, Supplementary Table S3, Fig. S46). A lower *M*_w_ of 4.6 × 10^3^ g mol^−1^ was obtained when the monomer **1**-aM was irradiated at a longer wavelength (530 nm), and a higher *M*_w_ of 2.5 × 10^5^ g mol^−1^ when the same amount of monomer was spread thin over a larger irradiation area. These values are comparable to the *M*_w_ of other recyclable polymers^66^ and topochemical polymers^9–11,16^. Another monomer (**3**-aM) polymerized to similar *M*_w_ (6.7 × 10^4^ g mol^−^^1^), while **2**-aM and **4**-aM afforded oligomers (*M*_w_ < 2.0 × 10^3^ g mol^−^^1^). This observation indicates that monomers prone to form photoactive crystals (cM) upon recrystallization are also more likely to be mechanoactivated and photopolymerized. Therefore, we confirm that the success of solid-state photochemical reactions is dependent on both molecular designs and activation methods (Supplementary Fig. S47 and Table S4). We note that the molecular weights of ionic polymers are reported to be often underestimated because of the strong interactions between polymers and the stationary phase of the SEC, which also results in overestimated polydispersity indices (PDI)^11,67^. In our experiments, even the ionic monomers **1**–**4** exhibited broad SEC signals and large PDI values ranging from 1.9 to 3.2, because of such strong interactions (Supplementary Fig. S48). To further evaluate the molecular weights of polymers, we also performed diffusion-ordered NMR spectroscopy (DOSY)^68^, which revealed a wide range of diffusion coefficients of **1**-aP (5.9–2.0 × 10^−10^ m^2^ s^–1^) and a number average molecular weight (*M*_n_) of 7.0 × 10^3^ g mol^−^^1^ (Supplementary Fig. S49), corroborating the SEC analysis of polydisperse **1**-aP. We hypothesize that such polydispersity also results from the non-uniform grinding and irradiation, intrinsic to solid-state activation and reaction conditions.

**Fig. 5:**
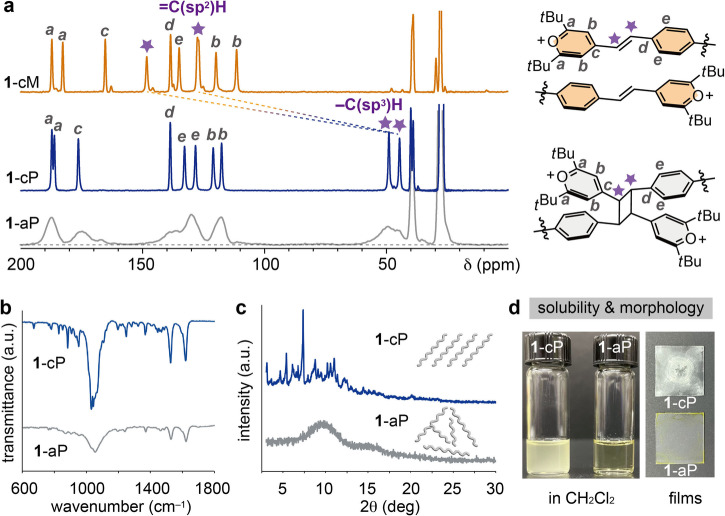
Comparison of crystalline and amorphous polymers prepared from identical monomers. **a** Solid-state ^13^C NMR of **1**-cM, **1**-cP and **1**-aP. To obtain the spectrum of pristine **1**-cP, any unreacted monomers were removed by rinsing (Supplementary section 7). **b** Infrared absorption spectra of **1**-cP and **1**-aP. **c** PXRD patterns of **1**-cP and **1**-aP. **d** Photographs of **1**-cP and **1**-aP in CH_2_Cl_2_ (1 g L^−1^) and their spin-coated films.

Solid-state ^13^C NMR was performed to clearly monitor the photo-induced conversion of olefin to cyclobutane. The matching chemical shifts of **1**-aP and **1**-cP confirm the formation of cyclobutane moieties upon the irradiation of both **1**-aM and **1**-cM (Fig. 5a, Supplementary Fig. S33d, e), and **1**-aP and **1**-cP also exhibit similar infrared (IR) spectra (Fig. 5b). The broader ^13^C NMR and IR peaks of **1**-aP, compared to those of **1**-cP, are attributed to the polydispersity and low crystallinity of **1**-aP, which is supported by the comparative PXRD patterns of **1**-aP and **1**-cP (Fig. 5c). From these spectroscopic results, we conclude that the chemical structures of **1**-cP and **1**-aP are analogous, despite the drastic difference in their crystallinity.

A large solubility difference between amorphous **1**-aP and crystalline **1**-cP was observed, highlighting the potential of mechanoactivated amorphous-state polymerization for creating soluble and processable polymers. **1**-aP shows high solubility in acetonitrile (>67 g L^−^^1^) and moderate solubility (2–6 g L^−1^) in other organic solvents, including dichloromethane, chloroform, and acetic acid (Supplementary Table S5), in sharp contrast to completely insoluble **1**-cP (Fig. 5d). Due to the insolubility of **1**-cP in all common organic solvents tested, solution NMR and SEC analysis of **1**-cP is not viable. In addition, MALDI-TOF mass analysis performed to compare the molecular weights of **1**-cP and **1**-aP did not yield any signals above the trimer, presumably due to fragmentation (Supplementary Fig. S50). The larger solubility of **1**-aP could be attributed to multiple potential factors, including the larger disorder in polymer backbones, lower inter-chain interactions, shorter polymer chains, and larger polydispersity, compared to **1**-cP. The solutions of **1**-aP could be spin-coated to form highly uniform and transparent films, whereas dispersions of **1**-cP formed inhomogeneous and opaque films (Fig. 5d, Supplementary Fig. S51).

We also investigated the thermally-induced depolymerization of **1**-cP and **1**-aP, building upon the successful cycloreversion of styrylpyrylium dimers in our previous report^65^. Differential scanning calorimetry (DSC) and thermogravimetric analysis (TGA) reveal the complete depolymerization of **1**-cP at 262 °C (Supplementary Figs. S52–55) and high thermal stability of the produced **1**-cM below 297 °C. The crystal structure of **1**-cP shows the cleavable C–C bond length of 1.589(3) Å in the cyclobutane units, which rationalizes the ease of cycloreversion^16^. In contrast, **1**-aP undergoes thermal decomposition at temperatures above 120 °C rather than depolymerization. The chemical stability of **1**-aP below 120 °C is confirmed by ^1^H NMR, solid-state ^13^C NMR, UV–vis absorption, SEC, and optical microscopy (Supplementary Figs. S56–S60). To explain the different thermal response of **1**-cP and **1**-aP, we hypothesize that mechanically disordered monomers (**1**-aM) can undergo [2 + 2] photocycloaddition to generate both all-*trans* (1*r*,2*r*,3*r*,4*r*) and *cis*-*trans* (1*R*,2*R*,3*S*,4*S*) cyclobutane isomers within the **1**-aP backbone, in contrast to **1**-cP solely composed of *cis*-*trans* isomer^69,70^. Our DFT calculation of model dimers suggests that an all-*trans* cyclobutane ring possesses a shorter C–C bond (1.571 Å) than that of a *cis*-*trans* cyclobutane ring (1.594 Å) (Supplementary Fig. S61, Tables S6 and S7). The presence of uncleavable all-*trans* cyclobutane rings in the **1**-aP backbone may be a reason for its different thermal properties from those of stereoregular **1**-cP. The marked differences in crystallinity, solubility, and thermal stability between **1**-aP and **1**-cP demonstrate how one can switch the activation method (mechanical vs. solution-based) to produce distinct polymers from the identical monomer.

**Fig. 6:**
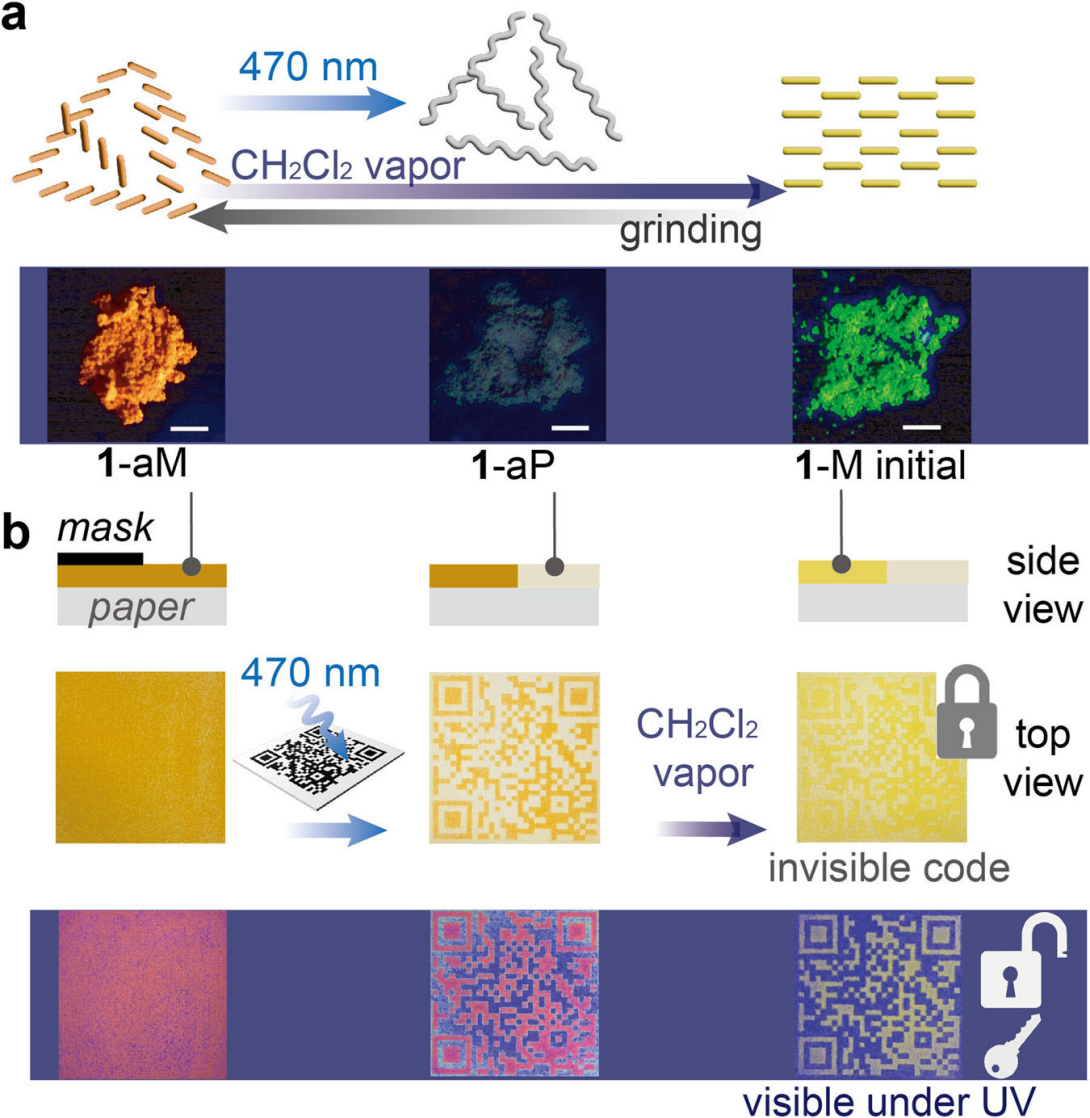
Pattern encryption enabled by multi-stimuli responsive materials. **a** Optical microscope images of **1**-aM, **1**-aP, and **1**-M initial under UV light in the dark. Scale bar = 1 mm. **b** Schematic illustration and optical images of the “locked” QR code generation via photopolymerization and solvent annealing of monomer **1**.

Lastly, the mechanoactivation of monomer and its solid-state photopolymerization enabled unique encryption application^71–74^. The solid-state emission of initial monomer **1** changes from green to orange by grinding, and the initial state is restored upon the exposure of **1**-aM to organic solvent vapor (CH_2_Cl_2_, ethanol, or acetic acid) (Fig. 6a and Supplementary Fig. S62). Also, 470 nm irradiation on **1**-aM generates **1**-aP that lacks fluorescence due to the loss of conjugation upon [2 + 2] cycloaddition. This force-, solvent-, and light-responsive fluorescence change of the compound was utilized to generate a security code that is unreadable under ambient light and only detected in the dark under UV illumination (Fig. 6b). A ground mixture of **1**-aM (5 wt%) and calcium sulfate was pressed into a chalk pellet and uniformly deposited on paper by drawing (Fig. 6b, Supplementary Fig. S63), which appeared pale orange under room light and showed red emission under UV. The color of the chromophore is diluted in the mixture with calcium sulfate. A QR code was printed on paper by 470 nm irradiation through an optical mask (Fig. 6b), discoloring the orange paper through photopolymerization and leaving the covered area intact. The initially printed QR code can be read both under ambient light and under UV (395 nm) in the dark, because of the colorless and non-fluorescent polymer **1**-aP. The QR code was then exposed to CH_2_Cl_2_ vapor for 5 min to make the code unreadable under the ambient light, turning the orange code to pale yellow that has insufficient contrast to the background (Fig. 6b). The background consisting of **1**-aP does not show any vapochromism. Thus, the information embedded in the QR code is “locked” under the ambient light and accessed only with a “key”: UV illumination in the dark. The green fluorescence of the QR code has sufficient contrast to the background, rendering it easily readable. Since the final QR code is encrypted by the combination of photostable monomer (**1**-M initial) and irreversible polymer (**1**-aP), the information is preserved even upon repeated exposure to UV and visible light. This security feature can be printed on various substrates, including fabric, frosted glass, plastics, and paper by abrasion, followed by irradiation through a mask and vapor annealing, which offers a simple and versatile encryption method.

## Conclusions

We discovered that all derivatives of photostable styryldipyrylium monomers **1**–**4** are more effectively activated by mechanical grinding than recrystallization, producing amorphous polymers or oligomers upon irradiation. Due to the large solubility, the amorphous products were easily characterized by solution-state NMR and SEC analyses, which were unviable for the corresponding crystalline polymers. In light of these findings, the mechanical activation of photostable crystalline monomers is revealed to be complementary to the common recrystallization method that may achieve the formation of a photoactive polymorph. Finally, the mechanically deposited monomers showcase drastic absorption and fluorescence changes upon irradiation or solvent annealing, which will offer a new avenue to encrypt information on a wide range of substrates. Although the mechanical-activation method has been tested only for styryldipyrylium-based monomers in the current study, the general applicability of this method will be elucidated through further investigations of a broader scope of monomers.

## Methods

### Materials and characterization methods

All reagents and starting materials were purchased from commercial vendors and used as supplied unless otherwise indicated. 2,6-di-*tert*-butyl-4-methylpyrylium tetraborate was prepared according to the literature^15^. Deuterated solvents were purchased from Cambridge Isotope Laboratories, Inc. and used as received. Solution state^1^H NMR and ^13^C{^1^H} NMR were recorded on a Varian INOVA 400 (400 MHz for ^1^H), 400MR (400 MHz for ^1^H and 101 MHz for ^13^C) and Bruker AVANCE NEO 800 (201 MHz for ^13^C) ^1^H NMR spectra are reported in *δ* ppm using the residual protons of the solvents, CH_2_Cl_2_ 5.32 ppm and CH_3_CN 1.94 ppm as an internal standard, those in ^13^C{^1^H} spectra are reported using the solvent signals of CD_2_Cl_2_ 53.84 ppm and CD_3_CN 1.32 ppm as an internal standard. ESI and MALDI-TOF mass spectra were obtained using Waters Synapt G2-Si ESI/LC-MS and Bruker Autoflex Speed LRF, respectively. LEDs were purchased from Thorlabs, Inc.: M470L5 (470 nm, 21.4 μW mm^−2^, 1161.7 mW); M530L4 (530 nm, 9.46 μW mm^−2^, 480 mW). Microscopic images and movies were taken using an OLYMPUS SZX-ILLD100 microscope equipped with an AmScope HD205-WU digital camera under a halogen tungsten lamp. Near-infrared absorption spectra were measured using a Nicolet™ Summit™ FTIR Spectrometer with attenuated total reflectance (ATR) method.

### Single-crystal X-ray diffraction (SC-XRD) measurements

Intensity data were collected from a Bulker single crystal X-ray diffractometer equipped with a Bruker X8 Dual imuS APEX2 detector with CuK*α* radiation (*λ* = 1.54184 Å) for **1**-cM, **1**-cP, and **2**-sM. The structures were solved SHELXT-2018/2 and refined by the full-matrix least-squares on SHELXL-2019/3^75,76^. All non-hydrogen atoms were refined anisotropically, and all hydrogen atoms were placed using AFIX instructions. Olex2 1.5^77^ was used as the GUI for these analyses. Data collection of **1**-cM was performed in dark conditions to prevent polymerization under ambient light.

### UV–vis absorbance spectroscopy

UV–vis absorption spectra of monomer (**1**–**4**) and their photo-irradiated samples in solution were obtained with a Cary 60 Bio UV–vis spectrophotometer in a UV Quartz cuvette with a path length of 10 mm. All compounds were dissolved in CH_2_Cl_2_ (for compound **1**) or acetonitrile (compound **2**–**4**) with concentrations ranging from 5 to 30 µM. The molar extinction coefficient was calculated on the basis of the concentration and molecular weight of the repeat unit *(*i.e., molecular weight of the monomer).

### Diffuse reflectance spectroscopy

Diffuse reflectance spectra were measured for 5–10 mg of powder sample in a sample holder equipped with a quartz disc of 15 mm diameter using a Perkin Elmer Lambda 1050 UV/vis/NIR spectrophotometer equipped with a 150 mm diameter integrating sphere. To confirm the impact of sample amount on measurement, the diffuse reflectance spectra of **1**-aM with varied amounts (5, 10, and 20 mg) were obtained (Supplementary Fig. S30). The absorbance (*A*_*S*_) of each powder sample were calculated by Eq. 1. from the reflectance %*R*_*S*_ of the sample and the reflectance %*R*_*B*_ of the background measurement.

1 $$As= - \log ( \% {R}_{B}/ \% {R}_{S})$$

### Fluorescence spectroscopy

Photoluminescence spectra were measured with a SHIMADZU RF-5301PC fluorescence spectrophotometer in a UV Quartz cuvette with a path length of 10 mm. All solid samples were measured as dispersed in diethyl ether. The solution sample was measured as a solution of low concentration CH_2_Cl_2_.

### Solid-state NMR

Solid-state NMR experiments were performed on a Bruker Avance Neo 400WB spectrometer at a ^13^C resonance frequency of 100 MHz and a ^1^H frequency of 400 MHz, using a Bruker 4-mm double-resonance magic-angle-spinning (MAS) probe head. About 60 mg of each sample was packed into a zirconia 4-mm MAS rotor with a 3-mm thick glass disk at the bottom and a KelF cap. The ^13^C NMR 90° pulse length was 4 μs, while the proton 90° pulse was ≤3.6 μs. In order to obtain nearly quantitative ^13^C NMR spectra of six materials derived from compound **1**-M with good signal-to-noise ratios, multiple cross-polarization (multiCP)^78^ was used at 14 kHz MAS with at least four repolarization delays of ~2 *T*_1H_ duration (ranging from 1 s to 1.75 s) separating cross polarization periods of 1.1 ms, after a recycle delay of at least 4 *T*_1H_ duration. Signal was detected, after a rotation-synchronized Hahn spin echo to avoid deadtime-induced baseline problems, with high-power ^1^H decoupling with |γ*B*_1_|/2*π* = 85 kHz. The same experiment was also performed with added recoupled dipolar dephasing by gating off ^1^H dipolar decoupling for two periods of 30 μs each, flanking the 180° pulse of the Hahn spin echo^79^.

Signal averaging for each pair of spectra was typically 20 h. The dipolar-dephased spectra consistently retained the peak at 140 ppm, showing that it must be assigned to substituted aromatic C, while the signal near 147 ppm, when present, was dephased and could therefore be assigned to =C–H; the high chemical shift indicated that this must be an olefin peak. ^13^C chemical shifts were indirectly referenced to TMS at 0 ppm using the COO resonance of 1-^13^C–glycine in the α-modification at 176.49 ppm as a secondary reference. In order to assess aromatic-ring packing and assign the olefin ^13^C NMR signals or the two cyclobutane ^13^C NMR peaks based on characteristic ^1^H chemical shifts, two-dimensional ^1^H–^13^C NMR heteronuclear correlation (HetCor) spectra with frequency-switched Lee–Goldberg homonuclear ^1^H decoupling were recorded at 14 kHz MAS with a cross-polarization period of 0.4 ms and a 90–100% amplitude ramp on ^1^H.

### Size exclusion column chromatography (SEC)

Analytical size exclusion column chromatography was performed on an Agilent 1100 Series HPLC system equipped with an Agilent diode array detector (G1313B). The polymer sample was eluted with CH_2_Cl_2_ at 1 ml min^−1^ 30 °C using an SEC column (Agilent PL-gel 5 µm MIXED-C 300 ×7.5 mm) calibrated with standard polystyrene reagents. The obtained signal was detected as absorbance at 300 nm, and the differential molecular weight curve and results are shown in Supplementary Figs. S46–S48 and Tables S3 and S4.

### Evaluation of number average molecular weight by DOSY NMR

The Stokes–Einstein equation (Eq. 2) describes the relationship between the radius (*r*) of a hydrodynamically equivalent sphere and its diffusion coefficient (*D*).

2 $$D={k}_{{{{{{\rm{B}}}}}}}T/6{{{{{\rm{\pi }}}}}}\eta r$$

where *k*_*B*_, *T*, and *η* are the Boltzmann coefficient, temperature, and viscosity, respectively.

The mass of a hydrodynamic sphere (*M*) can be calculated from the relationship between the volume of the sphere (*V*) and the radius (*r*).

3 $$V=3{{{{{\rm{\pi }}}}}}{r}^{3}/4=M/\rho$$

Here, *ρ* is the density. Thus, from (Eq. (2)) and (Eq. (3)), the mass (*M*), radius (*r*), and diffusion coefficient (*D*) have the following proportional relationship:

$$M\propto {r}^{3}\propto (1/{D}^{3})$$

Finally, according to the definition of degree of polymerization (DP) (Eq. (4)), the number average molecular weight (*M*_n_) was calculated from the diffusion coefficients of the monomer (*D*_0_) and polymer (*D*_poly_) determined by DOSY measurements, and the molecular weight of the monomer (*M*_0_).

4 $${{{{{\rm{DP}}}}}}={M}_{{{{{{\rm{n}}}}}}}/{M}_{0}={{D}_{0}}^{3}/{{D}_{{{{{{\rm{poly}}}}}}}}^{3}$$

The ^1^H DOSY NMR spectra of **1**-M and **1**-aP in CD_2_Cl_2_ at 20 °C are shown in Supplementary Fig. S49.

### Thin film preparation

Acetonitrile (0.25 mL) was added to 1 mg of **1**-aP or **1**-cP for spin-coating polymer films. A spin-coated film was prepared by dropping the entire volume of this solution (dispersion) onto a 1 × 1-inch glass slide at 100 rpm. The absorption spectra of these polymer films are shown in Supplementary Fig. S51.

### Differential scanning calorimetry (DSC)

DSC analysis was conducted on a DSC 250 (TA Instruments) with an RSC 90 cooling component. Heat flow was recorded 20 °C to 280 °C for **1**-aM, **1**-cM, and **1**-cP or 20 °C to 120 °C for **1**-aP respectively at 10 °C min^−1^ as 1^st^ heating. **1**-cP was recorded as a cooling cycle down to −100 °C to confirm depolymerization. The resulting DSC plots are shown in Supplementary Figs. S52 and S53.

### Computational methods

All of density functional theory (DFT) calculations were carried out using Becke’s three-parameter hybrid exchange functionals and the Lee–Yang–Paar correlation functional (B3LYP)^80^ and the 6–31 + G** basis set implemented in the Gaussian16 Revision B.01^81^ suite of programs with default thresholds and algorithms.

TD-DFT vertical excitation energy calculations were performed on the styryldipyrylium monomer and stacked dimer extracted from either the **1**-cM (J-aggregate) or **2**-sM (H-aggregate) crystal structure. The orbital energy diagram, selected Khon–Sham orbitals, and vertical energy oscillator strength are shown in Fig. S23.

Geometry optimization of the model compounds of a dimer of **1**-M (**1**_2_) was performed for two types of geometric isomers((1*R*,2*R*,3*S*,4*S*)*-***1**_2_ and (1*r*,2*r*,3*r*,4*r*)-**1**_2_). The stationary points were optimized without any symmetry assumptions and characterized by frequency analysis at the same level of theory (the number of imaginary frequencies, NIMAG, was 0). The cartesian coordinates for the optimized geometries are given in Supplementary Fig. S61 and Tables S6 and S7.

### Powder X-ray diffraction (PXRD) measurements

Powder X-ray diffraction (PXRD) patterns of compounds **1** and the rapidly recrystallized monomer **2** were obtained using a Malvern PANalytical Empyrean diffractometer equipped with a GaliPIX3D line detector with MoK*α* radiation (*λ* = 0.7093 Å, step size, 0.014°; time/step, 20 s). The PXRD patterns of other prepared monomers **2**–**4** were obtained using a Rigaku MiniFlex600 diffractometer with a CuK*α* source (*λ* = 1.5405 Å, step size, 0.010°; time/step, 0.6 s).

### Supplementary information

Supplementary Information

Description of Additional Supplementary Files

Supplementary Video 1

Supplementary Video 2

## Data Availability

The authors declare that the data supporting the findings of this study are available within the paper and its supplementary information files. The X-ray crystallographic coordinates for structures reported in this study have been deposited at the Cambridge Crystallographic Data Centre (CCDC), under deposition numbers 2309510, 2309511, and 2309512. These data can be obtained free of charge from The Cambridge Crystallographic Data Centre.
