# Supplementary material for: Mechanoactivated amorphization and photopolymerization of styryldipyryliums
Source: Commun Mater. 2024 Jun 8;5(1):98. doi: 10.1038/s43246-024-00539-8 (PMC11162349; doi:10.1038/s43246-024-00539-8)
Supplement: Supplementary file 1 — Supplementary Information [file 43246_2024_539_MOESM1_ESM.pdf]

Supplementary Information

Mechanoactivated Amorphization and Photopolymerization of Styryldipyryliums

Junichi Usuba[1], Zhenhuan Sun[1], Han P. Q. Nguyen[1], Cijil Raju[1], Klaus Schmidt-Rohr[1] & Grace G. D. Han[1]*

[1]Department of Chemistry, Brandeis University, 415 South Street, Waltham, MA, 02453, USA

E-mail: gracehan@brandeis.edu

Table of Contents

1. Synthesis and characterization of styryldipyrylium derivatives -----S2
2. Recrystallization of monomer -----S5
3. Activation of monomer -----S5
4. Solubility Measurement -----S5
5. QR code encryption -----S6
6. Crystal structures and microscope images of crystals -----S6
7. Photoinduced conversion in solid state -----S16
8. Mechanical grinding -----S23
9. TD-DFT calculation results -----S31
10. Fluorescence emission spectra -----S32
11. Solid-state NMR spectra -----S33
12. Photoinduced conversion in solution and in amorphous state -----S35
13. Static light penetration depth measurement -----S44
14. Molecular weights and solubilities of polymers -----S45
15. Thermal analyses -----S51
16. DFT calculation of isomers -----S57
17. Vapor annealing and preparation of solid pellets -----S62
18. ^{1}H and ^{13}C NMR spectra -----S63
19. Supplementary References -----S67

1. Synthesis and characterization of styryldipyrylium derivatives

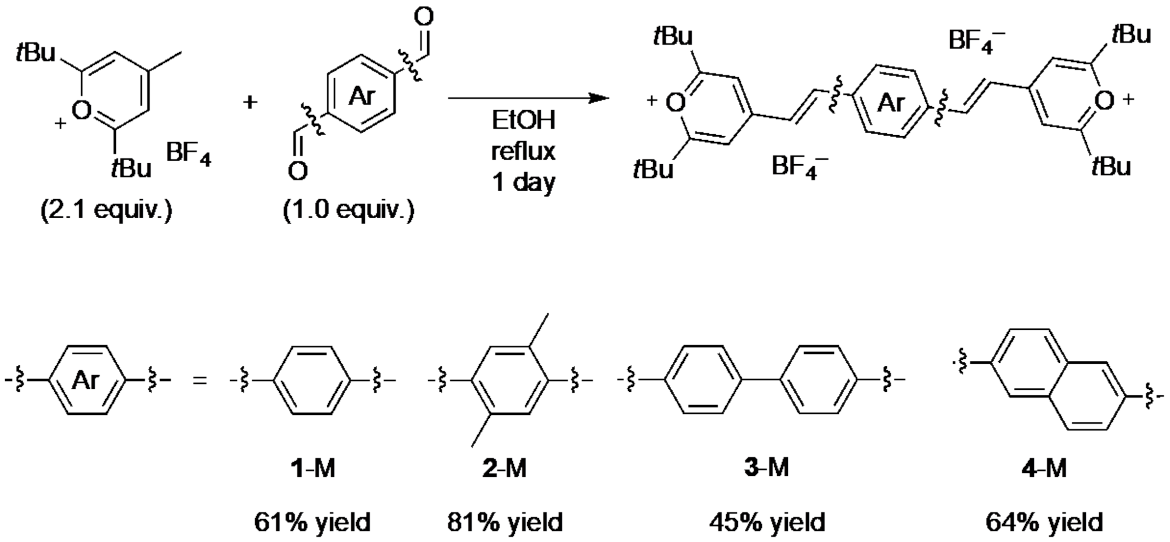

Figure S1. Synthesis of styryldipyrylium derivatives.

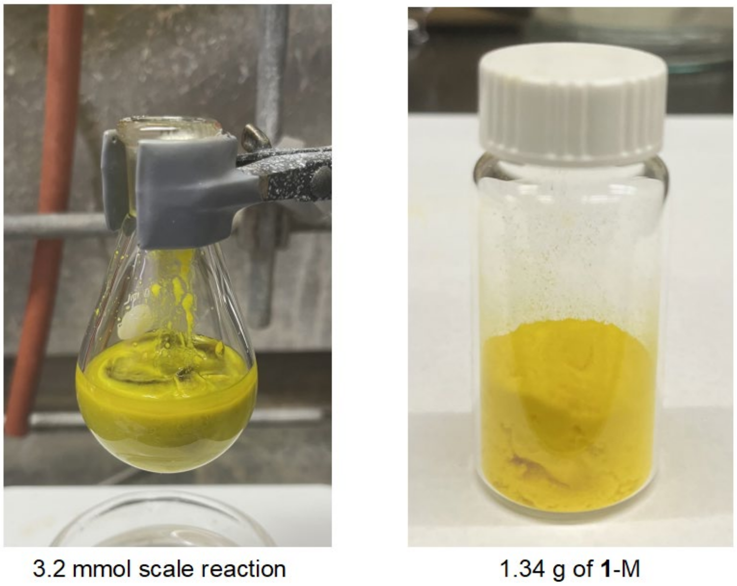

Figure S2. Gram scale synthesis of **1**-M.

a) 4,4'-((1*E*,1'*E*)-1,4-phenylenebis(ethene-2,1-diyl))bis(2,6-di-*tert*-butylpyrylium) tetraborate **(1-M)**

The round bottom flask was charged with terephthalaldehyde (433 mg 6.80 mmol), 2,6-di-*tert*-butyl-4-methylpyrylium tetraborate (2.00 g, 3.23 mmol), and 25 mL of ethanol. After reflux with stirring for 1 day in open air, the resulting precipitate was collected using a centrifuge, and the solid surface was washed with ethanol and diethyl ether. The residual volatiles were removed using a vacuum pump to afford 1.34 g of **1**-M initial (61% yield) as yellow powder. ^{1}H NMR (400 MHz, CD_2Cl_2): δ 8.31 (d, J = 16.1 Hz, 2H), 8.07 (s, 4H), 7.82 (s, 4H), 7.58 (d, J = 16.1 Hz, 2H), 1.56 (s, 36H). ^{13}C{^{1}H} NMR (201 MHz, CD_2Cl_2): δ 185.44, 165.65, 149.61, 138.33, 131.00, 125.46, 115.37, 39.28, 28.33. HRMS (ESI): *m*/*z* found $[M]^+$ for $C_{36}H_{48}O_2^{2+}$ 512.3648 (calcd. 512.3643) and $[M]^-$ for BF_4^- 87.0027 (calcd. 87.0029).

b) 4,4'-((1*E*,1'*E*)-(2,5-dimethyl-1,4-phenylene)bis(ethene-2,1-diyl))bis(2,6-di-*tert*-butylpyrylium) tetraborate (**2**-M)

This compound was prepared in a similar manner as described for **1**-M using 2,5-dimethylterephthalaldehyde (105 mg, 0.647 mmol) and 2,6-di-*tert*-butyl-4-methylpyrylium tetraborate (401 mg, 1.36 mmol) in ethanol (10 mL) to afford 375 mg of **2**-M initial (81% yield) as yellow powder. ^{1}H NMR (400 MHz, CD_3CN) δ 8.40 (d, J = 16.1 Hz, 2H), 7.96 (s, 4H), 7.84 (s, 2H), 7.49 (d, J = 16.1 Hz, 2H), 2.62 (s, 6H), 1.52 (s, 36H). ^{13}C{^{1}H} NMR (201 MHz, CD_3CN): δ 186.47, 165.27, 146.10, 139.14, 137.8, 131.04, 126.64, 116.08, 39.68, 28.36, 19.76. HRMS (ESI): *m*/*z* found $[M]^+$ for $C_{38}H_{52}O_2^{2+}$ 540.3962 (calcd. 540.3967) and $[M]^-$ for BF_4^- 87.0027 (calcd. 87.0029).

c) 4,4'-((1*E*,1'*E*)-[1,1'-biphenyl]-4,4'-diylbis(ethene-2,1-diyl))bis(2,6-di-*tert*-butylpyrylium) tetraborate (**3**-M)

This compound was prepared in a similar manner as described for **1**-M using 4,4'-biphenyldicarboxaldehyde (102 mg, 0.485 mmol) and 2,6-di-*tert*-butyl-4-methylpyrylium tetraborate (300 mg, 1.02 mmol) in ethanol (8 mL) to afford 169 mg of **3**-M initial (45% yield) as orange powder. ^{1}H NMR (400 MHz, CD_3CN) δ 8.36 (d, J = 16.3 Hz, 2H), 7.94 (s 4H), 7.93 (d, J = 7.3 Hz, 4H), 7.93 (d, J = 7.3 Hz, 4H), 7.50 (d, J = 16.3 Hz, 2H), 1.52 (s, 36H). ^{13}C{^{1}H} NMR (201 MHz, CD_3CN): δ 186.14, 165.68, 149.60, 144.17, 135.65, 131.34 129.07, 124.40, 115.56,

39.59, 28.35. HRMS (ESI): *m*/*z* found $[M]^+$ for $C_{42}H_{52}O_2^{2+}$ 588.3959 (calcd. 588.3967) and $[M]^-$ for BF_4^- 87.0031 (calcd. 87.0029).

d) 4,4'-((1*E*,1'*E*)-naphthalene-2,6-diylbis(ethene-2,1-diyl))bis(2,6-di-*tert*-butylpyrylium) tetraborate (**4**-M)

This compound was prepared in a similar manner as described for 1-M using naphthalene-2,6-dicarbaldehyde (60.0 mg, 0.326 mmol) and 2,6-di-*tert*-butyl-4-methylpyrylium tetraborate (200 mg, 0.680 mmol) in ethanol (6 mL) to afford 169 mg of **4**-M initial (45% yield) as orange powder. ^{1}H NMR (400 MHz, CD_3CN) δ 8.47 (d, *J* = 15.9 Hz, 2H), 8.34 (s, 2H), 8.15 (d, *J* = 8.7 Hz, 2H), 8.03 (d, *J* = 8.7 Hz, 2H), 7.97 (s, 4H), 7.60 (d, J = 15.9 Hz, 2H), 1.53 (s, 36H). ^{13}C{^{1}H} NMR (101 MHz, CD_3CN): δ 186.18, 165.37, 149.38, 135.87, 135.50, 131.44, 125.93, 125.33, 1s15.69, 39.55, 28.28. HRMS (ESI): *m*/*z* found $[M]^+$ for $C_{42}H_{52}O_2^{2+}$ 562.3792 (calcd. 562.3811) and $[M]^-$ for BF_4^- 87.0031 (calcd. 87.0029).

2. Recrystallization of monomers

For single-crystal XRD, **1**-sM, **1**-cM, and **2**-sM crystals were grown by the slow diffusion of diethyl ether vapor into acetonitrile solution of the monomer at room temperature. The ratio of **1**-sM to **1**-cM was estimated by counting the number of yellow crystals (**1**-sM) and orange crystals (**1**-cM) in microscopic images of the mixture obtained by recrystallization. Counting was performed on three microscope images, and an average value of 5% **1**-cM was obtained (Figure S6). In addition, the polymorphic mixture was irradiated at 470 nm for 24 h to determine the ratio (4% **1**-cM) by the solution state ^{1}H NMR (Figure S7).

3. Activation of monomers

a) Rapid recrystallization: 50 mg of monomer (**1**–**4**-M initial) was dissolved in 25 ml of acetonitrile in a brown flask. The monomer solution was stirred vigorously, and 125 ml of diethyl ether was rapidly added. After 5 min of stirring, the precipitate was collected by filtration.

b) Slow recrystallization: Diethyl ether was added to acetonitrile solution dropwise for 30 min (same concentration used in the rapid recrystallization method).

c) Grinding: **1**–**4**-aM were prepared by grinding the corresponding **1**–**4**-M initial in an agate mortar for 30 min.

Optical images and XRD patterns of rapid recrystallized, slow recrystallized, and ground monomers (**1**–**4**-aM) are shown in Figures S22–S25.

d) Ball milling: 30 mg of **1–4** M initial was added to a stainless-steel vial (9.5 mm inner diameter) with a silicone cap. The vial containing two stainless-steel balls (8 mm diameter) was fixed onto a vortex mixer and stirred for 30 min at 1800–2000 rpm. The equipment setup, PXRD pattern of ball-milled samples, ^{1}H NMR spectra after 24 hours of irradiation. and conversion (%) are shown in Figures S27, S28, S29, and Table S2.

4. Solubility Measurement

Dispersions of excess **1**-aP were filtered through PTFE, and 0.1 ml of filtrate was dried to measure the weight of the residue for the calculation of solubility. The solubilities of **1**-aP in dichloromethane, chloroform, acetonitrile, and acetic acid are shown in Table S5. The color of the THF, acetone, and DMF solution of **1**-aP slowly changed from pale yellow to brown or green, inferring decomposition. The isolation and characterization of the decomposition products have

been difficult. However, ring-opening reactions of the pyrylium ring triggered by a base or deprotonation of the α-position of the pyrylium ring could occur.[1]

5. QR code encryption

Pellets were prepared by pressing a mixture of **1**-aP (10 mg) and anhydrous calcium sulfate (200 mg) using an MSE PRO 10 mm diameter dry pellet pressing die set. **1**-aP was deposited on a filter paper by the abrasion of the pellet. An optical mask in the shape of a QR code was placed on the coated paper, which was then irradiated with a 470 nm LED for 3 hours. The QR code printed on the filter paper was exposed to CH_2Cl_2 vapor in a Petri dish at room temperature for 5 min. The QR code reading test was performed using an iPhone 13 mini (iOS 16.1.1).

6. Crystal structures and microscope images of crystals

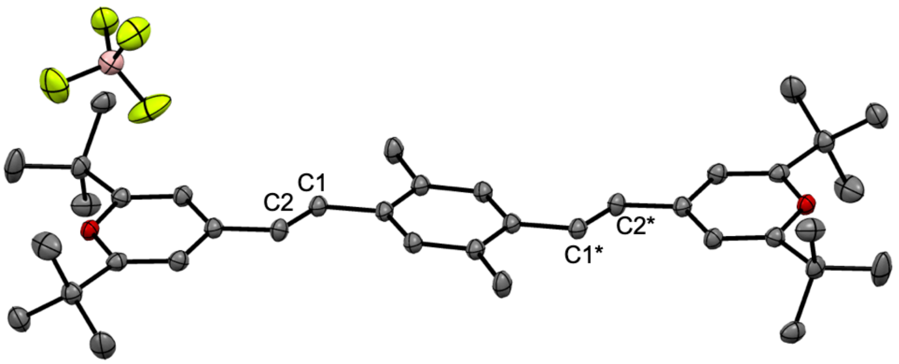

Figure S3. Crystal structure of **2**-sM (50% probability for thermal ellipsoids, hydrogen atoms are omitted for clarity).

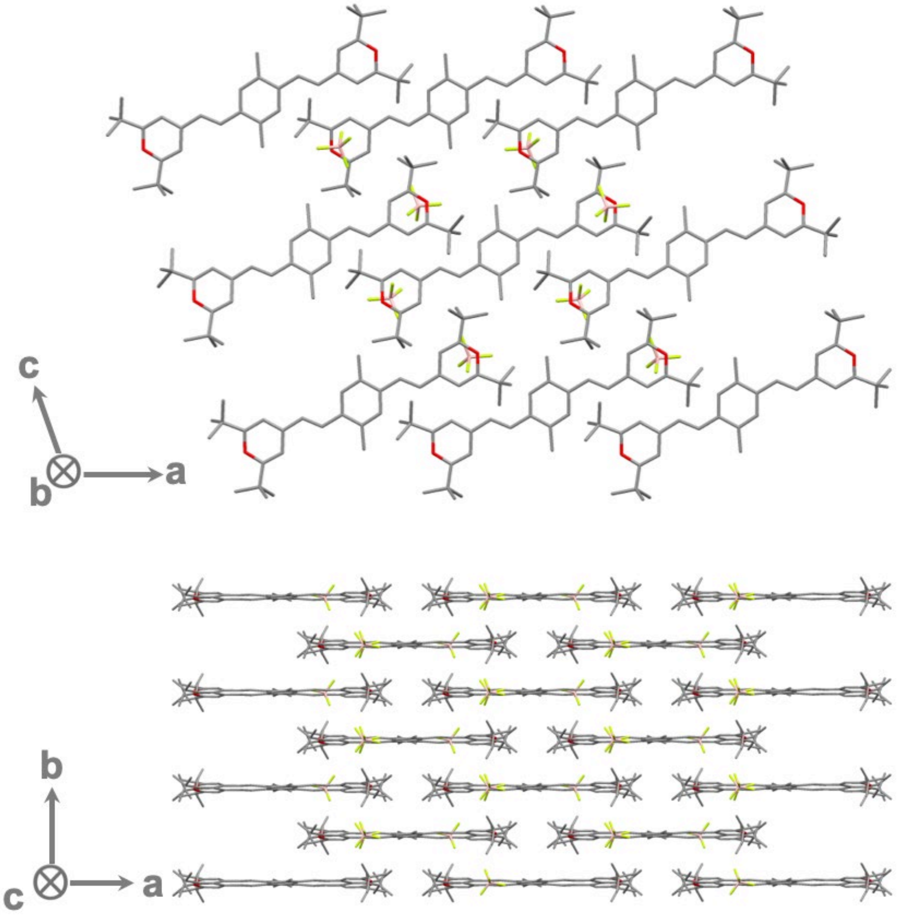

Figure S4. Packing structure of **2**-sM. Hydrogen atoms are omitted for clarity.

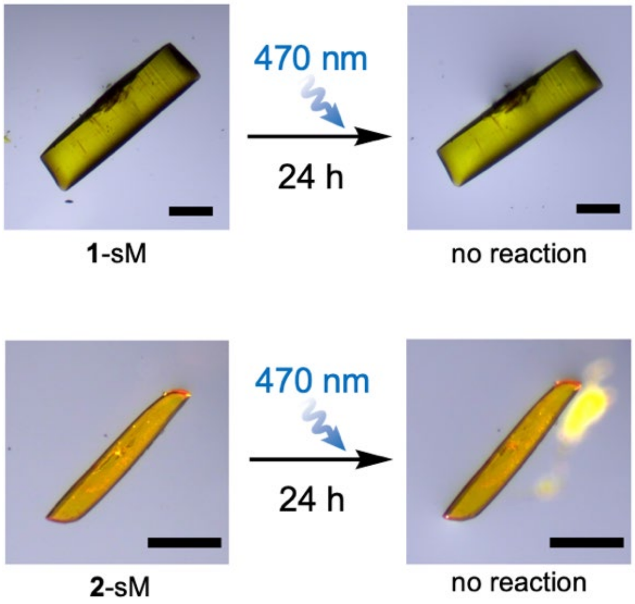

Figure S5. Optical microscope images of **1**-sM and **2**-sM single crystals, before light irradiation (left) and after 24 hours of 470 nm LED light irradiation (right) Scale bar = 50 µm

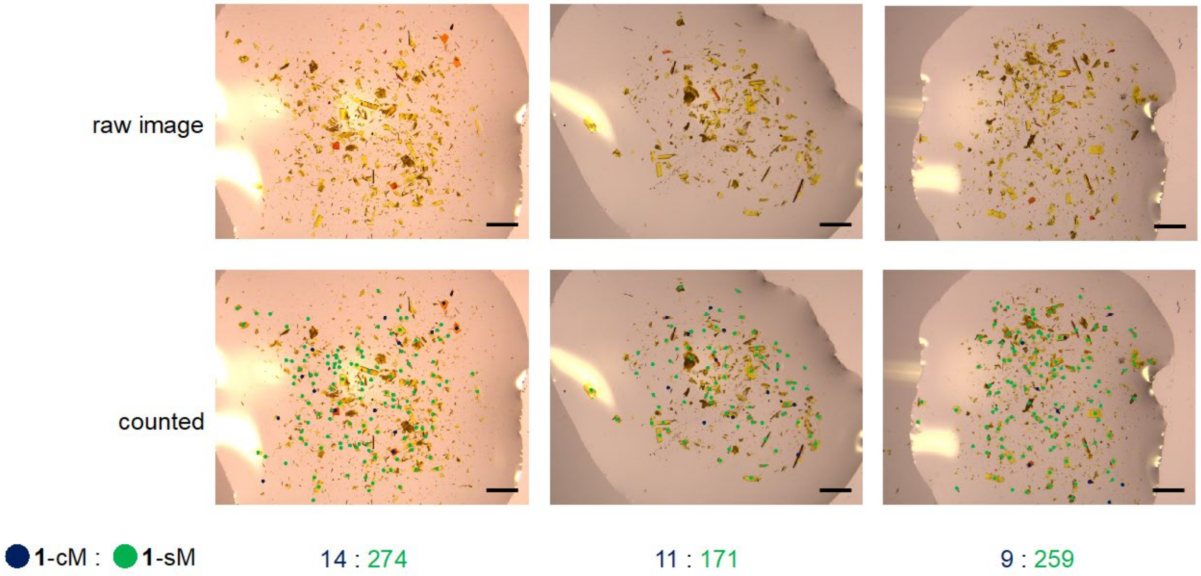

Figure S6. Optical microscope images of a **1**-sM and **1**-cM polymorph mixture obtained by recrystallized from MeCN/Et_2O. Raw images (top) and images marked for **1**-cM (blue) and **1**-cM (green) (bottom). Scale bar = 2 mm

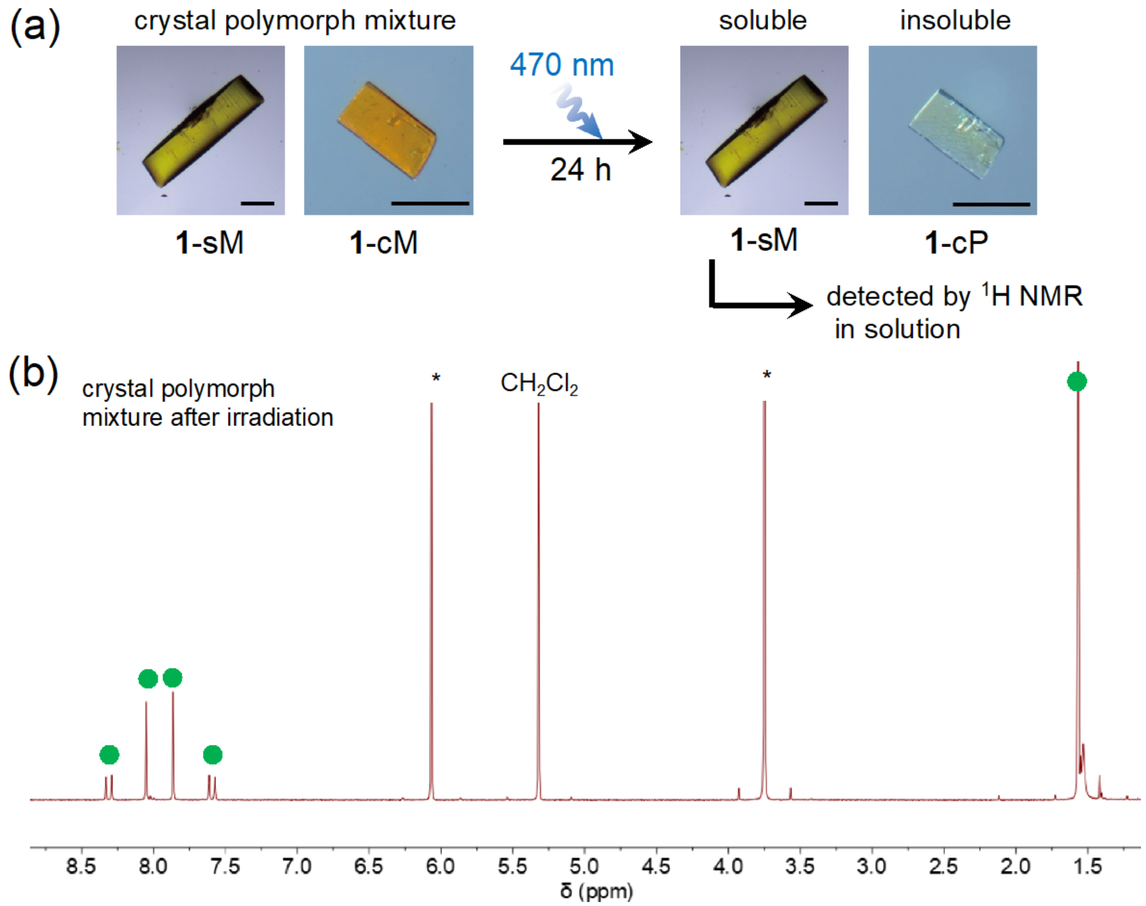

Figure S7. Determination of the ratio of crystal polymorph mixtures. (a) Optical microscope images of **1**-sM, **1**-cM and **1**-cP. Scale bar = 50 μm (b) ^{1}H NMR spectrum of a crystal polymorph mixture irradiated at 470 nm for 24 h, measured in CD_2Cl_2. Green circles denote the signals of unreacted **1**-sM, and the asterisks denote the signals of 1,3,5-trimethoxybenzene as an internal standard. The calculation method of content of **1**-sM (%) can be found in Section 7.

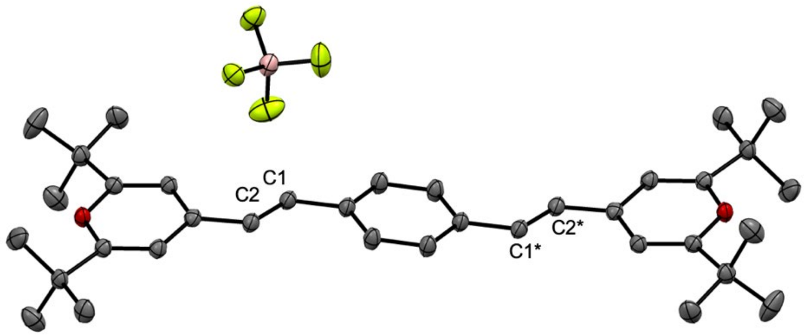

Figure S8. Crystal structure of **1**-cM (50% probability for thermal ellipsoids, hydrogen atoms are omitted for clarity).

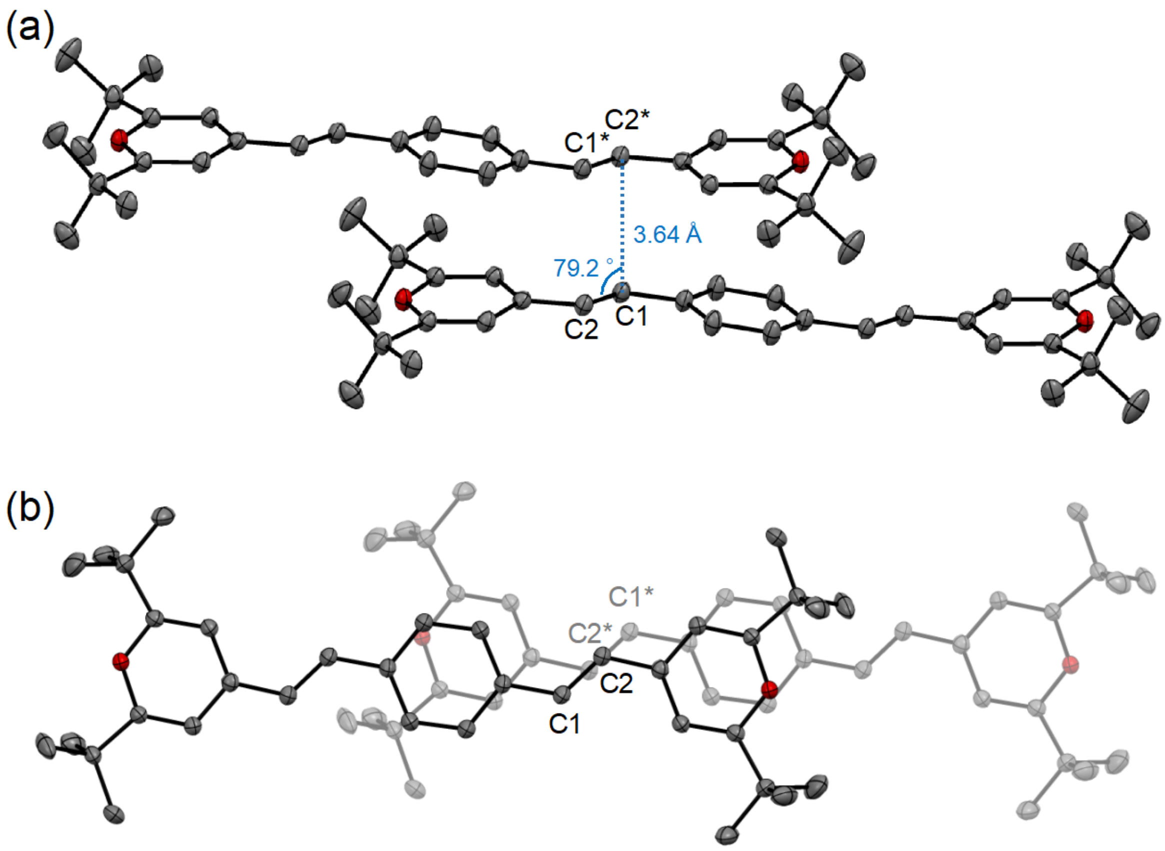

Figure S9. Thermal ellipsoid plots of two crystallographically equivalent adjacent molecules of **1**-cM: (a) side view and (b) top view. Thermal ellipsoids set at 50 % probability. Hydrogen atoms and counter-anions were omitted for clarity.

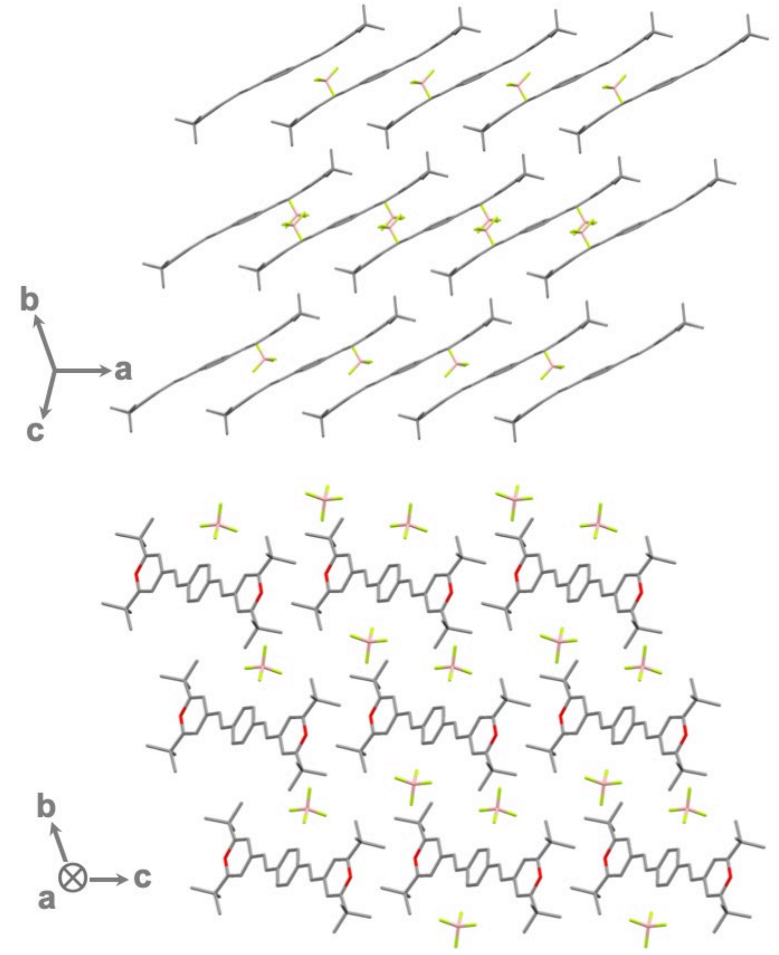

Figure S10. Packing structure of **1**-cM. Hydrogen atoms are omitted for clarity.

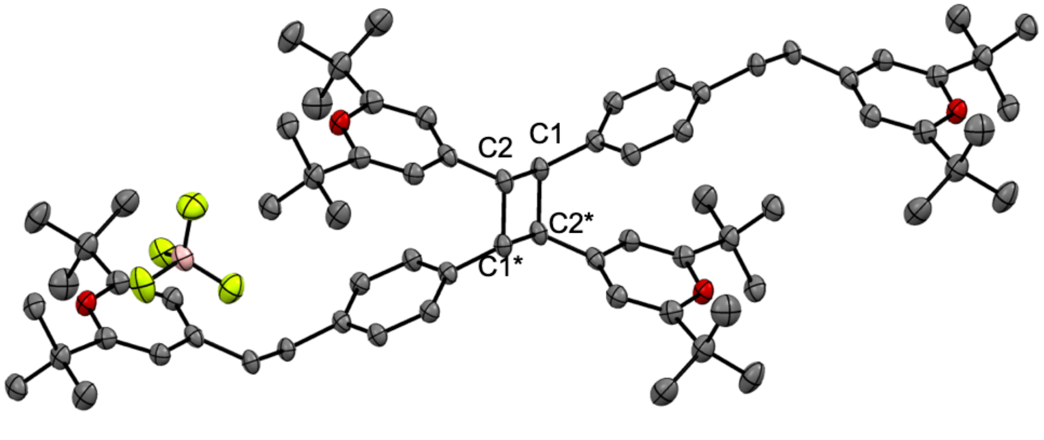

Figure S11. Crystal structure of **1**-cP (50% probability for thermal ellipsoids, hydrogen atoms are omitted for clarity).

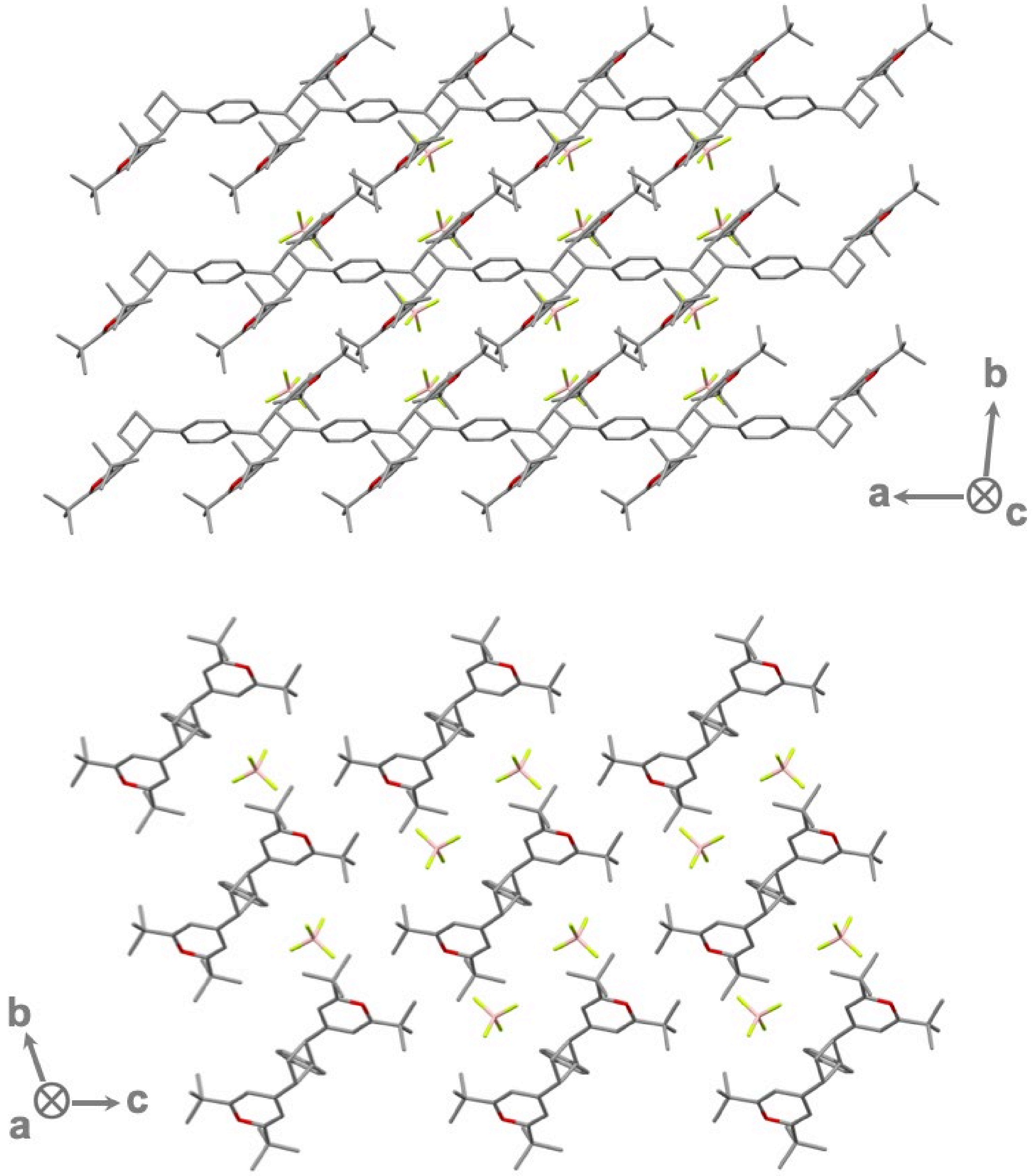

Figure S12. Packing structure of **1**-cP. Hydrogen atoms are omitted for clarity.

Table S1. Crystallographic data of **2**-sM**,** **1**-cM and **1**-cP.

Compound	**2**-sM	**1**-cM	**1**-cP
Formula weight	714.42	686.36	686.36
Crystal system	monoclinic	triclinic	triclinic
Space group	*C*12/*c*1	*P*-1	*P*-1
Crystal color	yellow	orange	colorless
Crystal description	needle	plate	plate
Crystal size / mm^3	0.3×0.07×0.06	0.2×0.12×0.025	0.18×0.12×0.12
a / Å	23.5518(15)	8.0260(4)	7.5269(10)
b / Å	8.5047(5)	9.5778(4)	9.1427(12)
c / Å	19.9873(11)	12.3550(6)	13.4892(19)
α / deg	90	107.147(2)	108.408(8)
β / deg	108.884(3)	98.902(2)	92.085(9)
γ / deg	90	90.081(2)	94.308(8)
V / Å^3	3788.0(4)	895.49(7)	876.5(2)
Z	1	1	1
d / g cm^{-3}	1.253	1.273	1.300
R_1 ($I > 2\sigma(I)$)	0.0430	0.0482	0.0729
wR_2 (all data)	0.1207	0.1451	0.1858
Goodness-of-fit	1.057	1.021	1.149
Temperature / K	100	100	100
CCDC No.	2309510	2309512	2309511

Single-crystal X-ray diffraction (SC-XRD) structure of 1-sM

It is reported that the intermolecular olefin carbon distance of **1**-sM is 9.61 Å.[2] However, because the cif. file was not available, we grew **1**-sM crystals for single-crystal. However, the quality of solved data was insufficient, despite the analytical efforts including the analysis of nonmerohedral twinning, application of pseudo-merohedral twin laws (three different monoclinic settings with their respective twin transformations to orthorhombic *P* and I), and modeling whole-molecule disorder. Additionally, the data were not resolved based on the reported cell parameters. ($P2_1/n$, a = 9.6146(5) Å, b = 14.980(1) Å. c = 19.714(2) Å, β = 92.527(5)°)

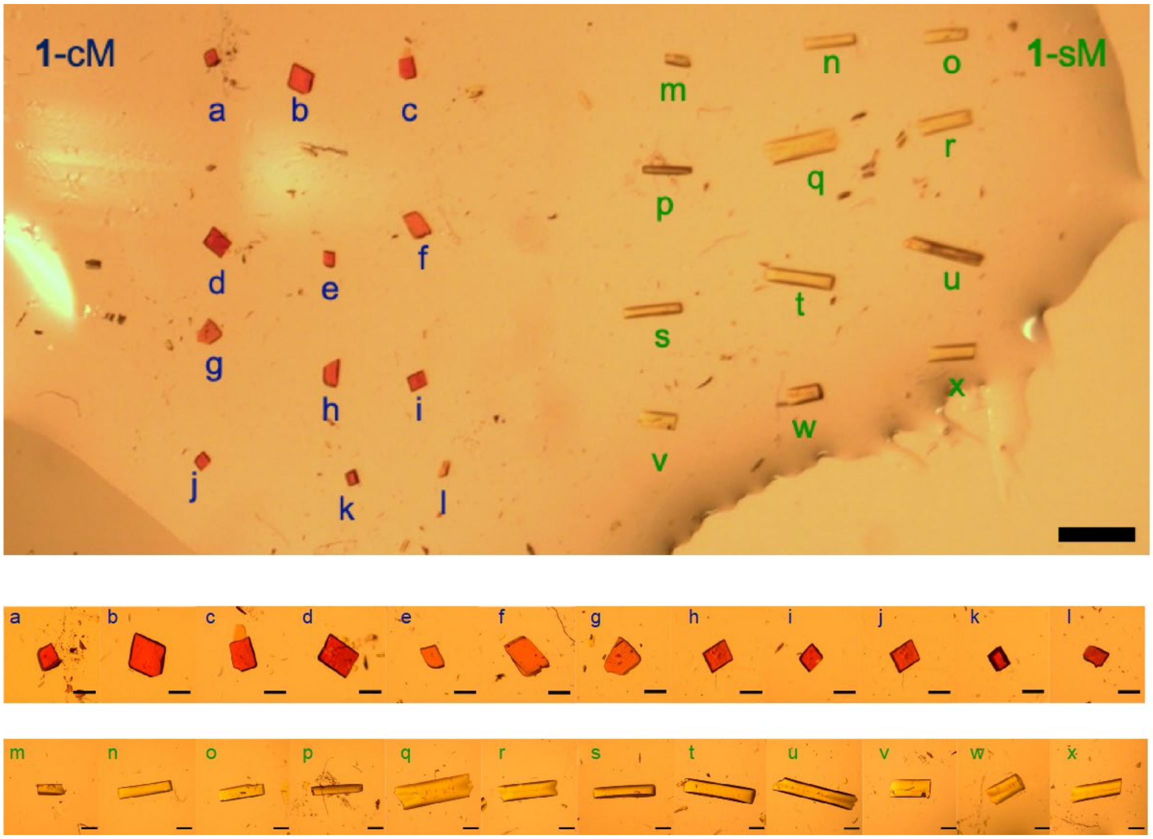

(a)
1-cM
1-sM
a b c d e f g h i j k l
m n o p q r s t u v w x
(b)
a b c d e f g h i j k l
m n o p q r s t u v w x

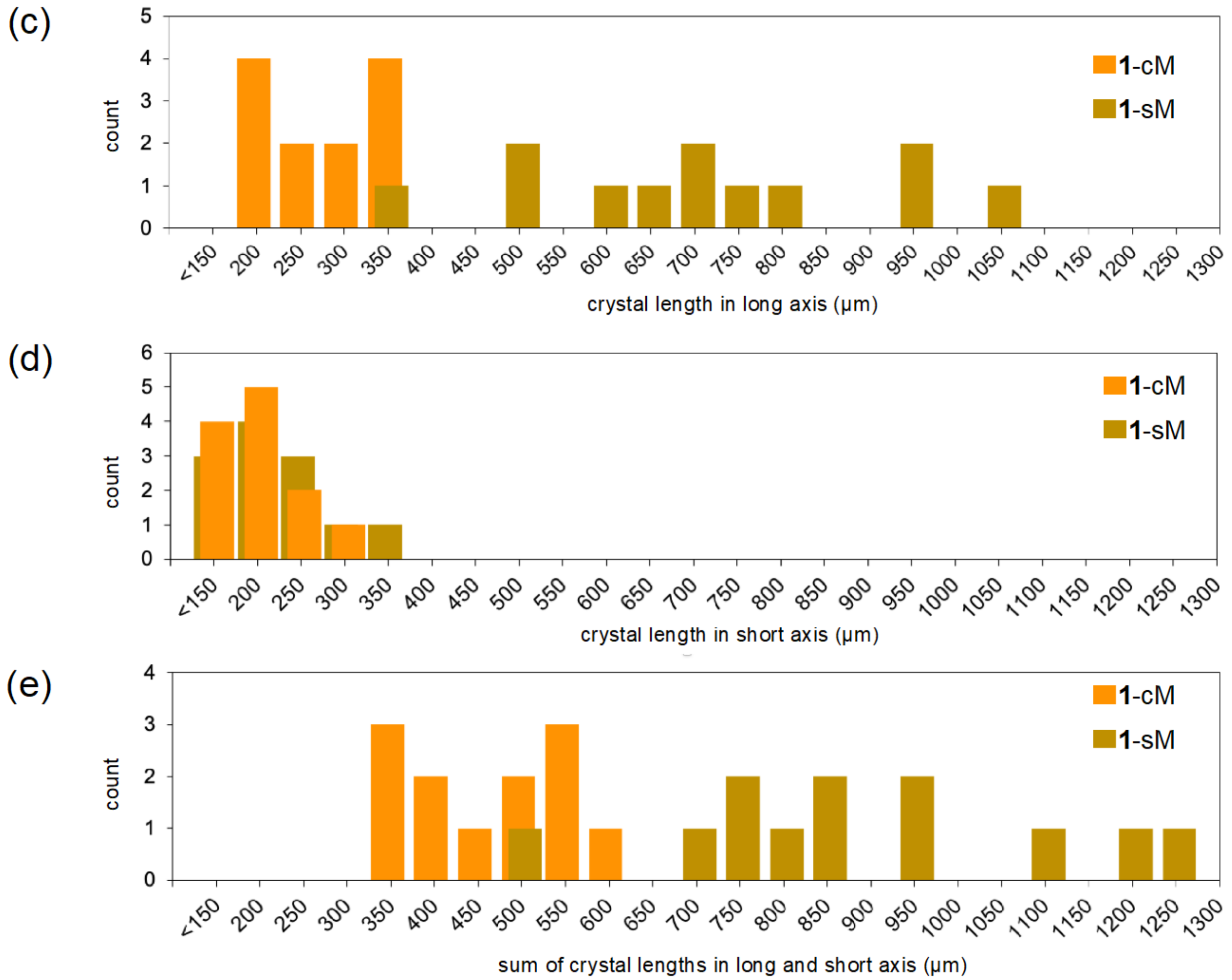

Figure S13. Measurement of the size of slowly-grown crystals of compound **1**. (a) An optical microscope image of crystals. Scale bar = 1 mm (b) Enlarged image of each crystal. Scale bar = 200 μm. Histogram of the lengths of **1**-cM (orange) and **1**-sM (yellow) crystals along their (c) long and (d) short axis, and (e) the sum of both lengths.

7. Photoinduced conversion in solid state

Unless otherwise noted, photoinduced polymerizations were performed using 470 nm LED, vertically placed above a 10 mg monomer sample sandwiched between 3”x1” glass slides (or 5 mg between 1.5”x1” slides). The thickness of sandwich films was measured using Zeta-20 Optical Profilometer. After irradiating **1**-cM for 24 h, the remaining unreacted monomer was removed by acetonitrile decantation to obtain **1**-cP.

Monomer conversion (%) was calculated from the ^{1}H NMR integral ratio of the residual monomer signal and the signal of 1,3,5-trimethoxybenzene added as an internal standard reagent. The integral value of the peak assigned to the arene hydrogen 3H of 1,3,5-trimethoxybenzene was normalized to 1. The integral value of the peak assigned to 4H on the pyrylium ring of the residual monomer is defined as S_{exp}.

The residual monomer (%) is

$$\text{residual monomer (\%)} = S_{\mathrm{exp}} / S_{100} \times 100 \qquad \text{-----eq.1}$$

where S_{100} is defined as the integral value that could be obtained if 100% of the monomer is present in the sample (i.e., reaction mixture).

Thus, it is calculated as

$$S_{100} = 4(w_{\mathrm{s}} / M_{\mathrm{m}}) / 3(w_{\mathrm{ref}} / M_{\mathrm{ref}}) \qquad \text{-----eq.2}$$

where w_{s}, M_{m}, w_{ref}, and M_{ref} are the sample weight, the molecular weight of the monomer, the weight of the internal standard reagent, and the molecular weight of the internal standard reagent, respectively.

Finally, the monomer conversion (%) is calculated as

$$\text{monomer conversion (\%)} = 100 - \text{residual monomer (\%)} \qquad \text{-----eq.3}$$

Since this calculation method utilizes both the relative mass of sample and standard (w_{s} and w_{ref}) as well as the integral value from NMR (S_{exp}), the direct comparison of any peaks within an NMR spectrum does not translate to the conversion (%).

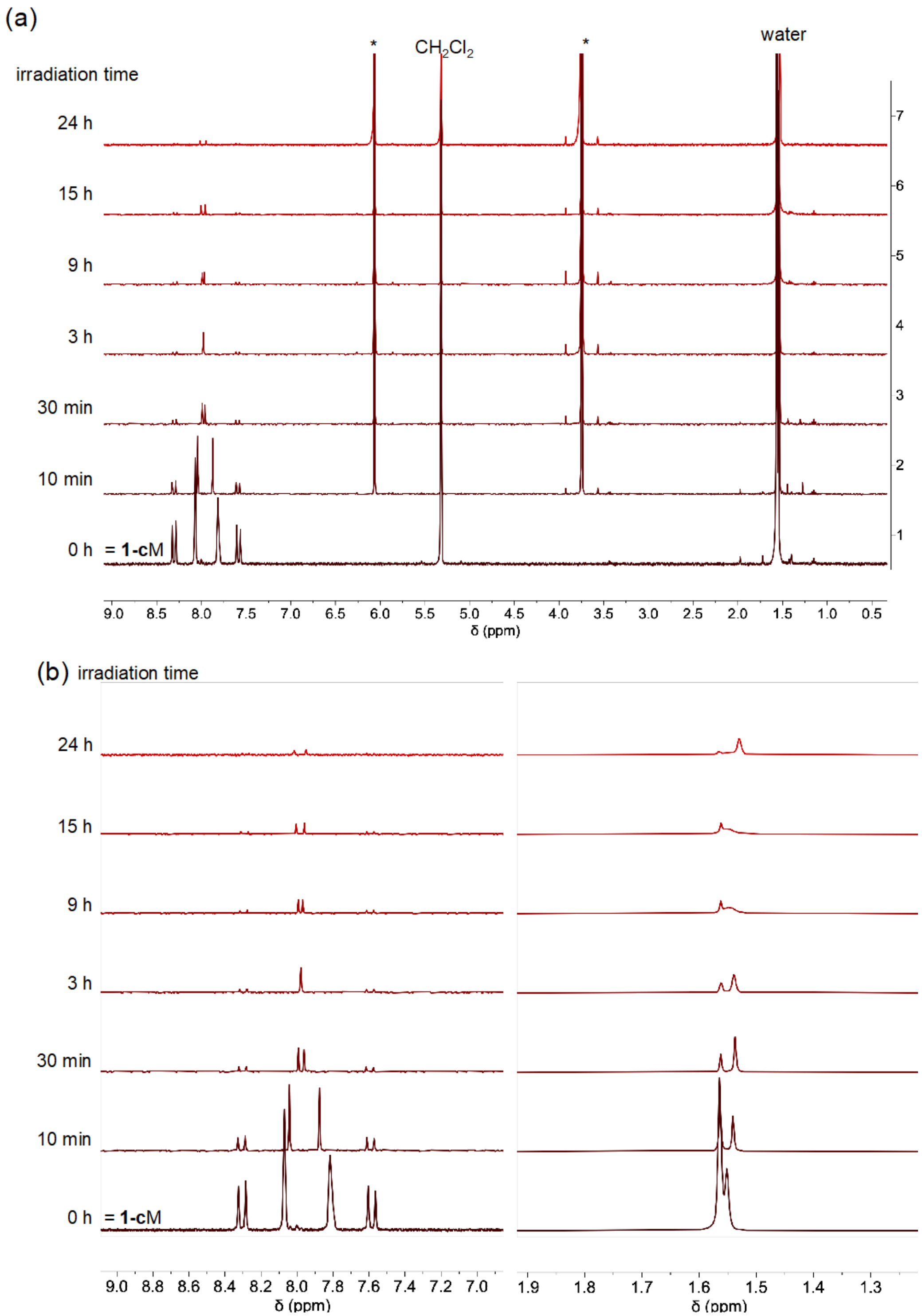

Figure S14. (a) Time-dependent ^{1}H NMR spectra measured during the photopolymerization of **1-**cM, collected in CD_2Cl_2, and (b) the enlarged spectra. Asterisks denote signals of 1,3,5-trimethoxybenzene as an internal standard. The calculation method of monomer conversion (%) can be found in Section 7.

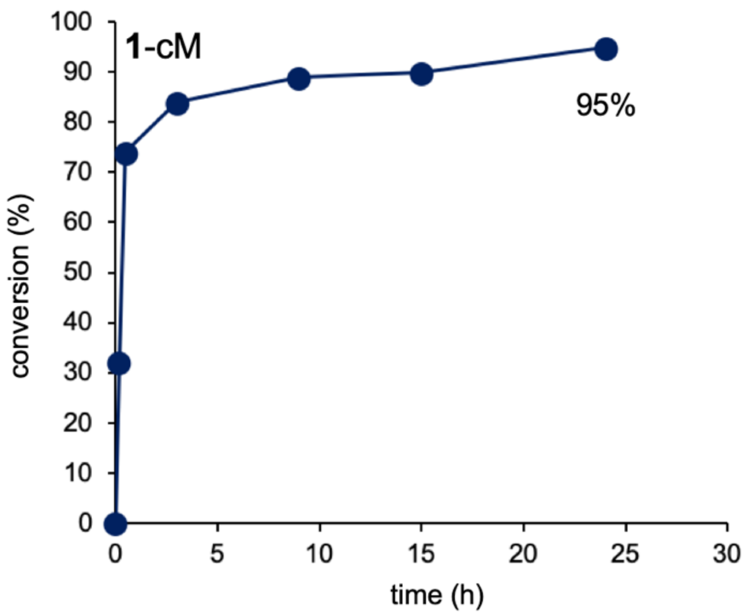

Figure S15. Monomer conversion (%) of **1**-cM over irradiation time, determined by ¹H NMR in CD_2Cl_2. The calculation method of monomer conversion (%) can be found in Section 7.

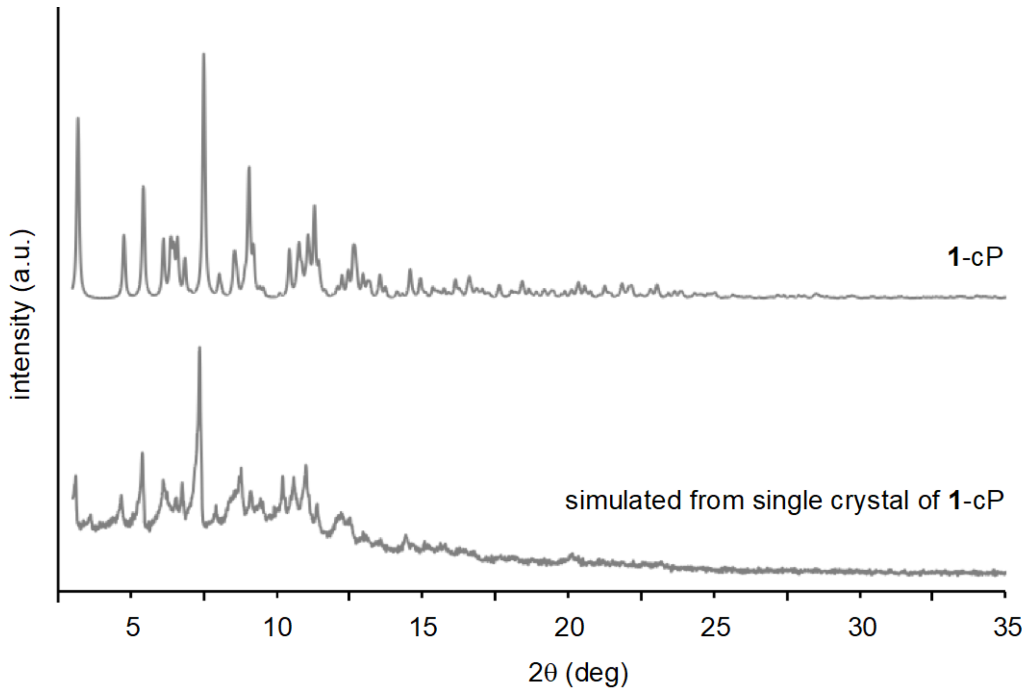

Figure S16. XRD patterns (GaliPIX3D) of powder sample **1**-cP and single crystal **1**-cP (simulated).

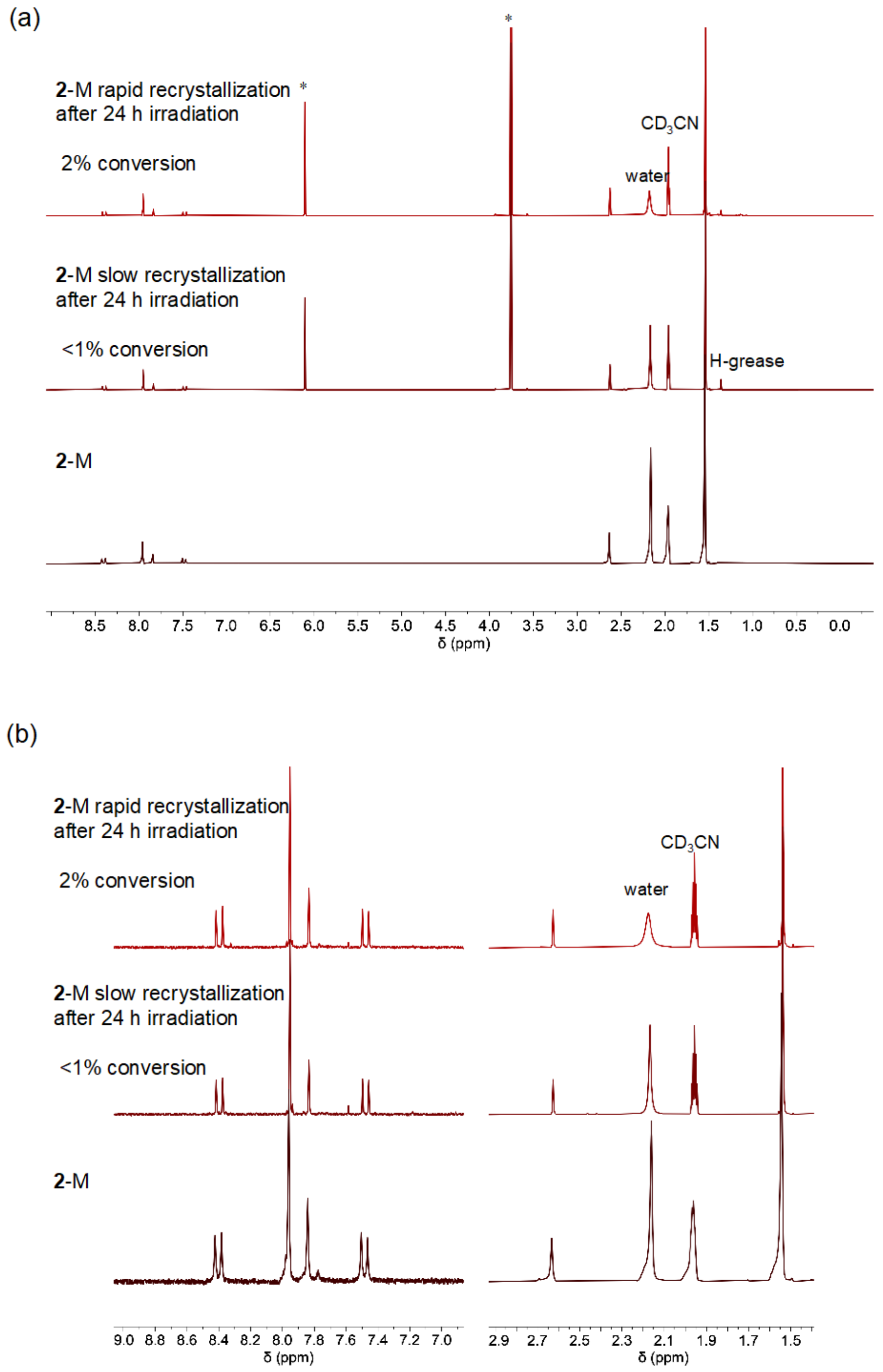

Figure S17. (a) ^{1}H NMR spectra of recrystallized **2**-M collected in CD_3CN: (top) rapid recrystallization followed by the irradiation for 24 hours, (middle) slow recrystallization followed by the irradiation for 24 hours, (bottom) no irradiation. (b) Enlarged spectra. Asterisks denote signals of 1,3,5-trimethoxybenzene as an internal standard. The calculation method of monomer conversion (%) can be found in Section 7.

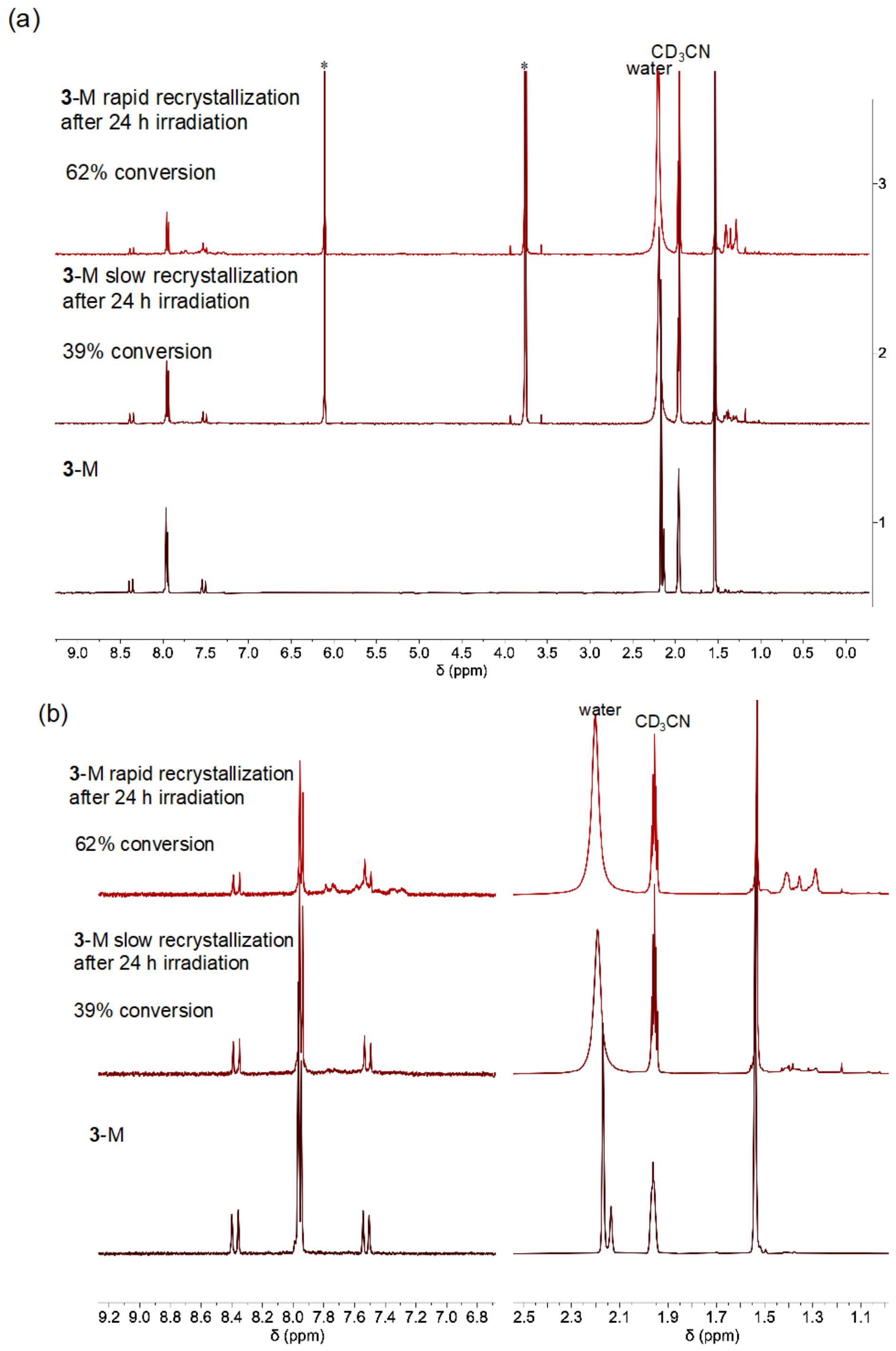

Figure S18. (a) ^{1}H NMR spectra of recrystallized **3**-M collected in CD_3CN: (top) rapid recrystallization followed by the irradiation for 24 hours, (middle) slow recrystallization followed by the irradiation for 24 hours, (bottom) no irradiation. (b) Enlarged spectra. Asterisks denote signals of 1,3,5-trimethoxybenzene as an internal standard. The calculation method of monomer conversion (%) can be found in Section 7.

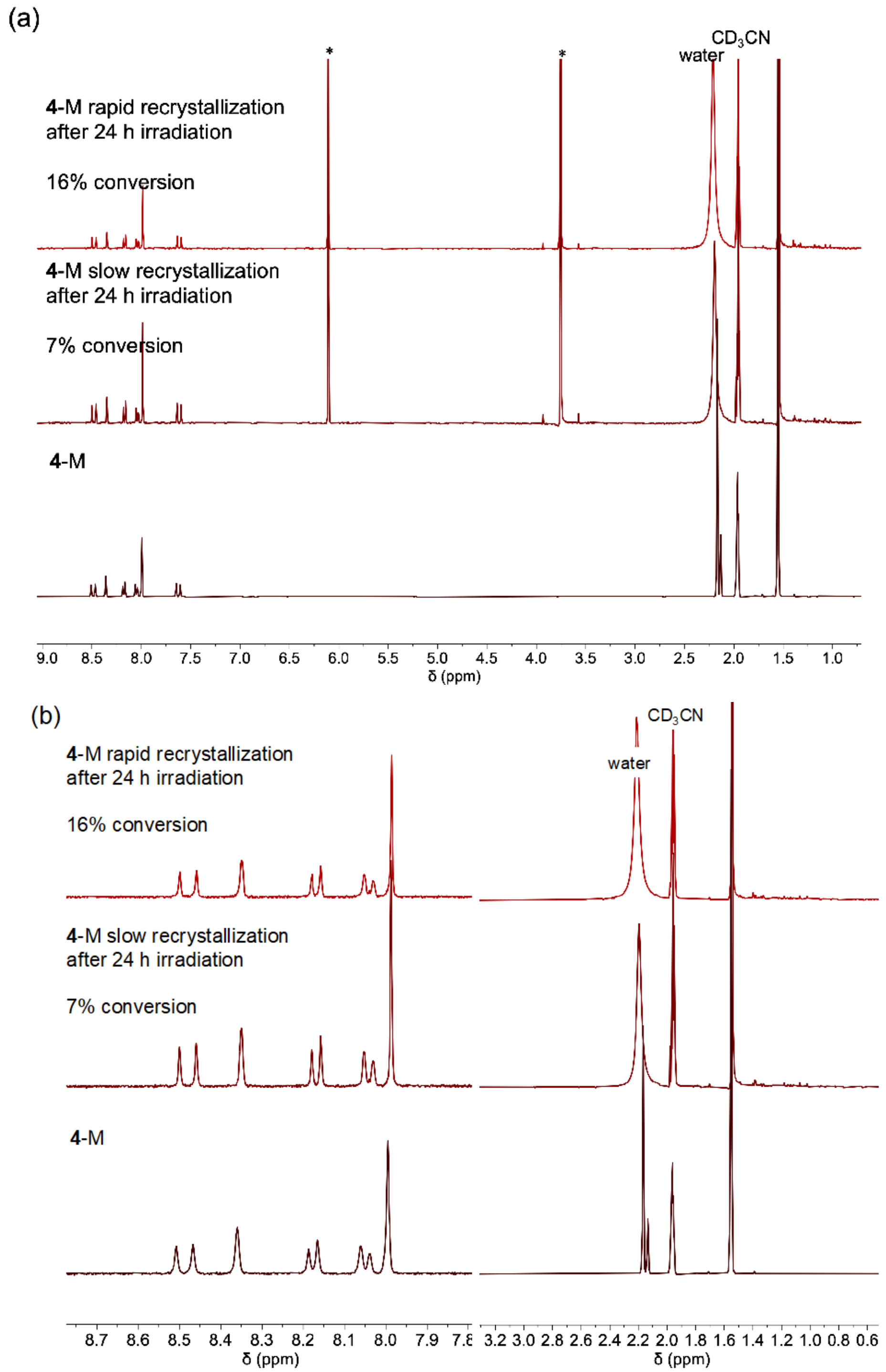

Figure S19. (a) ^{1}H NMR spectra of recrystallized **4**-M collected in CD_3CN: (top) rapid recrystallization followed by the irradiation for 24 hours, (middle) slow recrystallization followed by the irradiation for 24 hours, (bottom) no irradiation. (b) Enlarged spectra. Asterisks denote signals of 1,3,5-trimethoxybenzene as an internal standard. The calculation method of monomer conversion (%) can be found in Section 7.

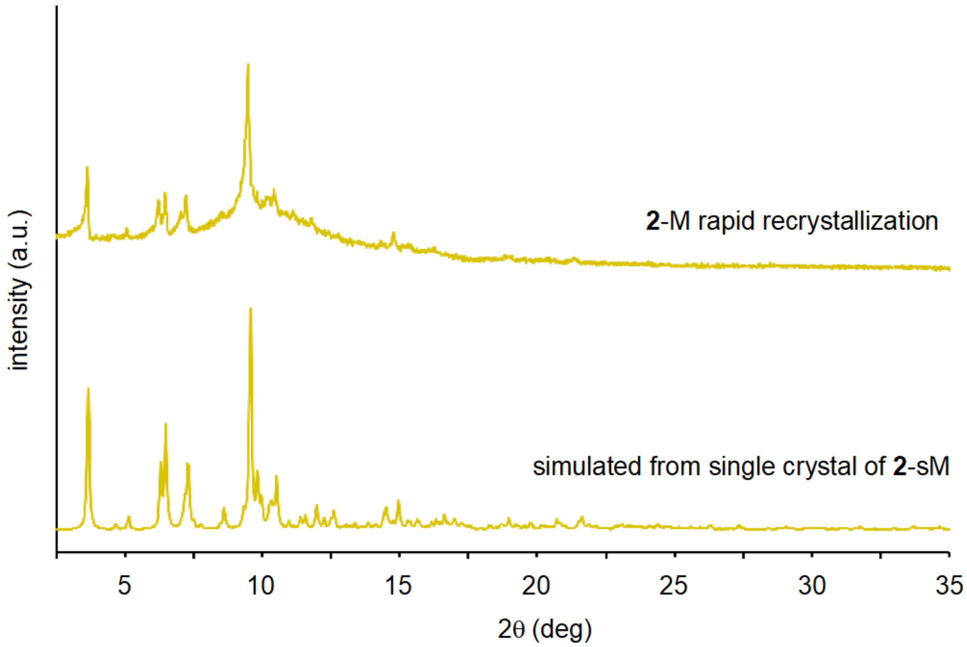

Figure S20. XRD patterns (GaliPIX3D) of rapid recrystallized **2**-M and single crystal **2**-sM (simulated).

8. Mechanical grinding

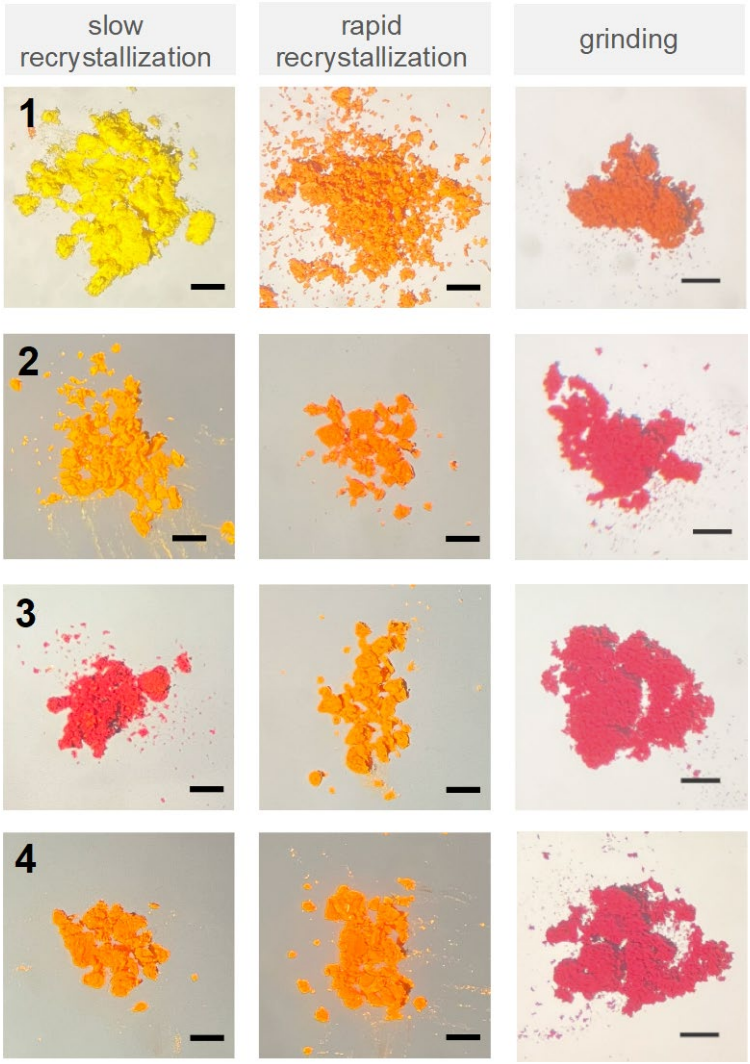

Figure S21. Optical microscope images of **1**–**4**-M that were slowly recrystallized (left), rapidly recrystallized (middle), and mechanically ground (right). Scale bar = 1 mm.

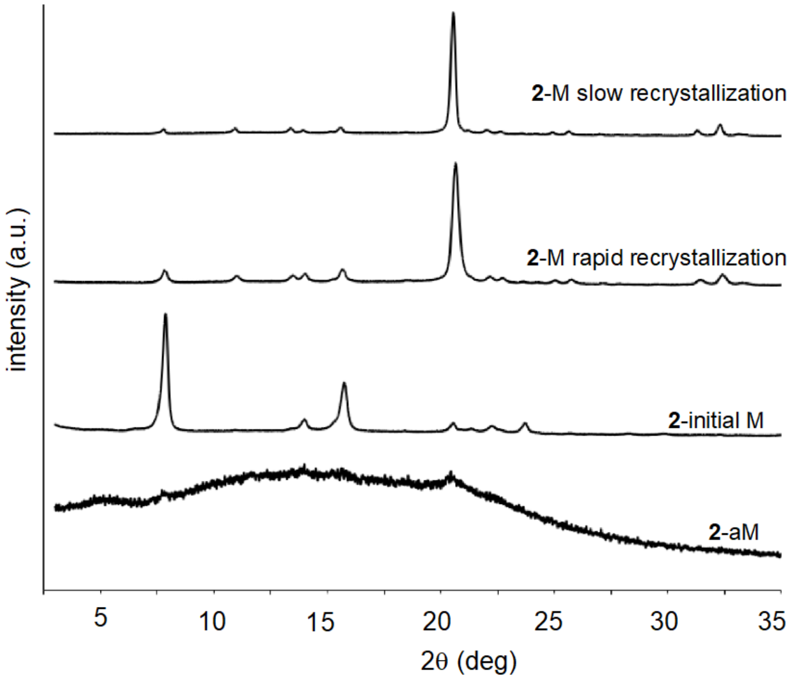

Figure S22. XRD patterns (MiniFlex600) of **2**-M that is either slowly recrystallized, rapidly recrystallized, intact, or mechanically ground.

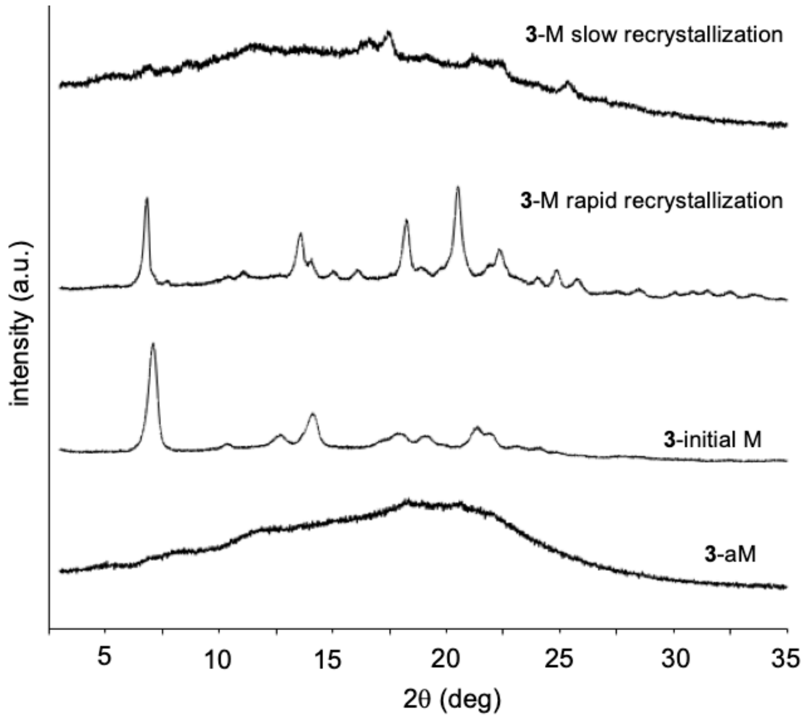

Figure S23. XRD patterns (MiniFlex600) of **3**-M that is either slowly recrystallized, rapidly recrystallized, intact, or mechanically ground.

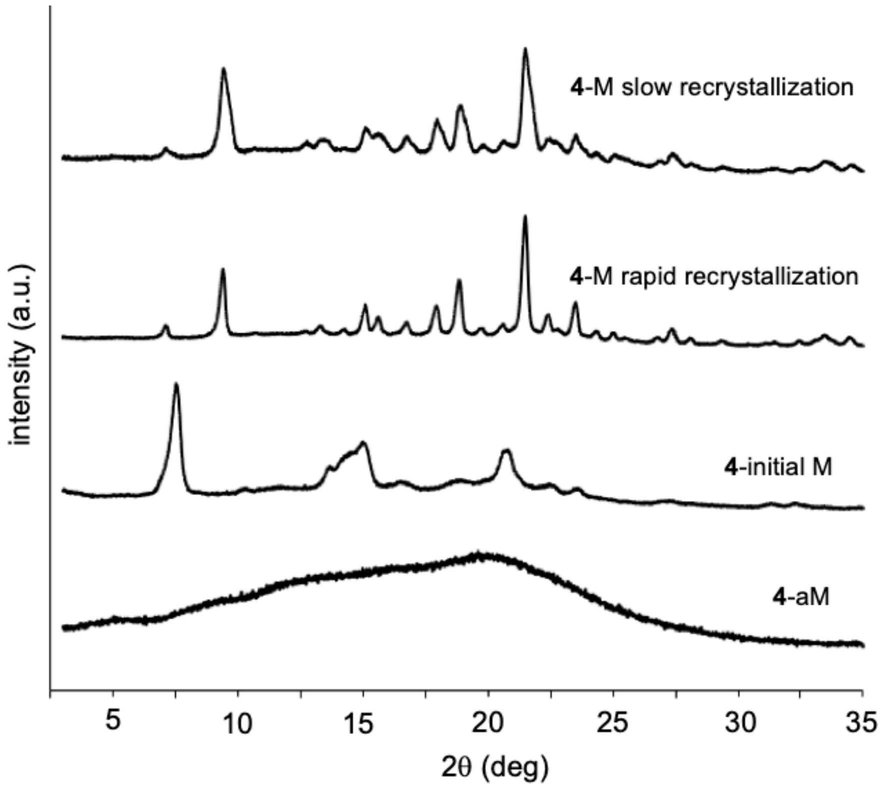

Figure S24. XRD patterns (MiniFlex600) of **4**-M that is either slowly recrystallized, rapidly recrystallized, intact, or mechanically ground.

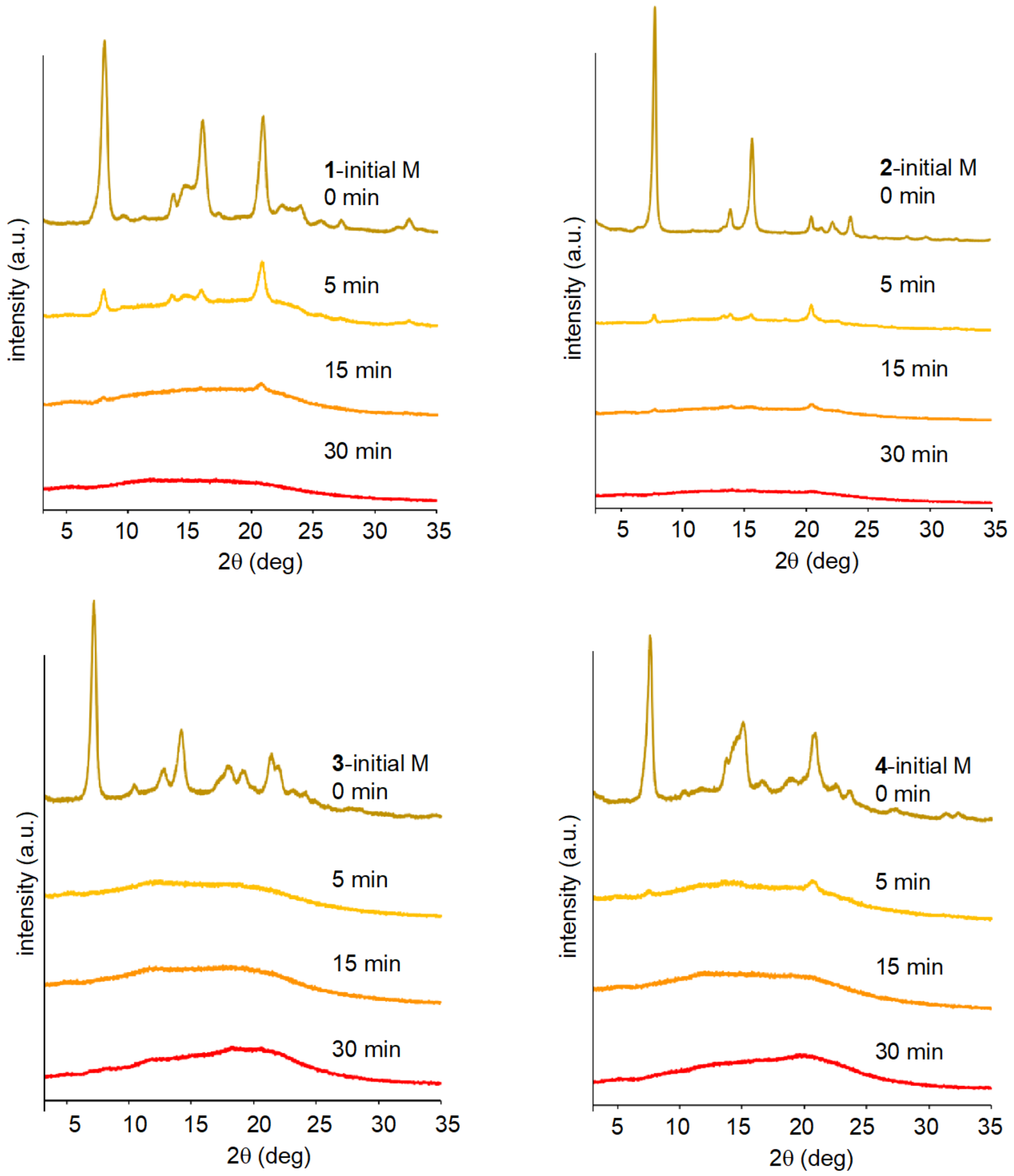

Figure S25. XRD patterns (MiniFlex600) of **1**–**4**-M obtained after grinding for varied durations.

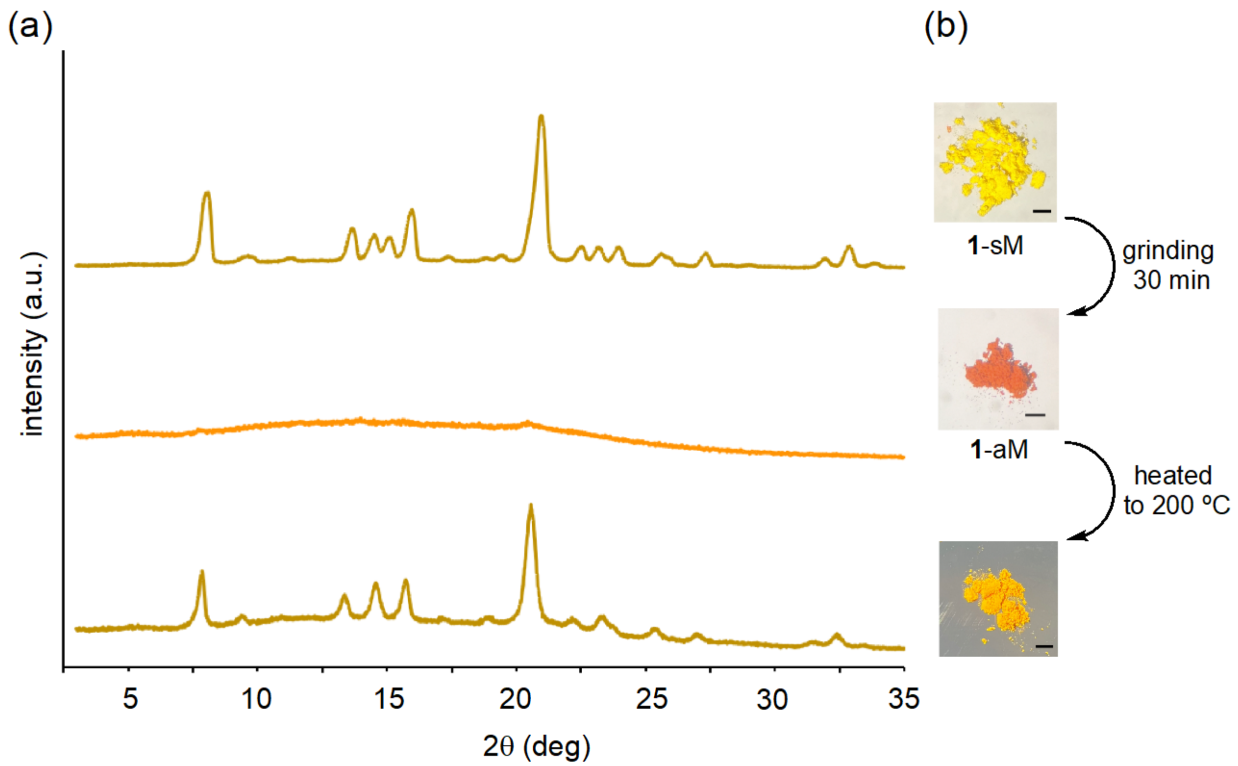

Figure S26. (a) PXRD patterns (MiniFlex600) and (b) optical microscope images of compound **1**-sM (top), **1**-aM (middle), and **1**-aM after heating to 200 °C (bottom) (Scale bar = 1 mm) showing the restoration of crystallinity of **1**-aM upon heating.

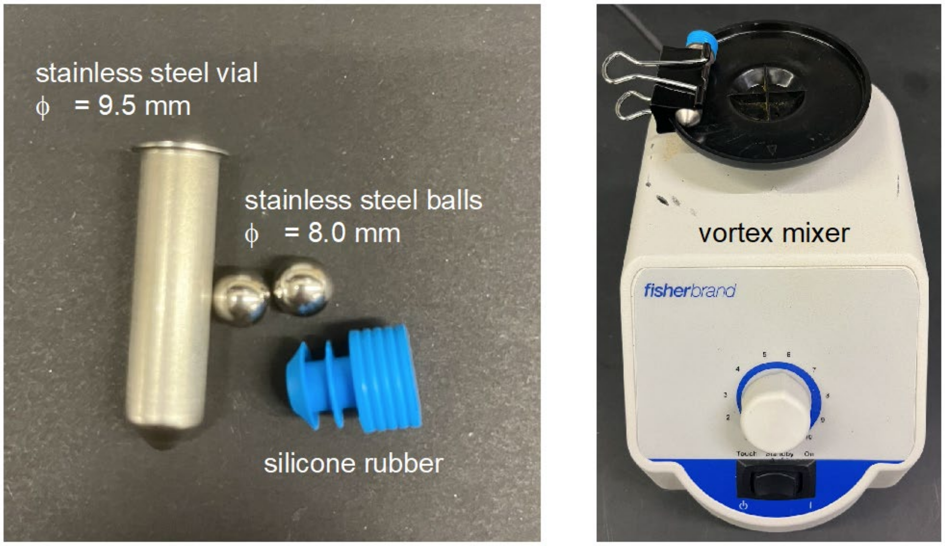

Figure S27. Photographs of equipment setup for ball milling and a vortex mixer

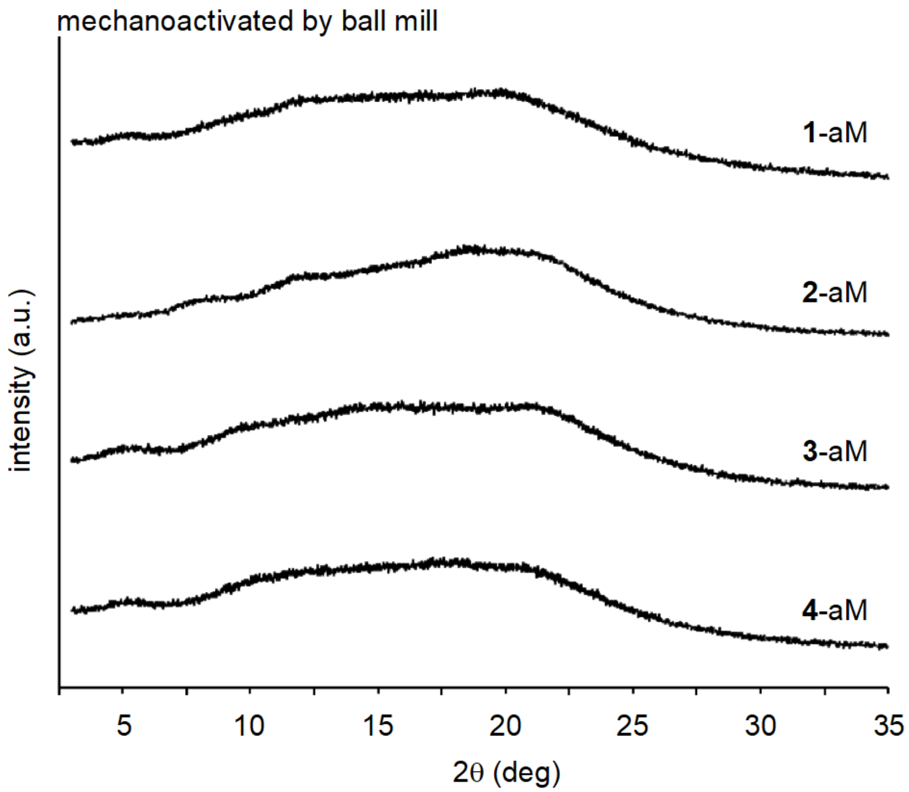

Figure S28. PXRD patterns (MiniFlex600) of **1**–**4**-aM prepared by ball milling.

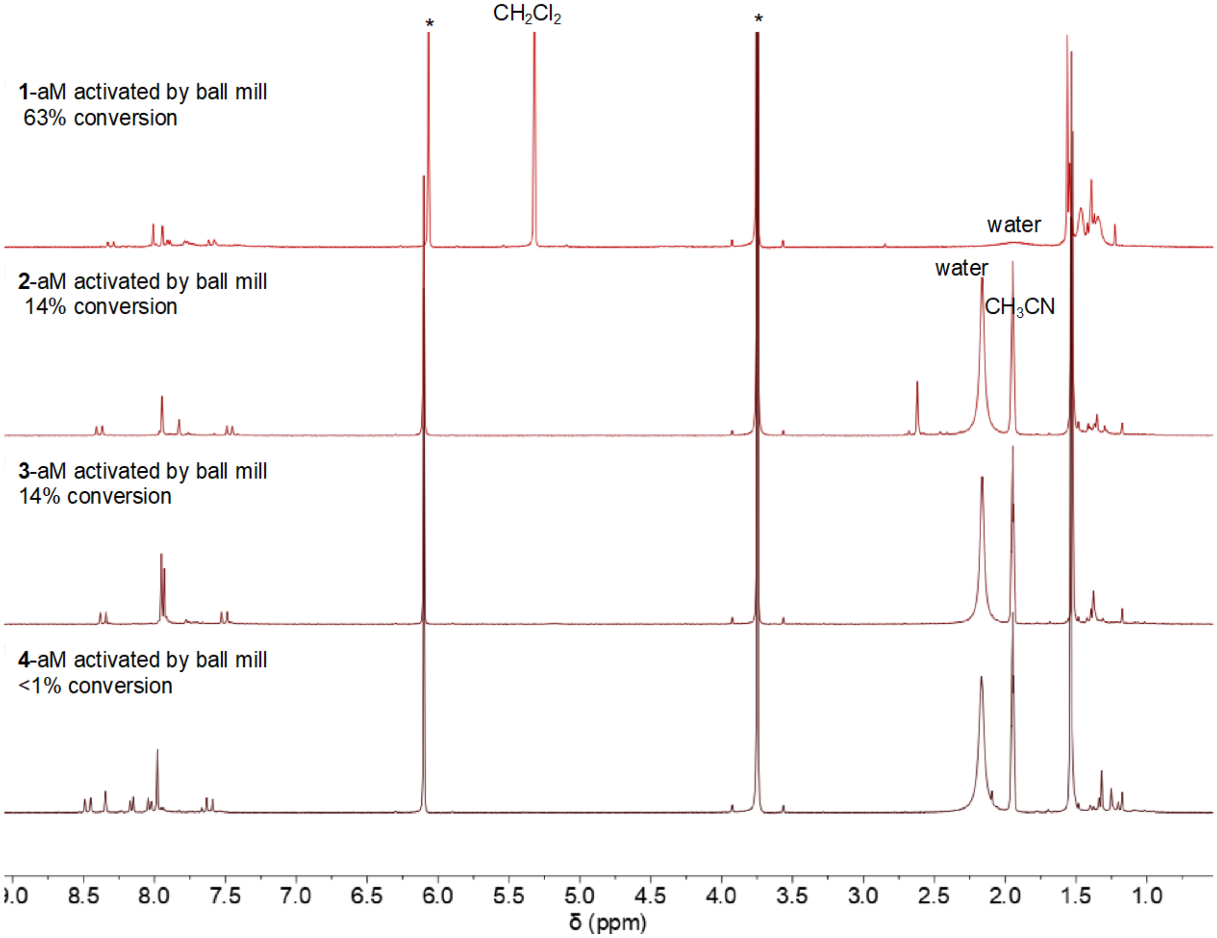

Figure S29. ^{1}H NMR spectra of ball-milled **1**–**4**-aM after 24 hours of irradiation, measured in CD_2Cl_2 (for **1**-aM) or CD_3CN (for **2**–**4**-aM). Asterisks denote the signals of 1,3,5-trimethoxybenzene as an internal standard. The calculation method of monomer conversion (%) can be found in Section 7.

Table S2. Conversion (%) of monomers upon irradiation at 470 nm for 24 h in solid state, analyzed by ^{1}H NMR.

	monomer activation	
compound	grinding in a mortar	ball milling
**1**	100	63
**2**	76	14
**3**	100	14
**4**	58	<1

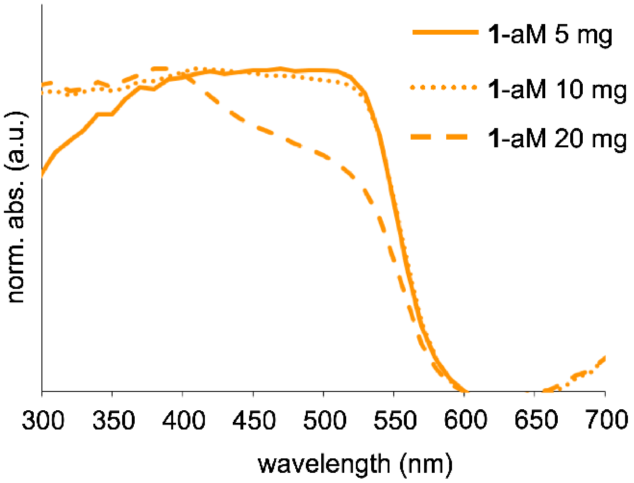

Figure S30. Diffuse reflection spectra of **1**-aM with varied sample amount.

9. TD-DFT calculation results

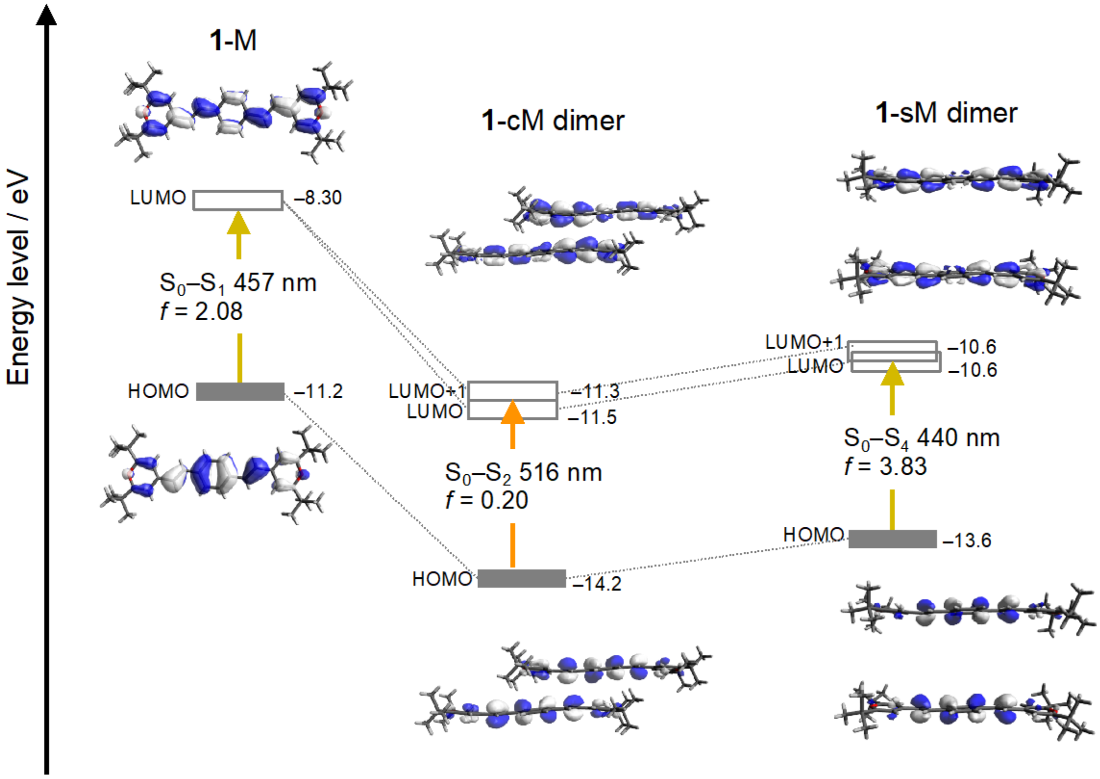

Figure S31. Orbital energy diagrams, pictorial representations of the selected Khon–Sham molecular orbitals, and TD-DFT vertical excitation energies with oscillator strengths obtained for **1**-cM, stacked **1**-cM pair (dimer), and stacked **1**-sM pair (dimer). Transitions with the least energy among the allowed transitions were shown. The dimer structure was extracted from crystal geometries and calculated at the (TD)B3LYP/6-31G** level of theory.

10. Fluorescence emission spectra

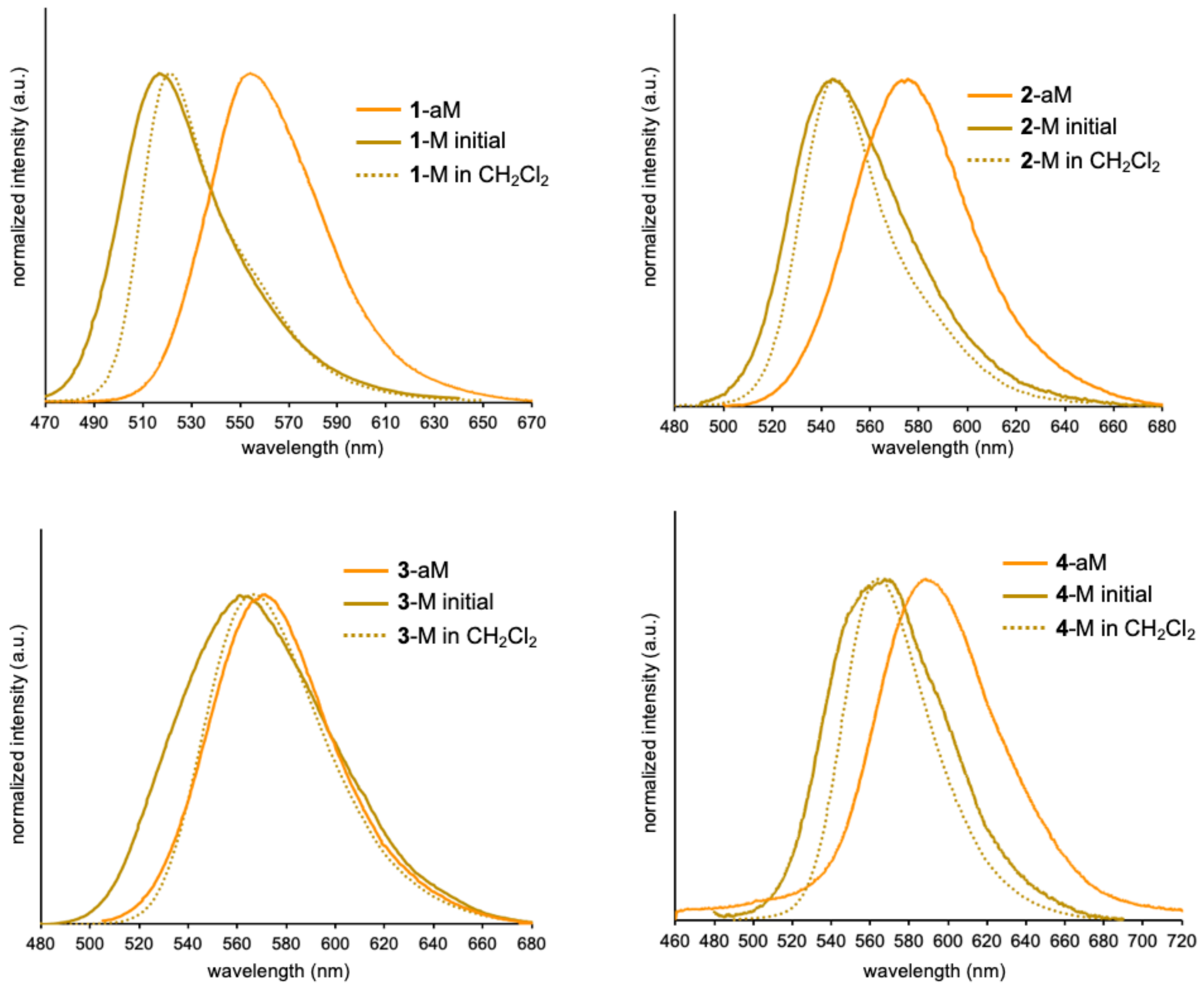

Figure S32. Emission spectra of **1**–**4**-M solutions in CH_2Cl_2 (yellow dashed line), dispersions in diethyl ether (yellow solid line), and **1**–**4**-aM (after grinding) dispersions in diethyl ether (orange solid line).

11. Solid-state NMR spectra

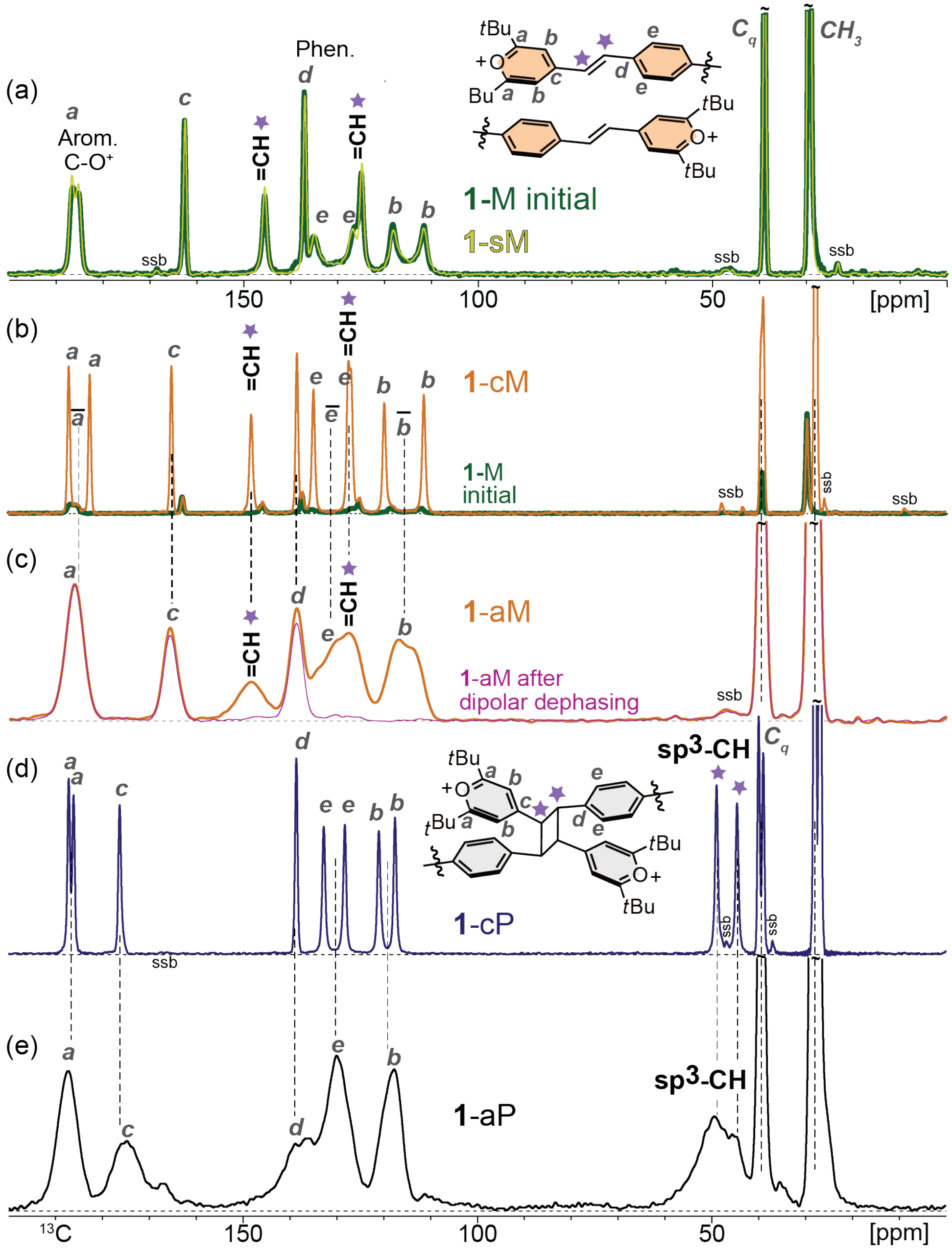

Figure S33. Nearly quantitative solid-state ^{13}C NMR spectra of six materials derived from compound **1**-M. The intense peaks of the *t*-butyl groups have been clipped. (a) Spectra of **1**-M initial and **1**-sM superimposed. The crystal packing is identical, but **1**-M initial also contains a few percent of a minor component resembling **1**-aM (e.g. contributing a foot at 140 ppm). (b) Spectrum of **1**-cM (orange trace), with the spectrum of **1**-M initial (thick green trace) superimposed scaled to match eight small peaks observed. Significant chemical shift differences between the majority phase of **1**-cM (orange trace) and **1**-M are apparent, reflecting their different packing. For the

crystalline compounds in a) and b), the small number of peaks shows that the asymmetric unit cell contains a single molecule that has inversion symmetry. The assignment of the olefin peaks (different magenta stars) is based on chemical-shift predictions. (c) Spectrum of **1**-aM (orange trace), with the spectrum after dipolar dephasing of nonprotonated and mobile carbons (thin purple trace) superimposed. Dipolar dephasing was also applied for the other samples (not shown) and consistently shows that the signal at 140 ppm is from substituted aromatic C and that near 147 ppm (if present) from =C-H. Vertical dashed lines highlight that the average chemical shifts of corresponding sites in **1**-cM and **1**-aM are in good agreement, indicating similar packing, while at least four signals of **1**-M initial and **1**-sM have distinctly different positions. (d) Spectrum of **1**-cP. The assignment of the cyclobutane peaks (different magenta stars) is based on the ^{1}H-^{13}C HetCor spectrum in Figure S34. (e) Spectrum of **1**-aP. ”ssb”: spinning sideband.

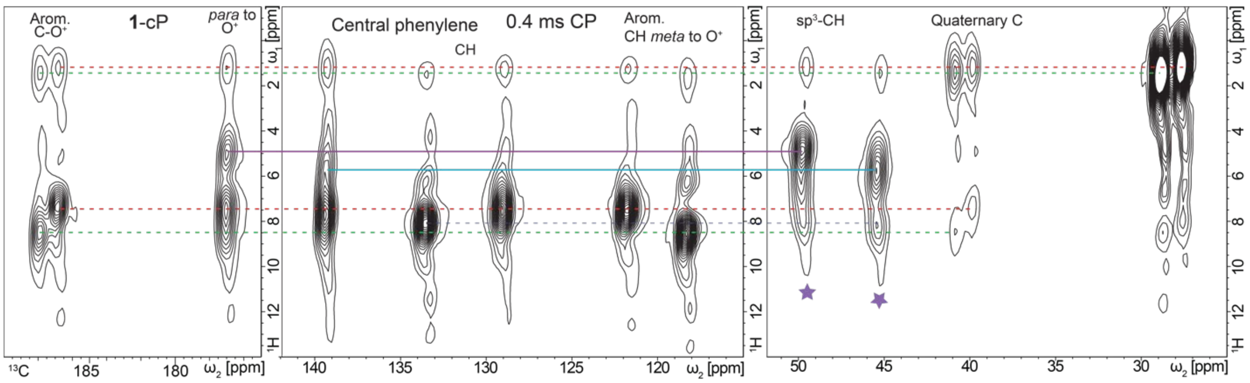

Figure S34. Two-dimensional ^{1}H-^{13}C HetCor spectrum of **1**-cP with 0.4-ms cross-polarization time. Solid horizontal lines connect cross peaks that enable assignment of the cyclobutane ^{13}C NMR signals at 45.5 and 50 ppm. The 50-ppm carbon is bonded to a 5-ppm proton that also polarizes the 177-ppm carbon of the O-containing ring, while the 45.5-ppm carbon is bonded to a 5.7-ppm proton that also polarizes the substituted phenylene carbon resonating at 140 ppm.

.

12. Photoinduced conversion in solution and in amorphous state

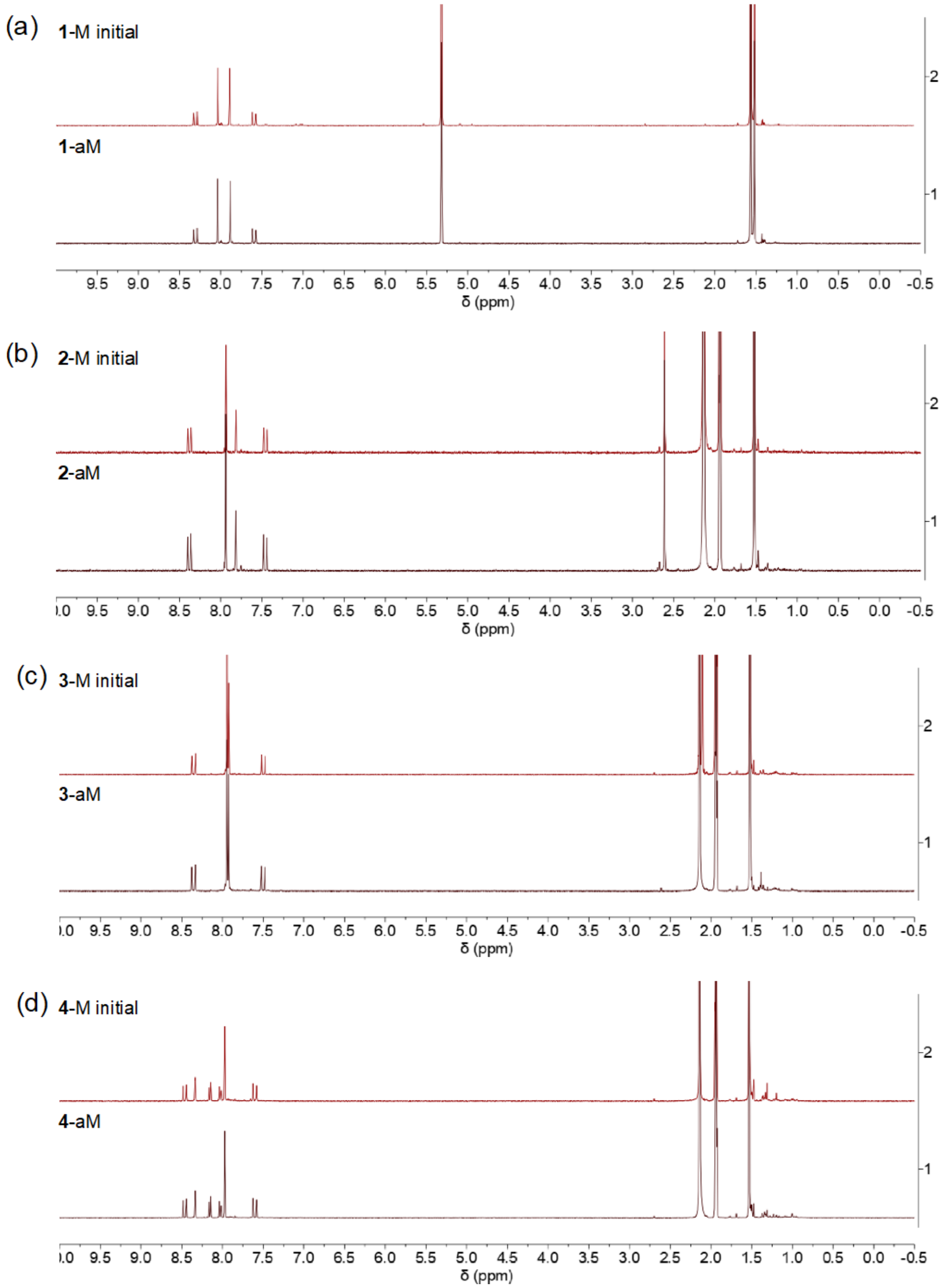

Figure S35. ^1H NMR spectra of **1**–**4**-M initial (top) and **1**–**4**-aM (bottom) (a) **1**-M in CD_2Cl_2. (b) **2**-M in CD_3CN. (c) **3**-M in CD_3CN. (d) **4**-M in CD_3CN.

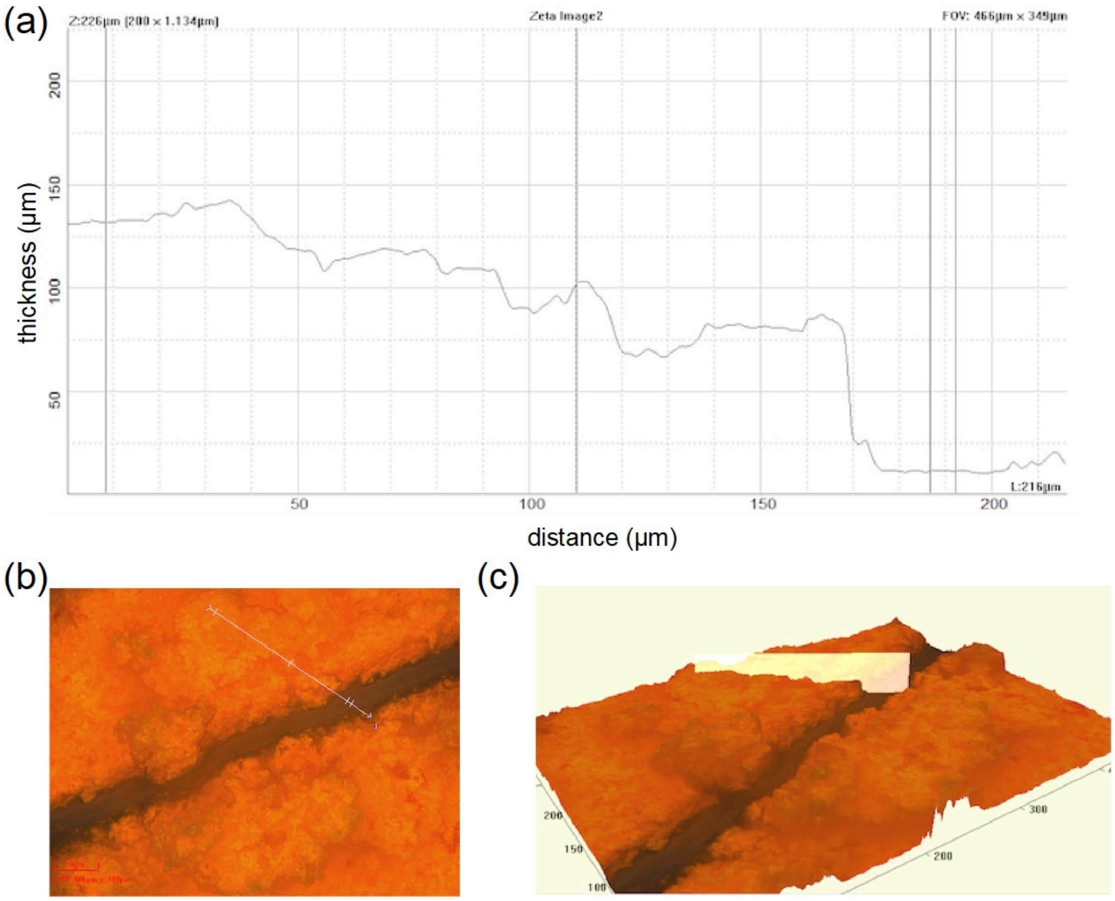

Measurement	1	2	3	4	5	6	7	8	Average
Thickness (µm)	72.1	222.1	173.6	107.2	86.7	107.0	61.4	125.1	**119.4**

Figure S36. (a) Thickness measurement of a sandwich film of compound **1**. (b) An optical microscope image of the measure area. (c) 3D topography of the measured area. Eight different areas were measured to get the average thickness of 119 µm

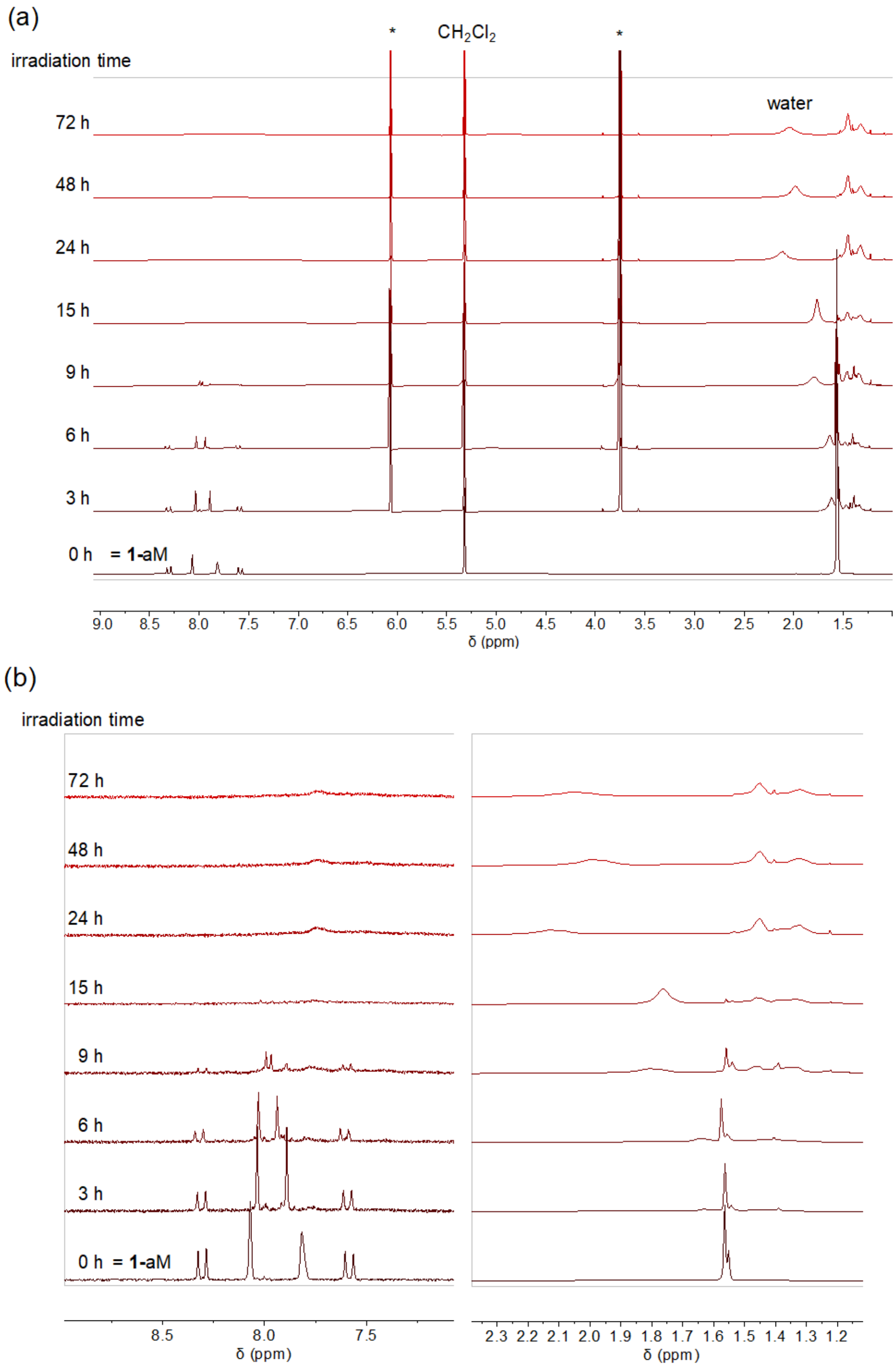

Figure S37. (a) Time-dependent ^{1}H NMR spectra measured during the photopolymerization of **1**-aM, collected in CD_2Cl_2, and (b) the enlarged spectra. Asterisks denote signals of 1,3,5-trimethoxybenzene as an internal standard. The calculation method of monomer conversion (%) can be found in Section 7.

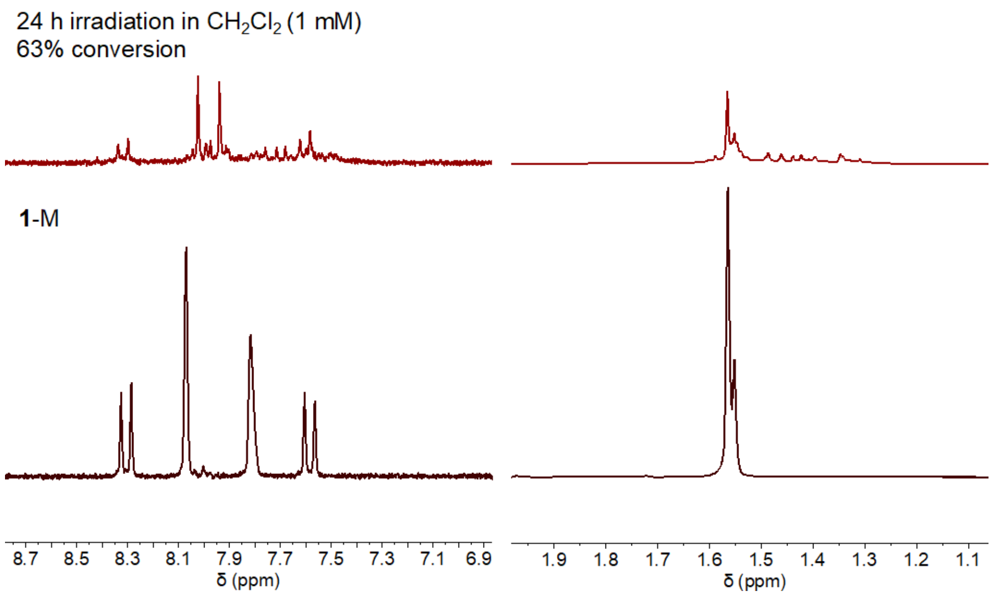

Figure S38. ^{1}H NMR spectra of **1**-M after 24 h irradiation in 1 mM CH_2Cl_2 solution (top) and the initial **1**-M (bottom).

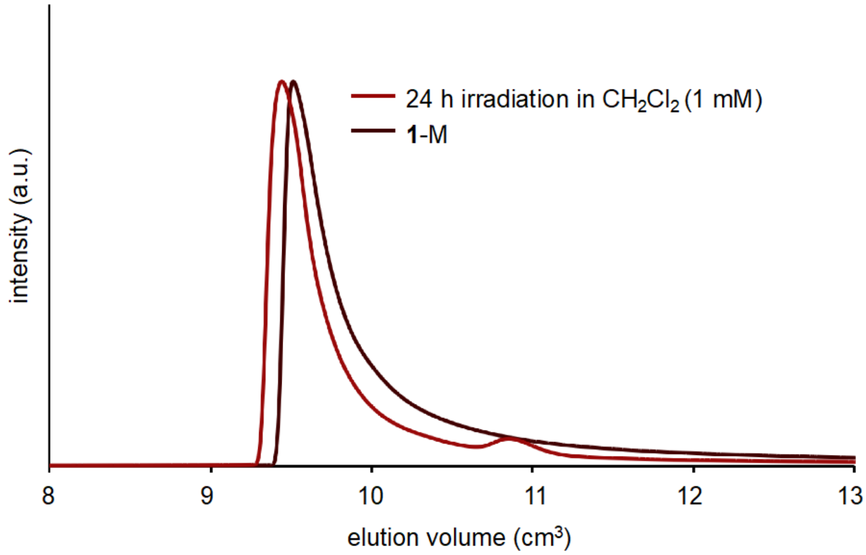

Figure S39. SEC plots of **1**-M after 24 h irradiation in 1 mM CH_2Cl_2 solution (brown) and the initial **1**-M (black).

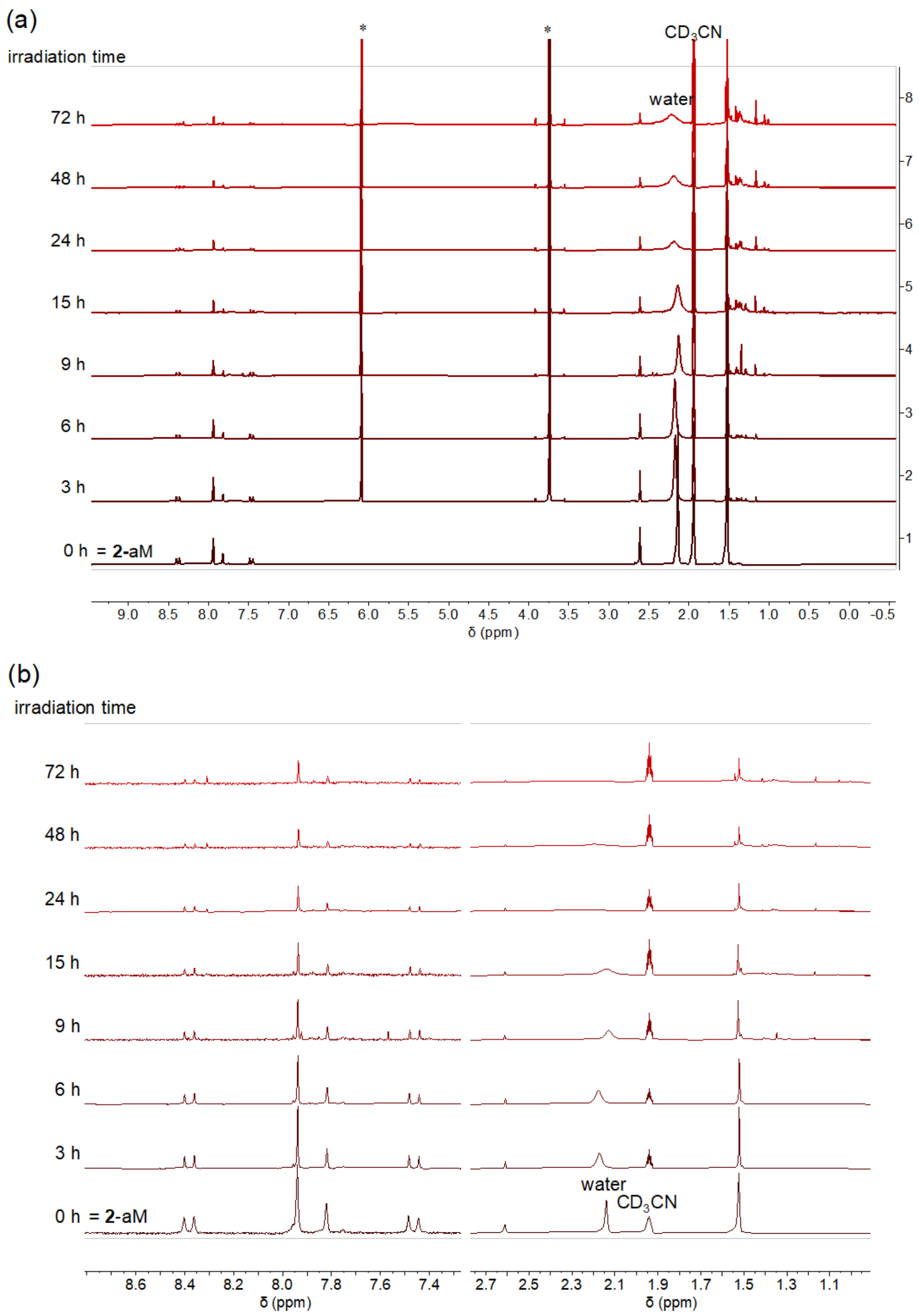

Figure S40. (a) Time-dependent ^{1}H NMR spectra measured during the photopolymerization of **2**-aM, collected in CD_3CN, and (b) the enlarged spectra. Asterisks denote signals of 1,3,5-trimethoxybenzene as an internal standard. The calculation method of monomer conversion (%) can be found in Section 7.

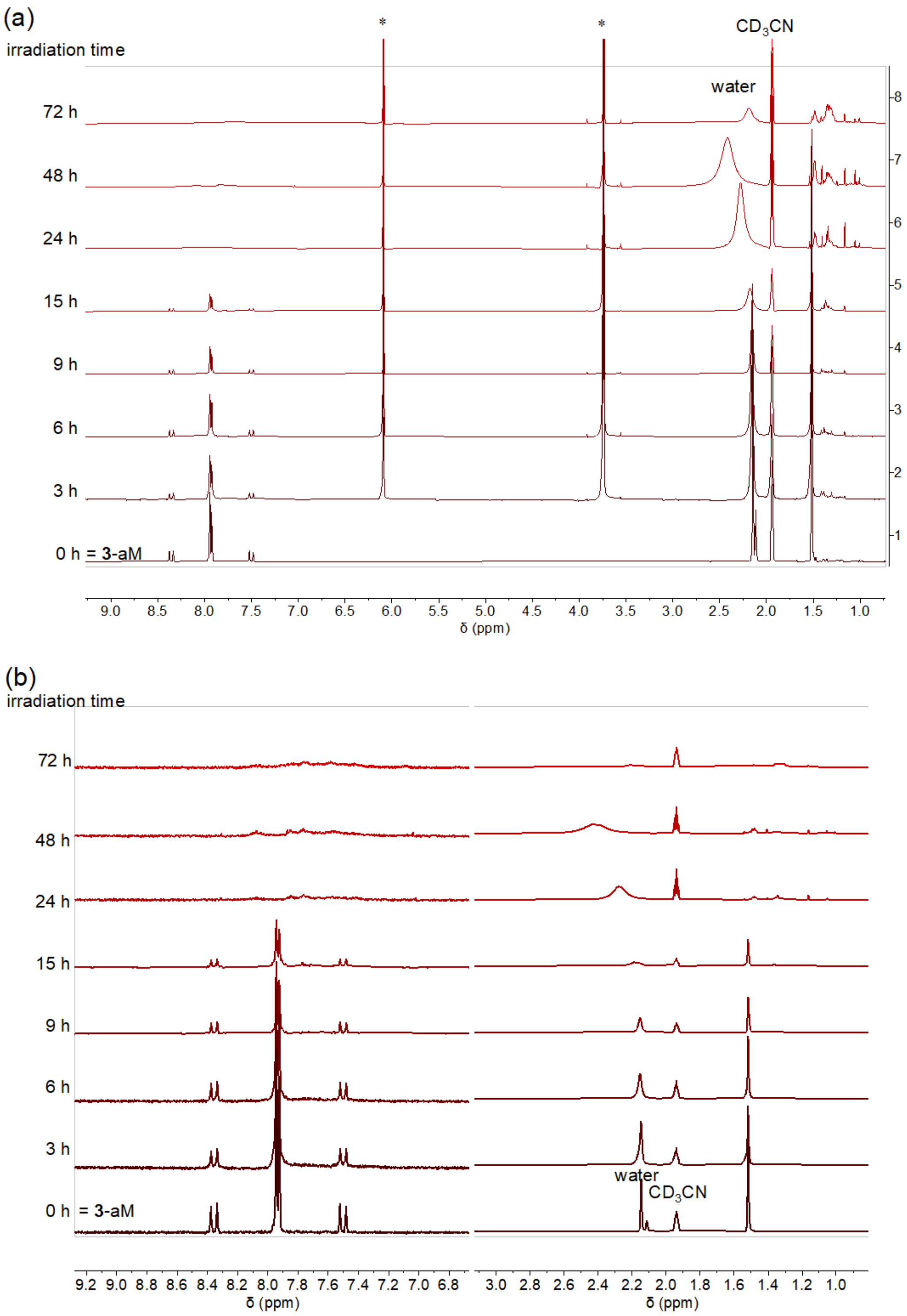

Figure S41. (a) Time-dependent ^{1}H NMR spectra measured during the photopolymerization of **3-aM**, collected in CD_3CN, and (b) the enlarged spectra. Asterisks denote signals of 1,3,5-trimethoxybenzene as an internal standard. The calculation method of monomer conversion (%) can be found in Section 7.

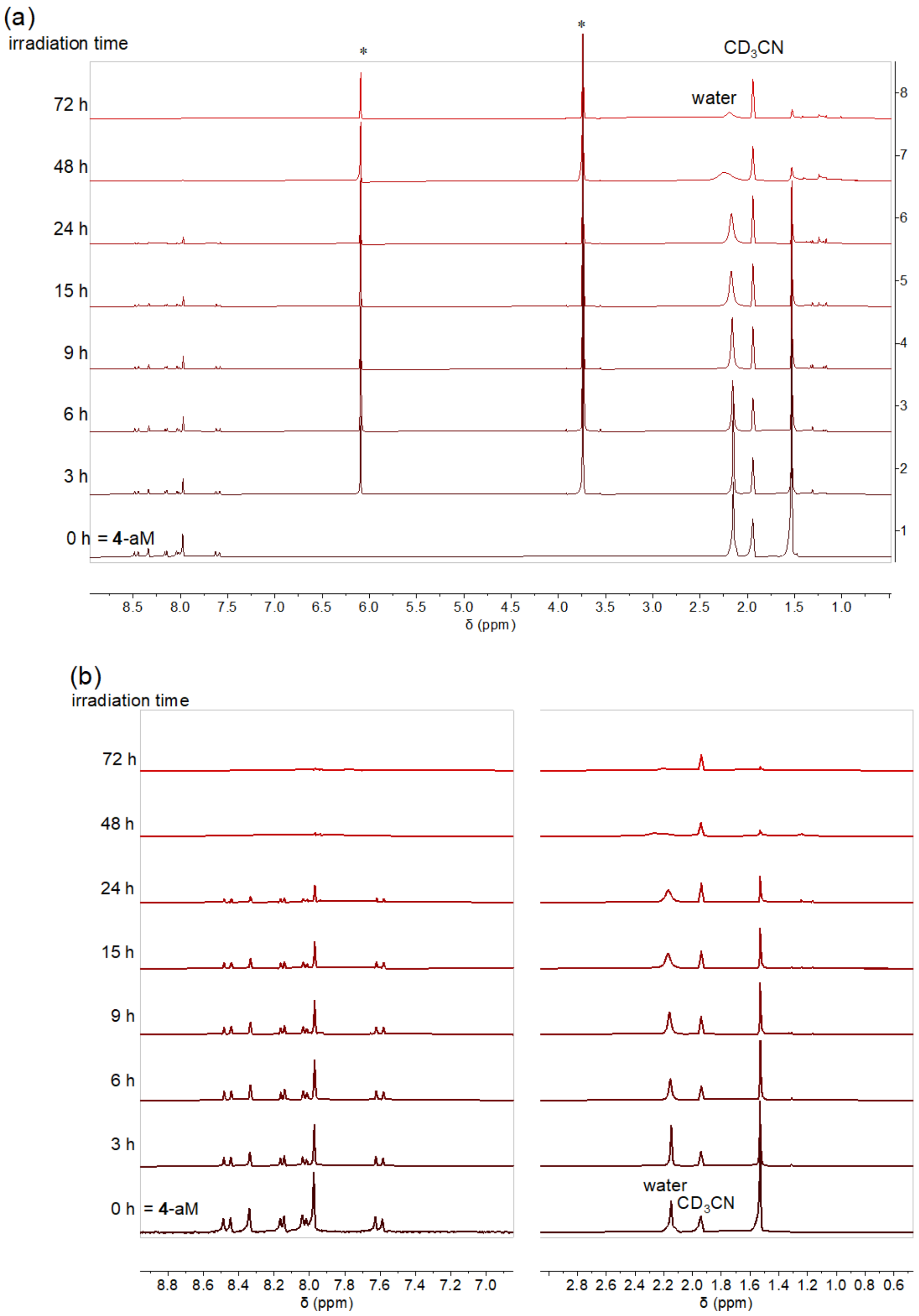

Figure S42. (a) Time-dependent ^{1}H NMR spectra measured during the photopolymerization of **4-aM**, collected in CD_3CN, and (b) the enlarged spectra. Asterisks denote signals of 1,3,5-trimethoxybenzene as an internal standard. The calculation method of monomer conversion (%) can be found in Section 7.

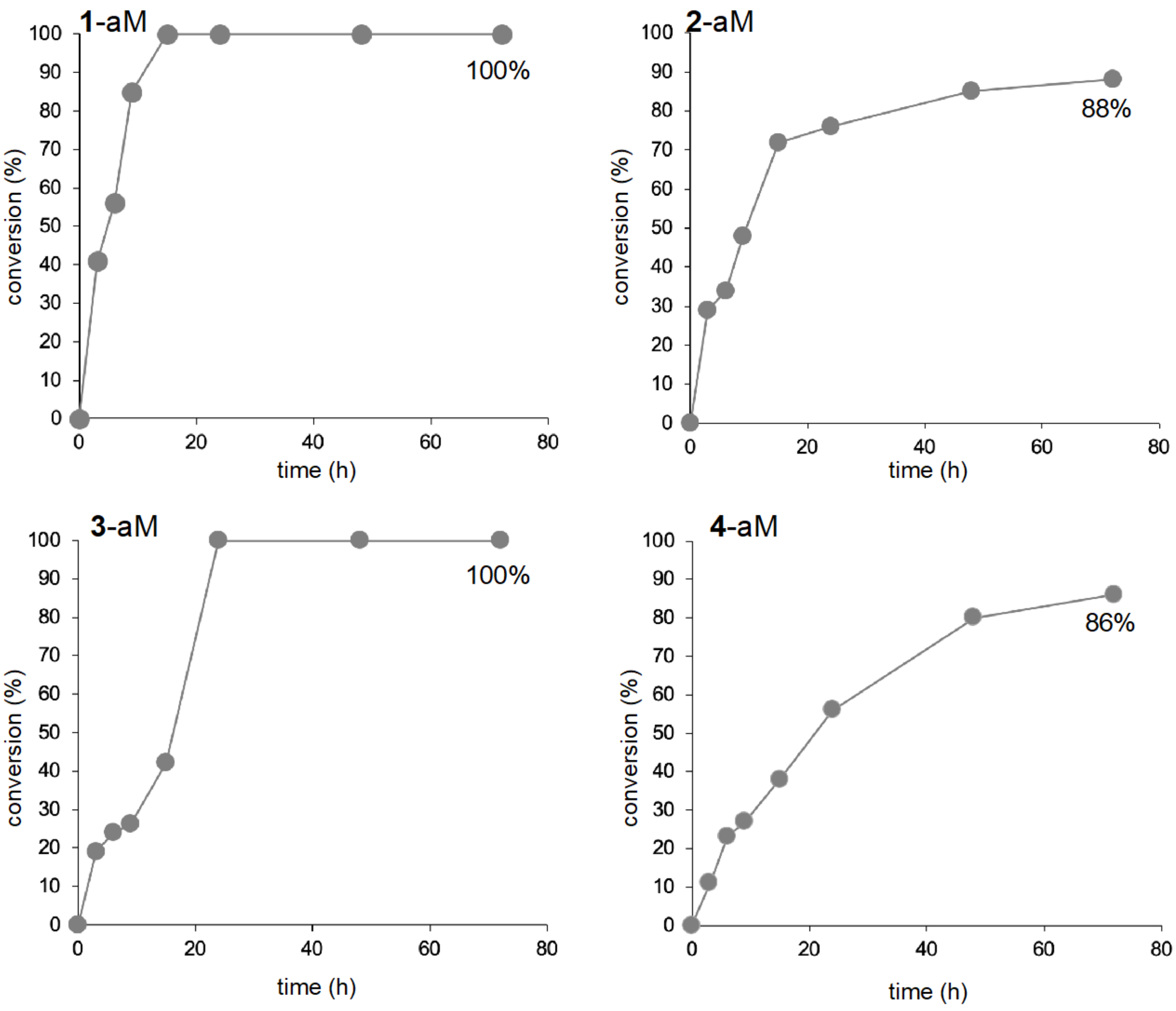

Figure S43. Monomer conversion (%) of **1**–**4**-aM over irradiation time, determined by ^{1}H NMR. The calculation method of monomer conversion (%) can be found in Section 7.

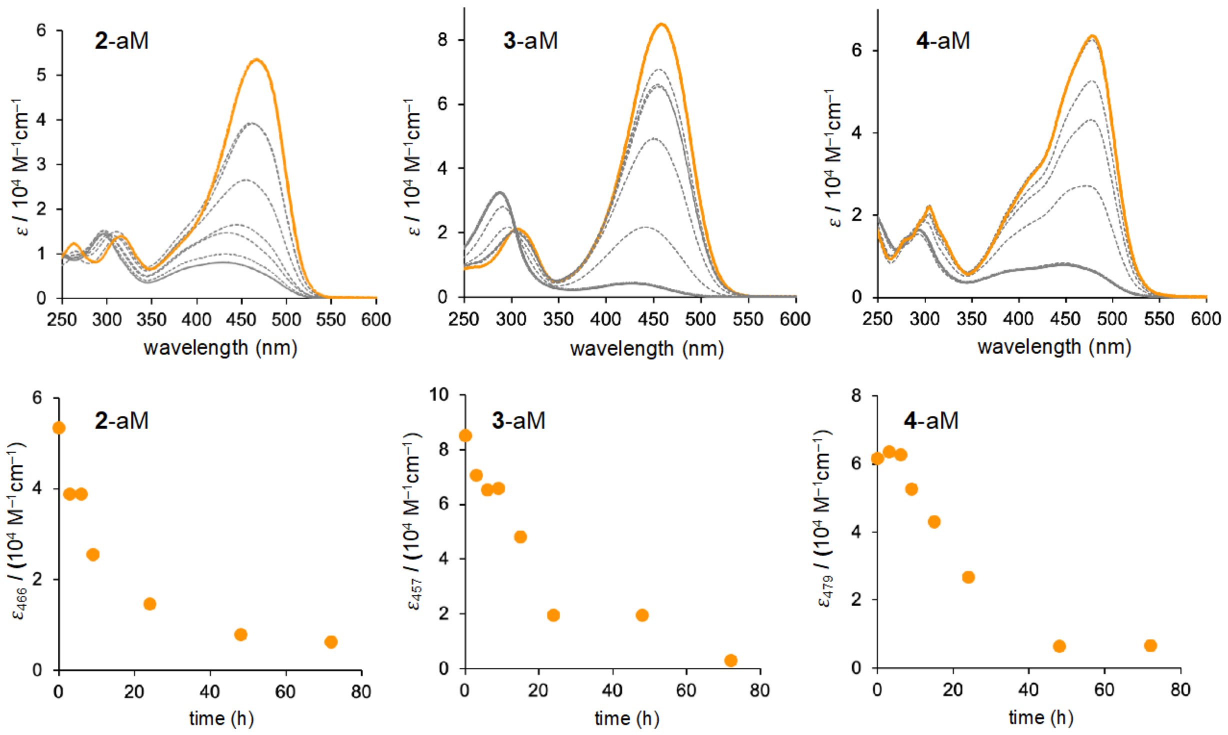

Figure S44. (top) UV-vis absorption spectra of acetonitrile dissolved aliquots of **2**–**4**-aM before (solid orange), during (dotted gray), and after 72-hour irradiation (solid gray) at 470 nm. (bottom) Changes of the peak molar extinction coefficients of **2**–**4**-aM over irradiation time.

13. Static light penetration depth measurement

The calculation of static light penetration depth of **1**-M and **1**-aP in solid state was based on a reported method.[3] Theoretically, the static penetration depths (δ_p) are estimated by

$$\delta_p = d_{sol}\, n_{sol} \,/\, A_{sol}\, n_{film} \quad \text{-----eq.4}$$

where A_{sol}, is the absorbance of **1**-M or **1**-aP in CH_2Cl_2 solution, d_{sol} is the thickness of the solution, n_{sol} is the molar fraction in CH_2Cl_2, n_{film} is the molar fraction in a sandwiched film, which equals to 1.

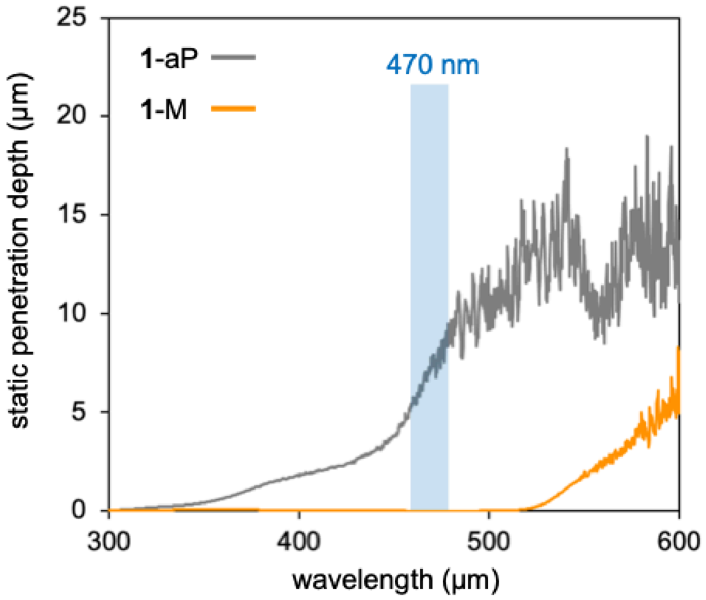

Figure S45. The estimated light penetration depth of **1**-M and **1-**aP in solid state. The blue highlighted area indicates the penetration depths of wavelengths around 470 nm.

14. Molecular weights and solubilities of polymers

Table S3. Weight average molecular weight (M_w), number average molecular weight (M_n), peak top molecular weight (M_p), and polydispersity index (PDI) of **1**-aP measured by SEC.

entry	wavelength (nm)	irradiation time (h)	M_w	M_n	M_p	PDI
1	470	24	8.8×10^2	241	1212	3.6
2	470	48	2.8×10^3	331	1723	8.5
3	470	72	1.8×10^4	468	2395	38.4
4*	470	72	2.5×10^5	654	3721	388
5	530	72	4.6×10^3	503	1657	9.2

* Performed on a larger irradiation area (10 cm x10 cm glass slide).

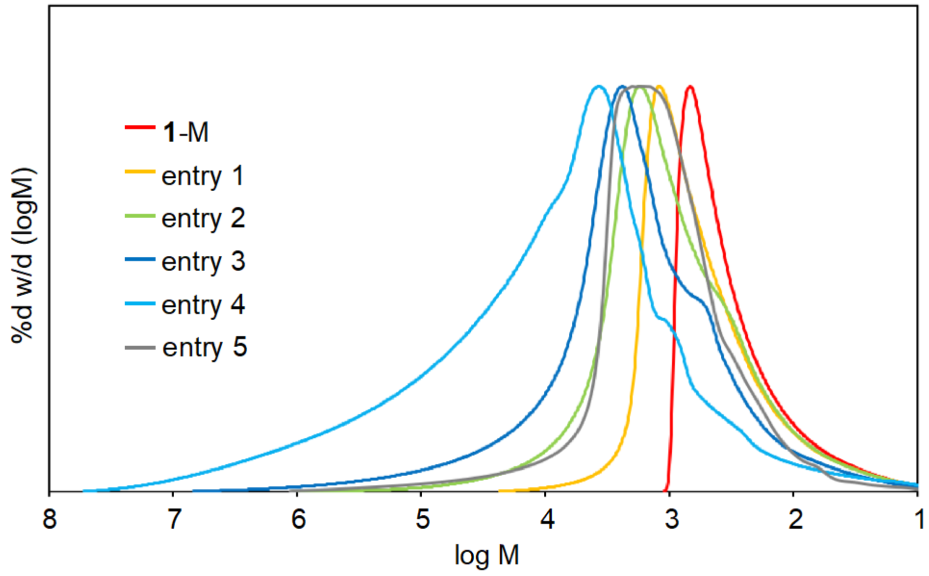

Figure S46. Differential molecular weight curves of compound **1**, which corresponds to data in Table S4.

Table S4. Weight average molecular weight (M_w), number average molecular weight (M_n), peak top molecular weight (M_p), and polydispersity index (PDI) of **2**–**4**-aP (irradiated for 72 hours) measured by SEC.

	M_w	M_n	M_p	PDI
**2**-aP	1.6×10^3	375	922	2.5
**3**-aP	6.7×10^4	217	972	309
**4**-aP	1.5×10^3	641	1808	2.3

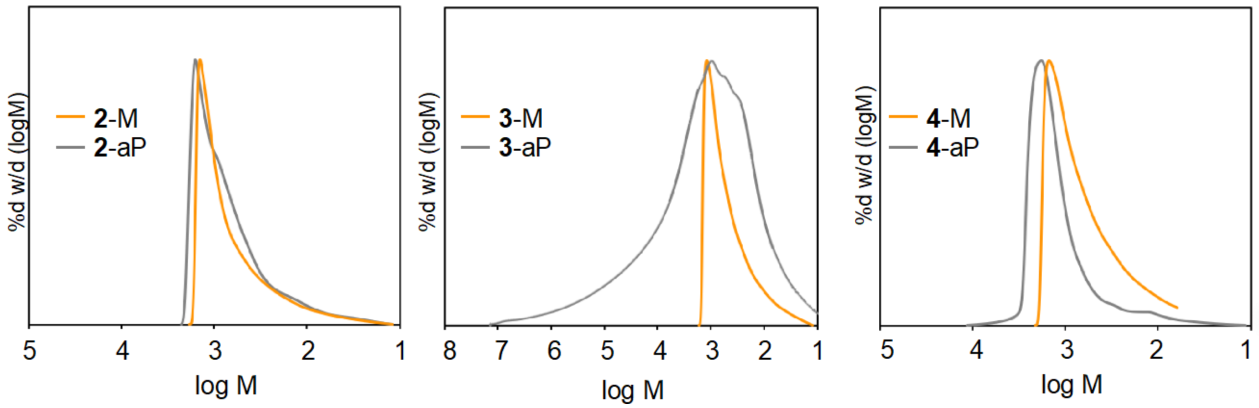

Figure S47. Differential molecular weight curves of **2**–**4**-M and **2**–**4**-aP (irradiated for 72 hours), which corresponds to data in Table S4.

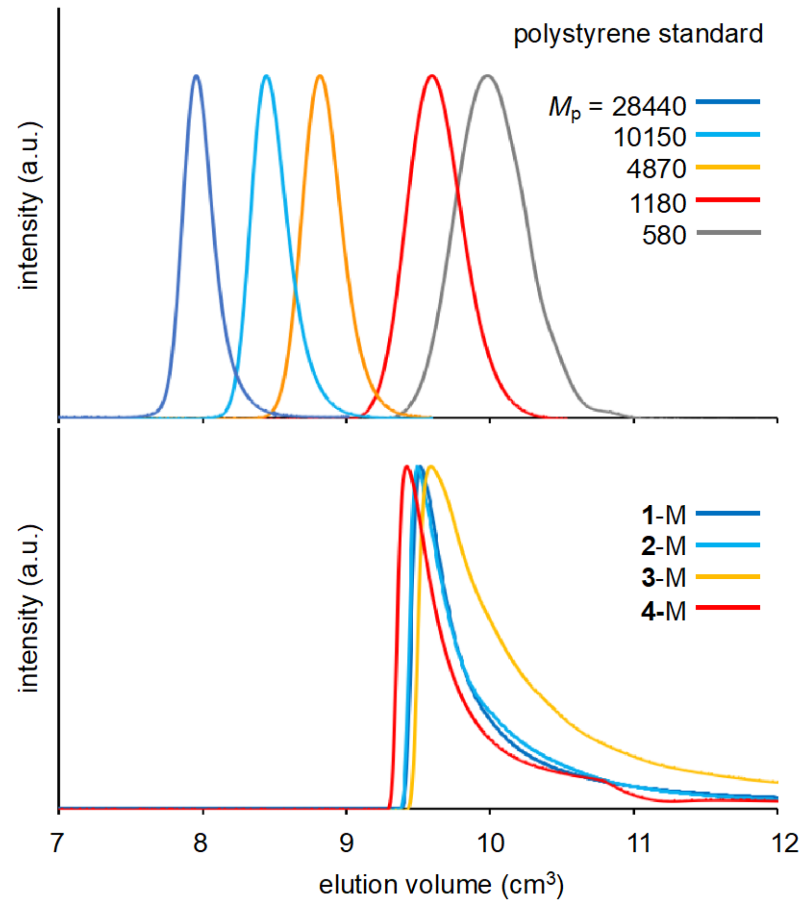

Figure S48. SEC plots of (top) polystyrene standards and (bottom) **1**–**4**-M obtained with CH_2Cl_2 elution (1 cm^3 min^{-1}). The M_n calculated from the SEC of the **1**-aP is 486 g mol^{-1}, smaller than the molecular weight of **1**-M (686 g mol^{-1}), which clearly shows the underestimation of polymer weights determined by SEC. For both monomers and polymers, the collected SEC plots spread toward the lower molecular weight region, presumably due to the Coulombic interactions between the samples and the stationary phase. The use of other solvents (*e.g.*, chloroform and acetonitrile) and the addition of LiBr were tried but did not alleviate the broadening or underestimation of molecular weights.

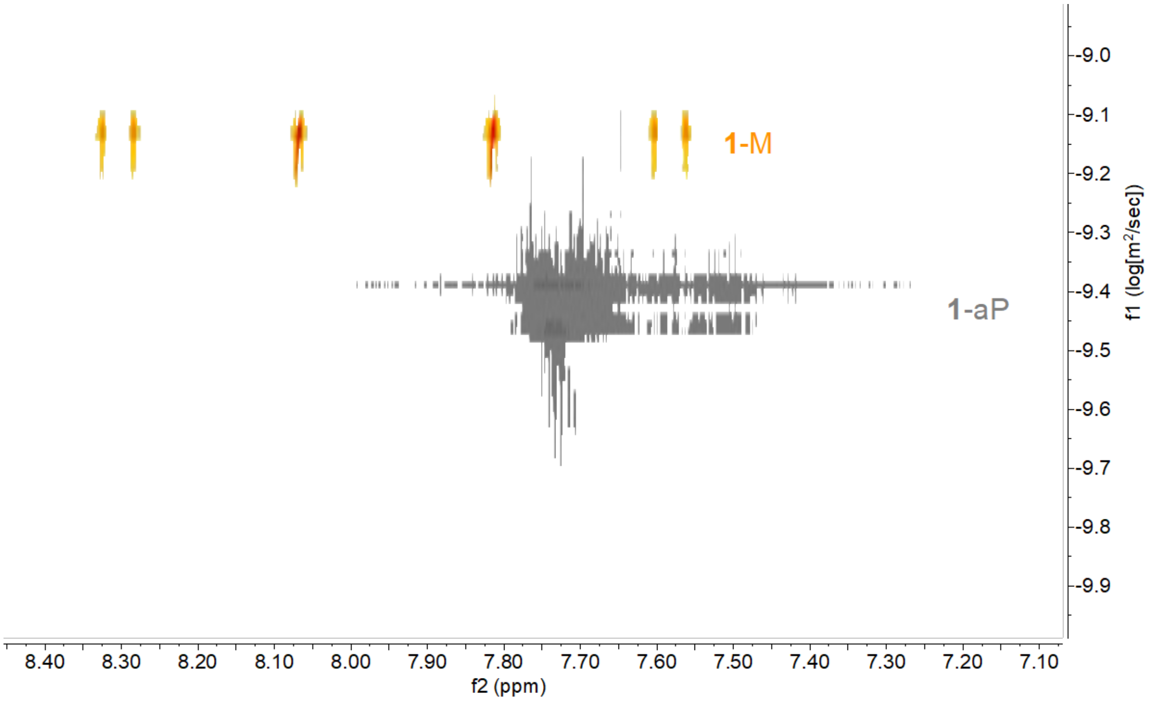

Figure S49. ^{1}H DOSY NMR spectra of **1**-M (orange) and **1**-aP (gray) measured in CD_2Cl_2 at 20 °C (400 MHz)

Table S5. The solubility of **1**-aP in selected solvents.

solvent	solubility (g/L)
CH_3CN	67.0
CH_2Cl_2	6.5
acetic acid	6.1
$CHCl_3$	2.3

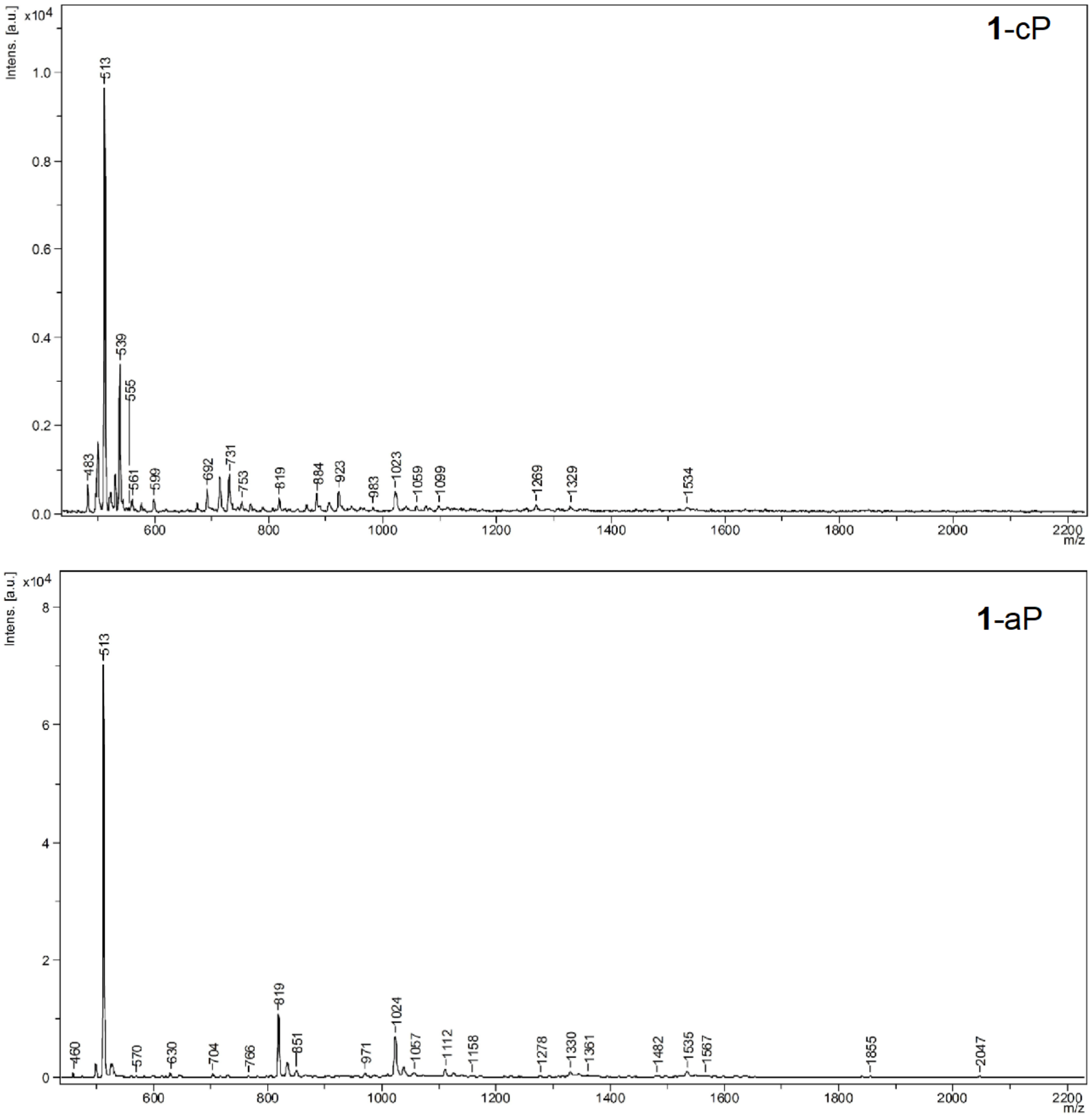

Figure S50. MALDI-TOF mass (+) spectra of **1-**cP (top) and **1-**aP (bottom)

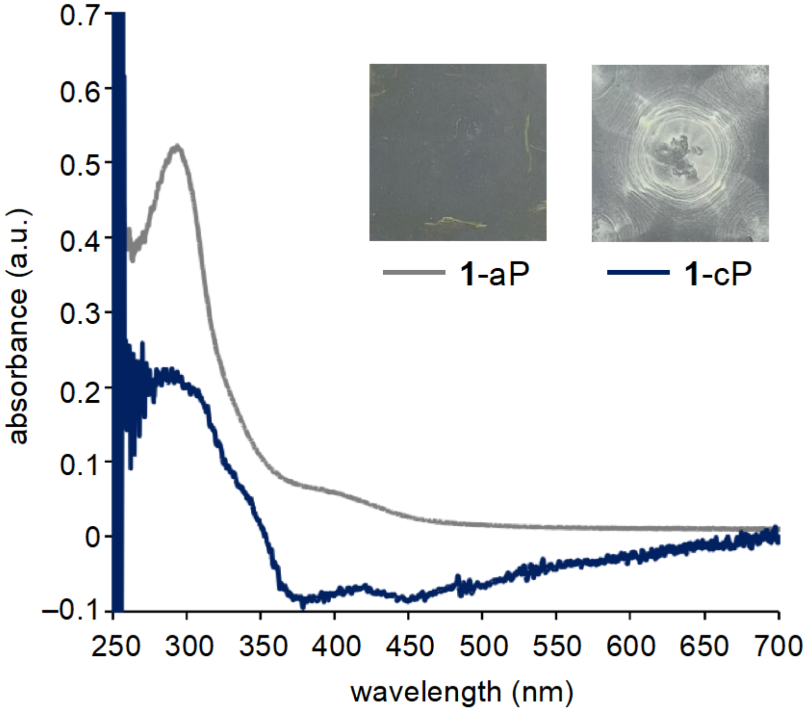

Figure S51. UV-vis absorption spectra of **1**-aP (gray) and **1**-cP (blue) and the photographs of spin-coated films.

15. Thermal analyses

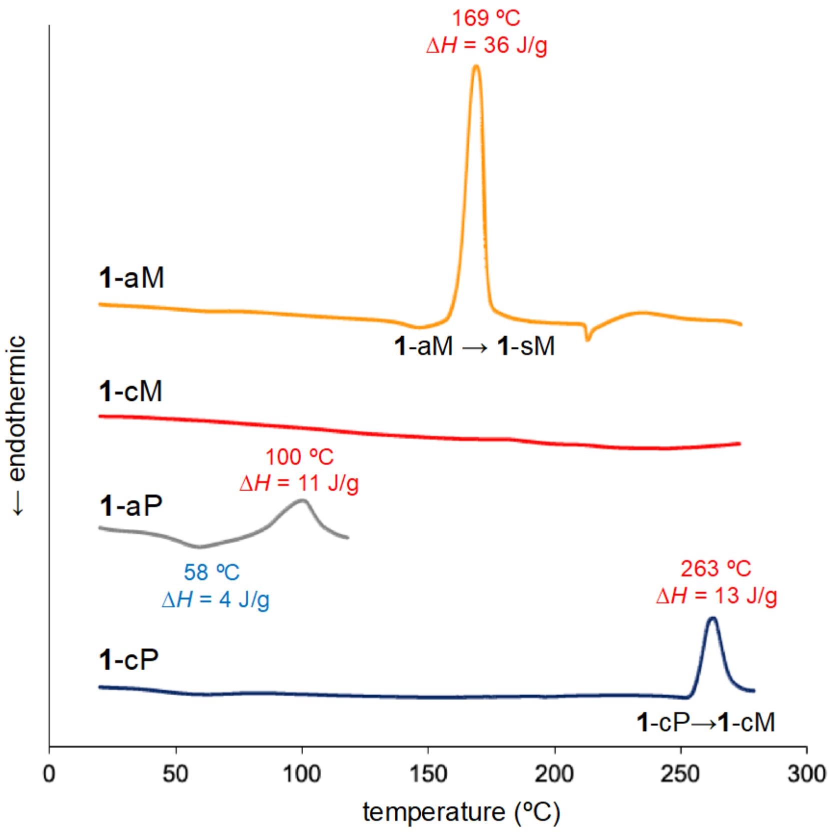

Figure S52. DSC plots of **1**-aM (orange), **1**-cM (red), **1**-aP (gray), and **1**-cP (blue) measured at 10 °C/min.

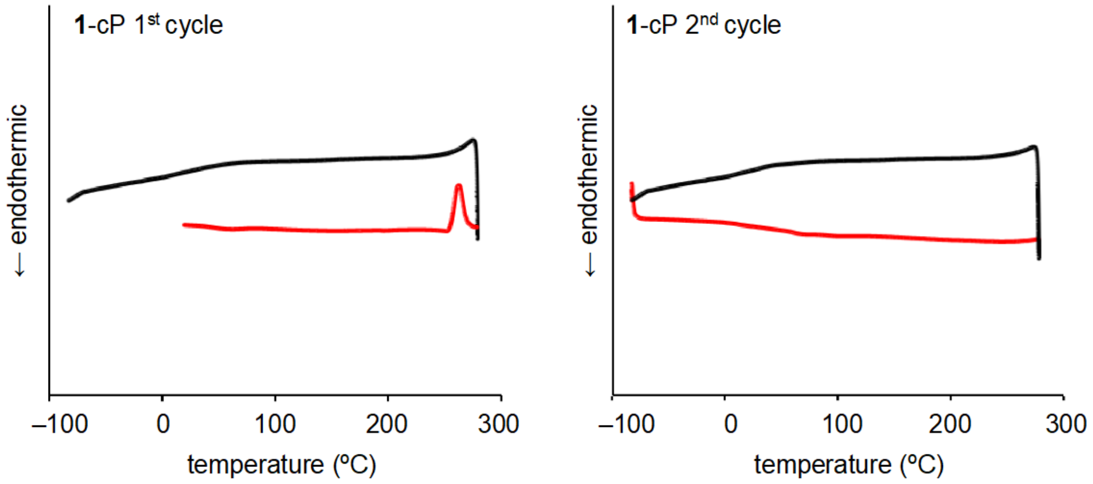

Figure S53. The 1st and 2nd cycles of DSC of **1**-cP measured at 10 °C/min. Heating(red), cooling (black).

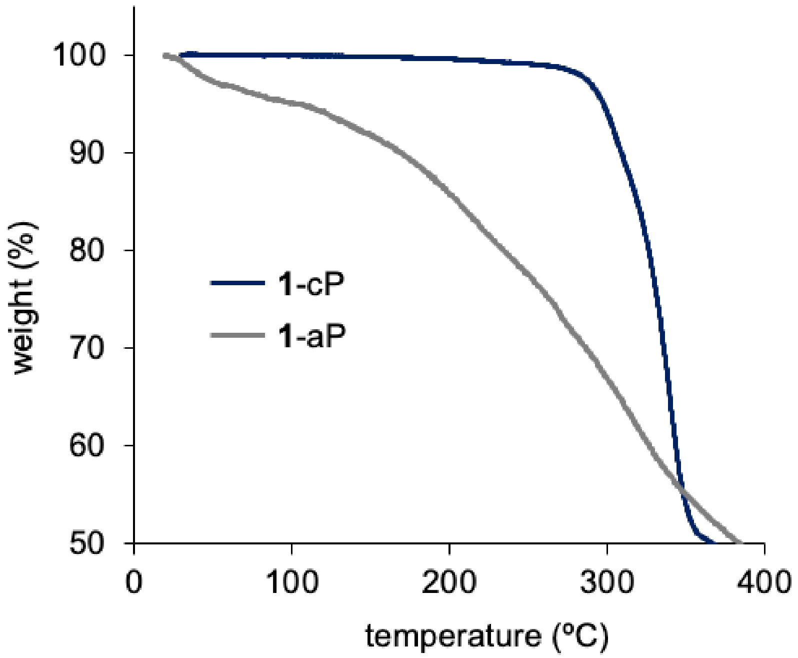

Figure S54. TGA plots of **1**-cP (blue) and **1**-aP (gray) measured at 20 °C/min.

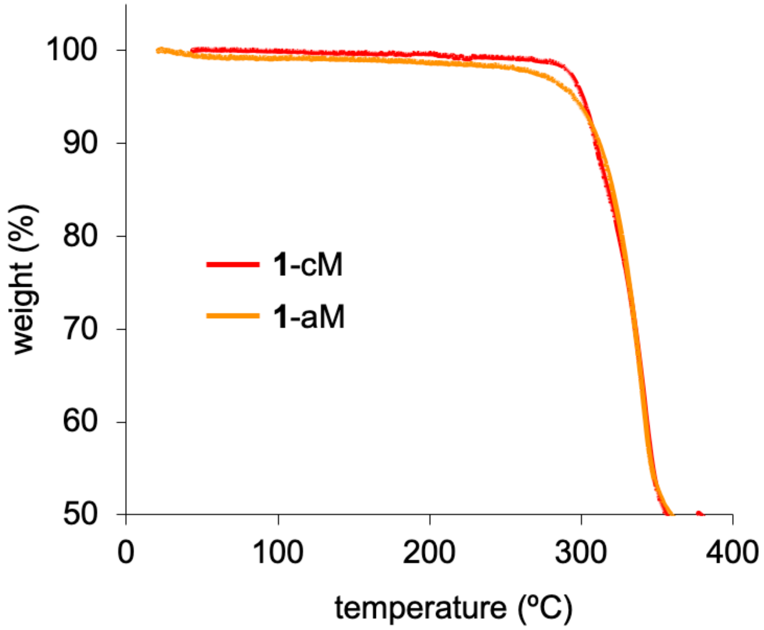

Figure S55. TGA plots of **1**-cM (red) and **1**-aM(orange) measured at 20 °C/min.

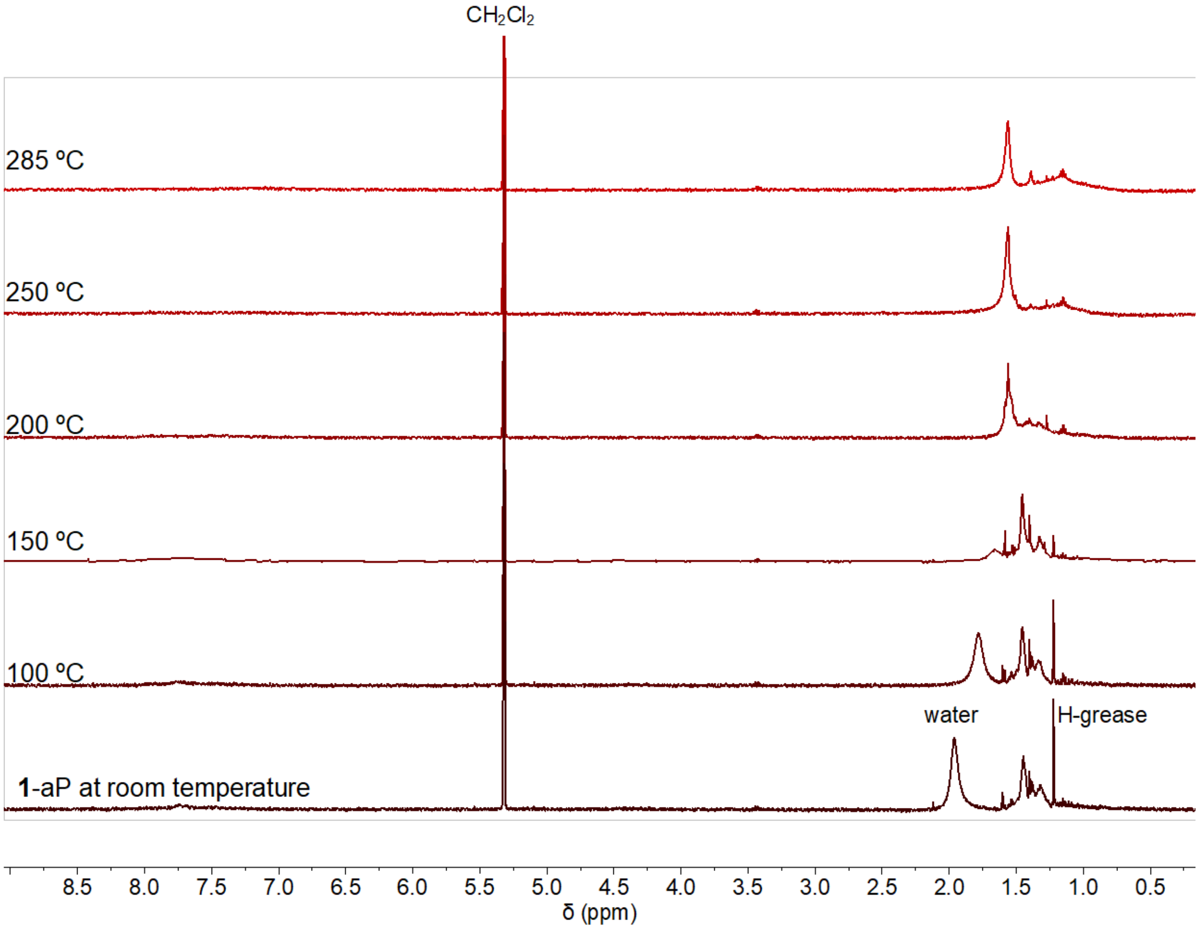

Figure S56. NMR spectra of **1**-aP measured after heating in solid under nitrogen atmosphere.

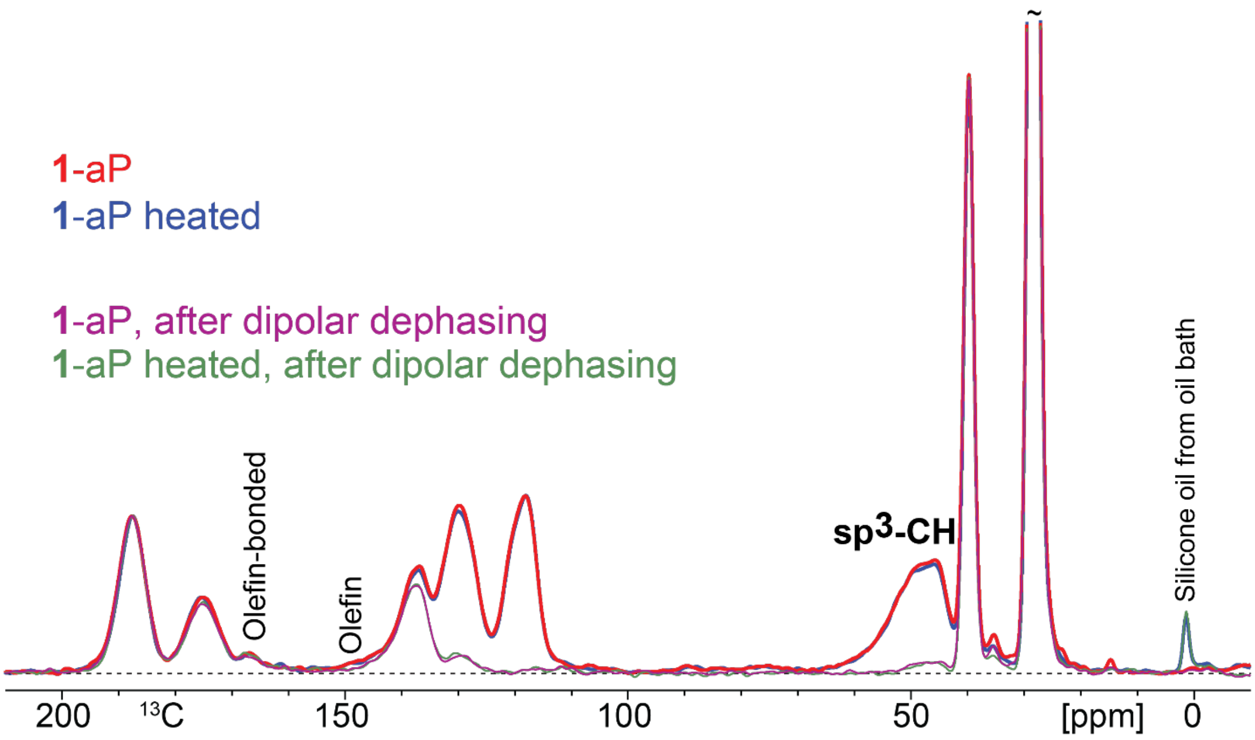

Figure S57. Solid-state ^{13}CNMR of **1**-aP, before heating (red) and after heating at 120 ºC for 2 min in an oil bath (blue), Thin lines: spectra after dipolar dephasing, which suppresses the signals of immobile CH groups (purple and green).

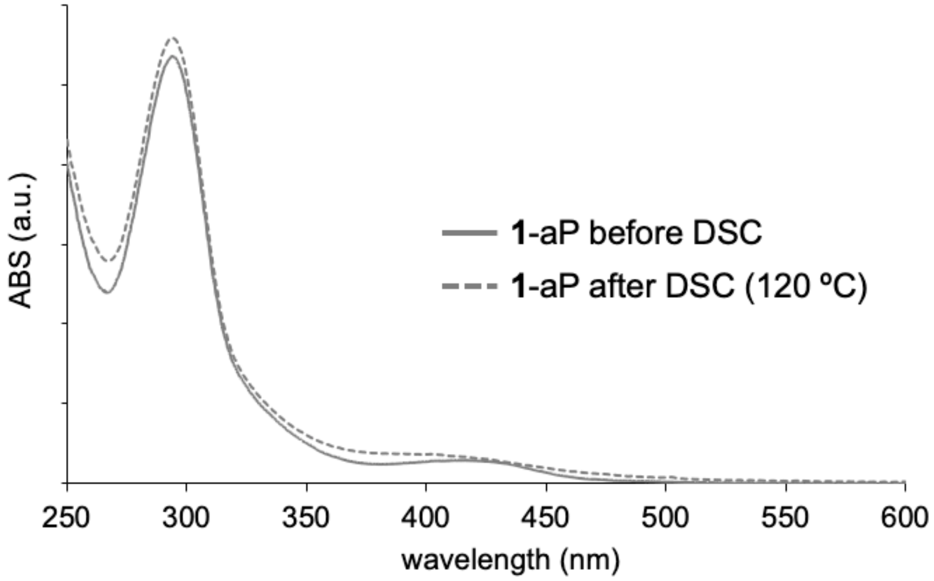

Figure S58. UV-vis absorption spectra of **1**-aP before (solid line) and after heating in DSC (dashed line) in CH_2Cl_2 (29 μM)

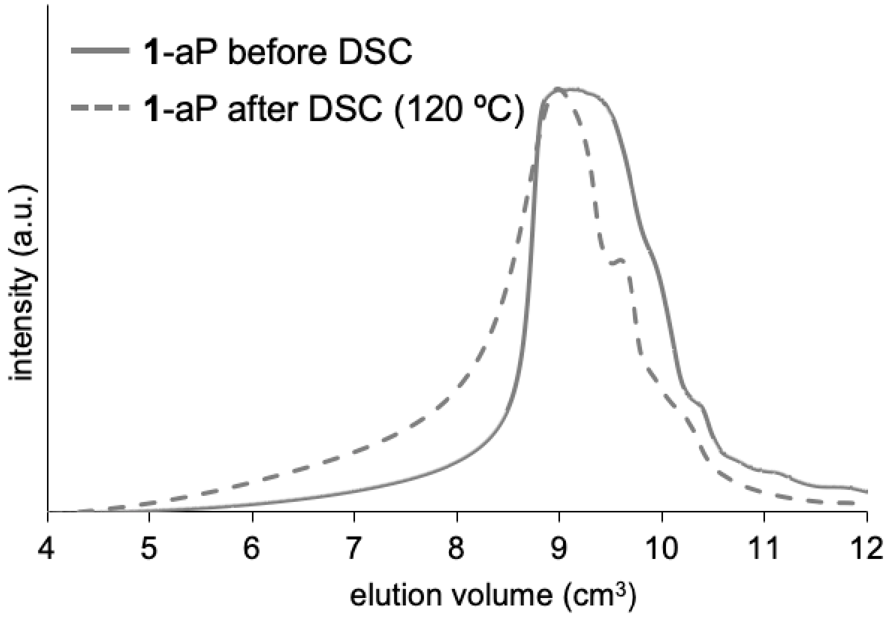

Figure S59. SEC plots of **1**-aP before (solid line) and after heating in DSC (dashed line), obtained by CH_2Cl_2 elution (1 mL/cm^3).

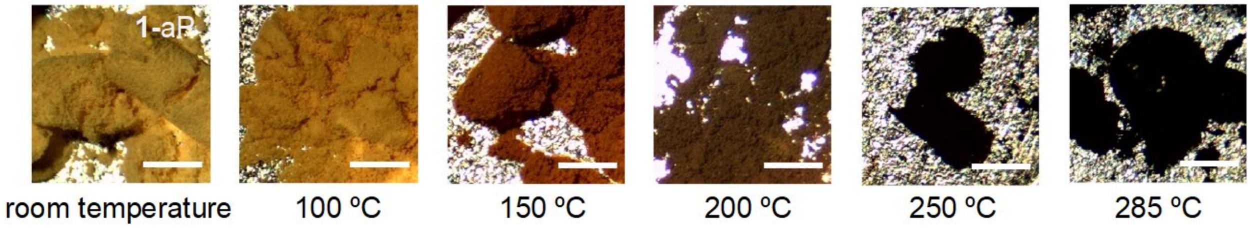

Figure S60. Optical microscope images of heated **1**-aP in solid under nitrogen atmosphere. Scale bar = 1 mm

16. DFT calculation of isomers

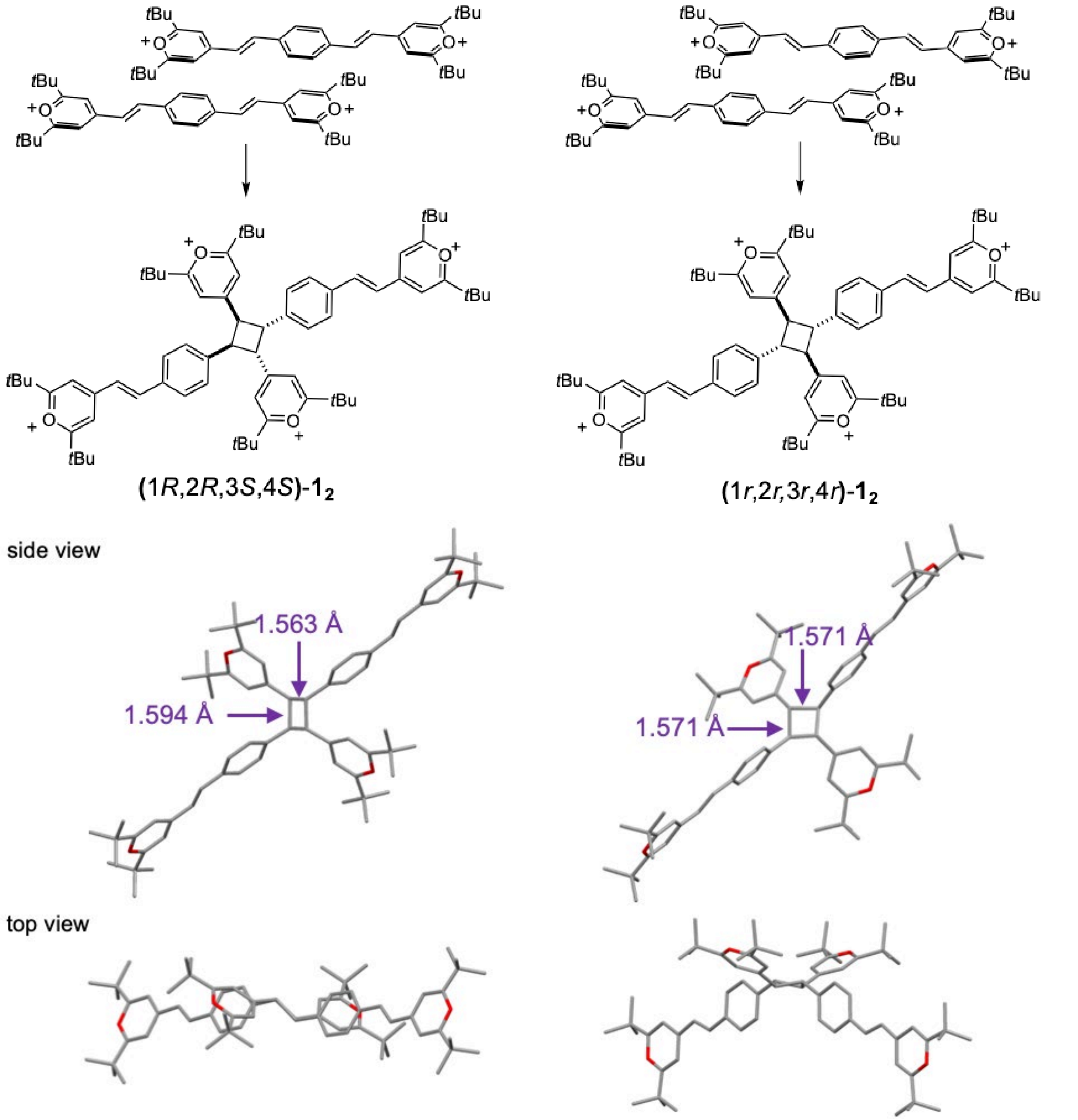

Figure S61. Optimized structures of (1*R*,2*R*,3*S*,4*S*)-**1**$_2$ and (1*r*,2*r*,3*r*,4*r*)-**1**$_2$ calculated at the B3LYP/6-31+G** level of theory. Hydrogen atoms are omitted for clearly.

Table S6. Cartesian coordinates (Å) of the optimized geometry for (1*R*,2*R*,3*S*,4*S*)-**1**$_2$

C	-5.231170	-0.034670	-0.048970
C	-2.980760	0.284580	-0.949780
H	-2.360920	0.557030	-1.800490
C	-4.364170	0.317220	-1.104760
H	-4.781830	0.609670	-2.063780
C	-6.675130	-0.008090	-0.292240
H	-6.950070	0.299060	-1.298920
C	-7.659170	-0.327020	0.586170
H	-7.402710	-0.634500	1.595860
O	1.135790	-4.982940	-0.405460
C	-2.400480	-0.106930	0.268150
C	-4.647200	-0.405440	1.179150
H	-5.267350	-0.681440	2.025100
C	0.402750	-2.318520	-0.515090
C	-3.264200	-0.439500	1.329150
H	-2.850710	-0.741490	2.288420
C	1.474240	-2.821590	-1.286400
H	2.043830	-2.187520	-1.954620
C	1.802360	-4.151920	-1.206400
C	-0.901690	-0.220870	0.465270
H	-0.727340	-0.708640	1.428420
C	-0.052910	-0.893810	-0.662020
H	-0.612430	-0.884430	-1.606870
C	-0.272760	-3.235870	0.316320
H	-1.117640	-2.942640	0.927440
C	0.121640	-4.552410	0.340350
O	11.828510	0.136300	0.654730
C	5.242610	0.010880	0.119420
C	2.926170	-0.276610	0.846110
H	2.239450	-0.556820	1.641320
C	9.115020	0.208120	0.053750
C	4.293470	-0.350300	1.098690
H	4.633670	-0.681910	2.075410
C	10.104860	0.581780	-0.891980
H	9.836690	0.911370	-1.889360
C	11.431340	0.532150	-0.556230
C	6.664120	-0.057900	0.464510
H	6.859350	-0.409000	1.475430
C	7.715170	0.273470	-0.327570
H	7.538170	0.626890	-1.339250
C	9.588950	-0.204880	1.326980
H	8.923840	-0.508630	2.125320
C	10.933210	-0.224400	1.578530
O	-1.240570	5.062610	-0.080690
C	2.445420	0.165710	-0.397450
C	4.758090	0.431720	-1.135340
H	5.444200	0.715590	-1.925920
C	-0.418170	2.431900	0.163600
C	3.390810	0.505640	-1.383050
H	3.055540	0.846590	-2.359590
C	-1.486770	2.947010	0.932030
H	-2.017570	2.336390	1.652010
C	-1.860060	4.259230	0.783920
C	0.967480	0.329990	-0.695400
H	0.875270	0.816660	-1.669780
C	0.084470	1.033070	0.390220
H	0.629360	1.094320	1.341460
C	0.206810	3.320680	-0.735420
H	1.046750	3.021950	-1.350240
C	-0.230700	4.620890	-0.825270
C	-9.084060	-0.303350	0.304760
O	-11.834340	-0.305580	-0.100610
C	-9.656220	0.044380	-0.947360
C	-9.995600	-0.652150	1.334830
C	-11.343550	-0.640480	1.094230
C	-11.015240	0.029330	-1.101520
H	-9.056420	0.323560	-1.804270
H	-9.650010	-0.932500	2.323410
C	2.914831	-4.858834	-1.985271
C	2.307385	-5.965051	-2.852021
H	3.103771	-6.471111	-3.409594
H	1.590982	-5.525932	-3.555695
H	1.792547	-6.690019	-2.211256
C	3.915574	-5.472238	-1.002311
H	4.711961	-5.978299	-1.559883
H	3.400736	-6.197206	-0.361546
H	4.350427	-4.680329	-0.381829
C	3.634007	-3.846128	-2.880354
H	4.430394	-4.352188	-3.437927
H	4.068860	-3.054218	-2.259872
H	2.917603	-3.407010	-3.584028
C	-0.509985	-5.671065	1.173189
C	-1.664733	-5.101052	2.001201
H	-1.284661	-4.318959	2.668353
H	-2.116897	-5.901866	2.597406
H	-2.419294	-4.674274	1.330593
C	0.544059	-6.267230	2.109960
H	1.370711	-6.675287	1.517210

H	0.091895	-7.068044	2.706166
H	0.924131	-5.485138	2.777112
C	-1.040908	-6.763569	0.241245
H	-0.214257	-7.171625	-0.351505
H	-1.795469	-6.336791	-0.429362
H	-1.493071	-7.564383	0.837452
C	0.345731	5.707663	-1.736649
C	0.862740	6.868477	-0.882732
H	1.645664	6.504854	-0.207437
H	0.037277	7.285104	-0.294302
H	1.275390	7.646468	-1.535163
C	1.498818	5.125678	-2.558626
H	2.281742	4.762055	-1.883331
H	1.911468	5.903670	-3.211056
H	1.128705	4.294684	-3.169921
C	-0.747934	6.215609	-2.679968
H	-0.335283	6.993599	-3.332399
H	-1.573396	6.632236	-2.091537
H	-1.118046	5.384614	-3.291262
C	-2.975569	4.975522	1.549771
C	-2.383393	6.148659	2.335289
H	-1.636177	5.773704	3.044015
H	-3.181955	6.661431	2.883540
H	-1.908126	6.850658	1.640642
C	-3.639469	3.994900	2.520122
H	-2.892253	3.619944	3.228848
H	-4.063391	3.155085	1.957791
H	-4.438031	4.507673	3.068373
C	-4.019353	5.499296	0.559753
H	-3.544086	6.201295	-0.134894
H	-4.817915	6.012069	1.108005
H	-4.443275	4.659480	-0.002576
C	-11.776469	0.375824	-2.383874
C	-10.779429	0.748446	-3.484340
H	-10.110992	-0.099163	-3.673991
H	-10.189169	1.614759	-3.164480
H	-11.324372	0.996491	-4.402341
C	-12.710207	1.559847	-2.118951
H	-12.119948	2.426160	-1.799091
H	-13.423961	1.293097	-1.331159
H	-13.255151	1.807891	-3.036953
C	-12.601002	-0.834328	-2.830686
H	-11.932566	-1.681937	-3.020337
H	-13.145945	-0.586282	-3.748688
H	-13.314756	-1.101077	-2.042894
C	-12.441649	-0.990673	2.101952
C	-13.363252	0.216901	2.292672
H	-12.780261	1.065751	2.667894
H	-14.149351	-0.033793	3.014072
H	-13.819894	0.483213	1.332583
C	-11.803767	-1.362684	3.443099
H	-11.144018	-2.227153	3.306568
H	-12.589866	-1.613377	4.164499
H	-11.220776	-0.513834	3.818320
C	-13.256028	-2.176430	1.577806
H	-12.596279	-3.040899	1.441274
H	-13.712671	-1.910117	0.617717
H	-14.042127	-2.427123	2.299206
C	11.593383	-0.641429	2.895428
C	10.512889	-1.040412	3.904044
H	10.985488	-1.338951	4.846775
H	9.932280	-1.879777	3.504574
H	9.847404	-0.188131	4.082823
C	12.404434	0.531078	3.453447
H	13.177930	0.816700	2.731407
H	12.877033	0.232539	4.396178
H	11.738949	1.383359	3.632227
C	12.605433	0.902688	-1.466267
C	12.072885	1.344883	-2.831850
H	12.913385	1.610141	-3.483320
H	11.420070	2.216030	-2.704764
H	11.503964	0.525032	-3.285049
C	13.400158	2.047936	-0.833194
H	14.240659	2.313195	-1.484664
H	13.781394	1.731381	0.144389
H	12.747343	2.919083	-0.706108
C	13.517350	-0.314215	-1.643793
H	14.357850	-0.048957	-2.295263
H	12.948428	-1.134066	-2.096992
H	13.898586	-0.630770	-0.666210
C	12.522997	-1.831979	2.645691
H	13.296492	-1.546358	1.923651
H	11.942388	-2.671343	2.246221
H	12.995596	-2.130517	3.588422

Table S7. Cartesian coordinates (Å) of the optimized geometry for (1*r*,2*r*,3*r*,4*r*)-**1**$_2$

C	-0.000010	-1.098800	1.074880
H	-0.000010	-0.024870	1.306890
C	1.107840	-1.391920	0.000140
H	1.308650	-2.469880	0.000230
C	-1.107840	-1.391920	0.000110
H	-1.308650	-2.469880	0.000200
C	0.000020	-1.098980	-1.074680
H	0.000010	-0.025090	-1.306880
C	0.000030	-1.834640	-2.386560
O	0.000060	-3.126180	-4.828310
C	-1.204120	-2.178820	-3.036450
C	1.204200	-2.179030	-3.036310
C	1.160050	-2.820250	-4.250810
C	-1.159950	-2.820050	-4.250940
H	-2.171000	-1.942840	-2.607470
H	2.171070	-1.943210	-2.607230
C	-2.422610	-0.641870	0.000030
C	-4.929660	0.708340	-0.000120
C	-3.636670	-1.355330	0.000040
C	-2.485760	0.760860	-0.000060
C	-3.711310	1.420060	-0.000130
C	-4.864420	-0.699470	-0.000030
H	-3.624800	-2.442850	0.000110
H	-1.577860	1.358480	-0.000070
H	-3.727590	2.505940	-0.000200
H	-5.771170	-1.294640	-0.000030
C	-0.000030	-1.834230	2.386890
O	-0.000070	-3.125340	4.828860
C	1.204120	-2.178360	3.036810
C	-1.204200	-2.178430	3.036730
C	-1.160060	-2.819440	4.251340
C	1.159930	-2.819380	4.251410
H	2.171000	-1.942510	2.607770
H	-2.171070	-1.942640	2.607630
C	2.422610	-0.641870	0.000100
C	4.929660	0.708340	0.000070
C	3.636670	-1.355330	0.000300
C	2.485760	0.760860	-0.000110
C	3.711310	1.420060	-0.000110
C	4.864420	-0.699470	0.000290
H	3.624800	-2.442850	0.000470
H	1.577870	1.358480	-0.000260
H	3.727600	2.505940	-0.000270
H	5.771180	-1.294640	0.000490
C	6.180910	1.468580	0.000050
H	6.043720	2.547620	0.000180
C	7.443210	0.969890	-0.000170
H	7.599380	-0.104990	-0.000440
C	8.662420	1.758520	-0.000190
O	11.071510	3.146920	-0.000320
C	8.708530	3.177830	0.000490
C	9.915080	1.091500	-0.000910
C	11.079010	1.812410	-0.000950
C	9.916590	3.818880	0.000400
H	7.815180	3.789290	0.001140
H	9.978080	0.009240	-0.001440
C	-6.180910	1.468580	-0.000190
H	-6.043720	2.547620	-0.000330
C	-7.443210	0.969890	-0.000100
H	-7.599370	-0.104990	0.000070
C	-8.662420	1.758510	-0.000190
O	-11.071510	3.146920	-0.000310
C	-8.708530	3.177830	-0.000490
C	-9.915080	1.091500	0.000050
C	-11.079010	1.812400	-0.000020
C	-9.916590	3.818880	-0.000540
H	-7.815180	3.789290	-0.000700
H	-9.978070	0.009240	0.000280
C	-2.363530	-3.258483	-5.089470
C	2.363651	-3.258878	-5.089208
C	-2.321030	-4.775846	-5.288879
H	-1.395189	-5.050416	-5.807189
H	-3.182639	-5.089708	-5.889158
H	-2.354837	-5.273652	-4.313039
C	-3.656836	-2.874938	-4.365443
H	-3.687261	-1.788701	-4.222691
H	-3.690642	-3.372745	-3.389604
H	-4.518445	-3.188801	-4.965722
C	-2.316305	-2.563098	-6.452617
H	-1.390464	-2.837667	-6.970928
H	-2.346731	-1.476861	-6.309866
H	-3.177915	-2.876961	-7.052897
C	3.656940	-2.875555	-4.365033
H	4.518564	-3.189557	-4.965219
H	3.690554	-3.373375	-3.389194
H	3.687530	-1.789324	-4.222270
C	2.320919	-4.776233	-5.288633
H	3.182543	-5.090235	-5.888819

H	1.395090	-5.050642	-5.807049
H	2.354533	-5.274052	-4.312794
C	2.316695	-2.563475	-6.452355
H	3.178319	-2.877476	-7.052541
H	2.347285	-1.477243	-6.309592
H	1.390866	-2.837885	-6.970771
C	2.363510	-3.257723	5.089987
C	3.656817	-2.874378	4.365855
H	4.518426	-3.188176	4.966169
H	3.687294	-1.788168	4.222905
H	3.690571	-3.372365	3.390106
C	2.316358	-2.562088	6.453008
H	3.177967	-2.875885	7.053323
H	1.390517	-2.836513	6.971394
H	2.346836	-1.475877	6.310058
C	2.320936	-4.775048	5.289673
H	3.182545	-5.088846	5.889987
H	2.354690	-5.273034	4.313924
H	1.395094	-5.049473	5.808059
C	-2.363663	-3.257871	5.089838
C	-2.321006	-4.775196	5.289503
H	-1.395179	-5.049574	5.807940
H	-2.354671	-5.273169	4.313744
H	-3.182633	-5.089057	5.889760
C	-3.656949	-2.874595	4.365634
H	-3.687487	-1.788385	4.222699
H	-4.518575	-3.188456	4.965891
H	-3.690614	-3.372568	3.389875
C	-2.316637	-2.562254	6.452874
H	-1.390810	-2.836631	6.971311
H	-3.178263	-2.876115	7.053130
H	-2.347174	-1.476044	6.309939
C	-10.127202	5.335324	-0.000851
C	-12.496662	1.234278	0.000206
C	-8.767689	6.039373	-0.001103
H	-8.205617	5.748781	0.893798
H	-8.918460	7.124953	-0.001326
H	-8.205750	5.748395	-0.895962
C	-10.912172	5.741791	1.249176
H	-10.350102	5.451199	2.144078
H	-11.885410	5.237782	1.249356
H	-11.062944	6.827371	1.248953
C	-10.912357	5.741253	-1.250936
H	-10.350419	5.450275	-2.145796
H	-11.063128	6.826833	-1.251159
H	-11.885595	5.237245	-1.250755
C	-13.241653	1.710130	1.250212
H	-12.708208	1.369870	2.145129
H	-14.256510	1.296270	1.250373
H	-13.293558	2.804900	1.249978
C	-13.241830	1.709588	-1.249900
H	-12.708512	1.368939	-2.144745
H	-13.293736	2.804358	-1.250134
H	-14.256687	1.295727	-1.249738
C	-12.424155	-0.295004	0.000533
H	-11.890710	-0.635265	0.895449
H	-11.890837	-0.635652	-0.894312
H	-13.439012	-0.708864	0.000694
C	10.127200	5.335325	0.001092
C	10.911848	5.742152	-1.249020
H	11.885086	5.238145	-1.249596
H	11.062617	6.827732	-1.248524
H	10.349547	5.451814	-2.143860
C	8.767685	6.039370	0.001896
H	8.918454	7.124951	0.002391
H	8.205978	5.748134	0.896816
H	8.205385	5.749033	-0.892944
C	10.912676	5.740896	1.251092
H	11.885914	5.236890	1.250518
H	10.350968	5.449660	2.146012
H	11.063445	6.826477	1.251587
C	12.496664	1.234293	-0.001699
C	13.241321	1.710464	-1.251782
H	14.256179	1.296606	-1.252319
H	13.293223	2.805234	-1.251284
H	12.707638	1.370429	-2.146642
C	12.424161	-0.294989	-0.002394
H	13.439019	-0.708847	-0.002930
H	11.890478	-0.635024	-0.897254
H	11.891081	-0.635866	0.892506
C	13.242163	1.709288	1.248330
H	14.257021	1.295430	1.247794
H	12.709083	1.368411	2.143230
H	13.294065	2.804058	1.248827

17. Vapor annealing and preparation of solid pellets

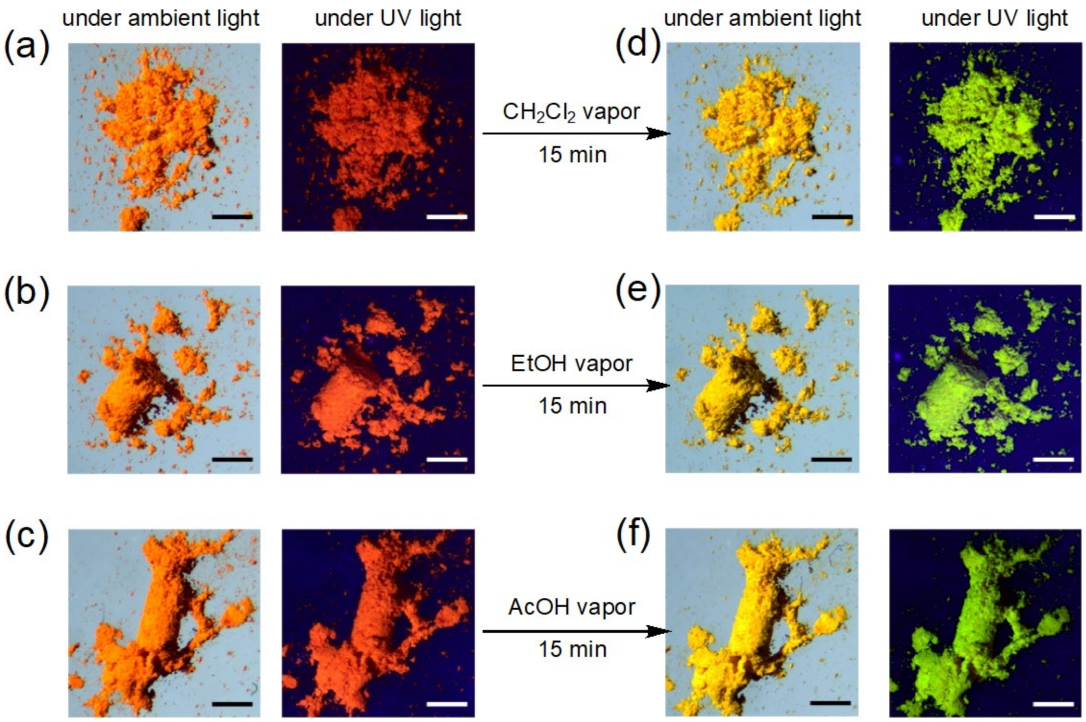

Figure S62. Optical microscope images of **1**-aM under ambient light (left) and UV light in dark (right), (a-c) before exposure to any vapor and after 15 min exposure to (d) CH_2Cl_2, (e) EtOH, and (f) AcOH vapor. Scale bar = 1 mm.

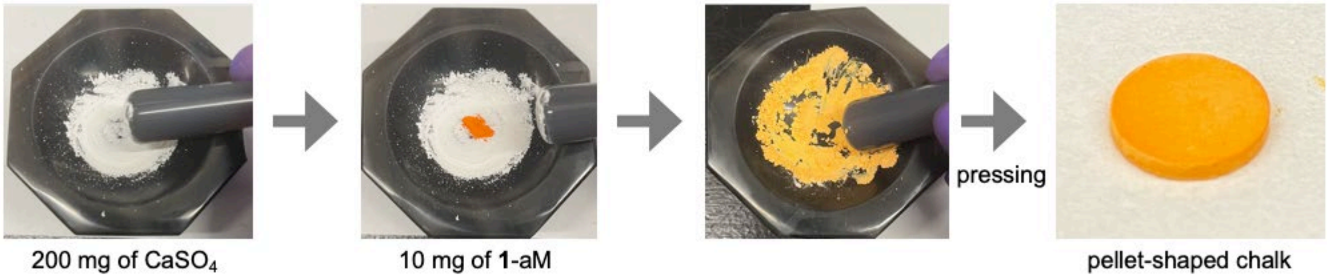

Figure S63. **1**-aP pellet made by grinding **1**-aM and $CaSO_4$, followed by pressing them in a die.

18. ^{1}H and ^{13}C NMR spectra

^{1}H NMR spectrum of **1**-M (400 MHz, CD_2Cl_2)

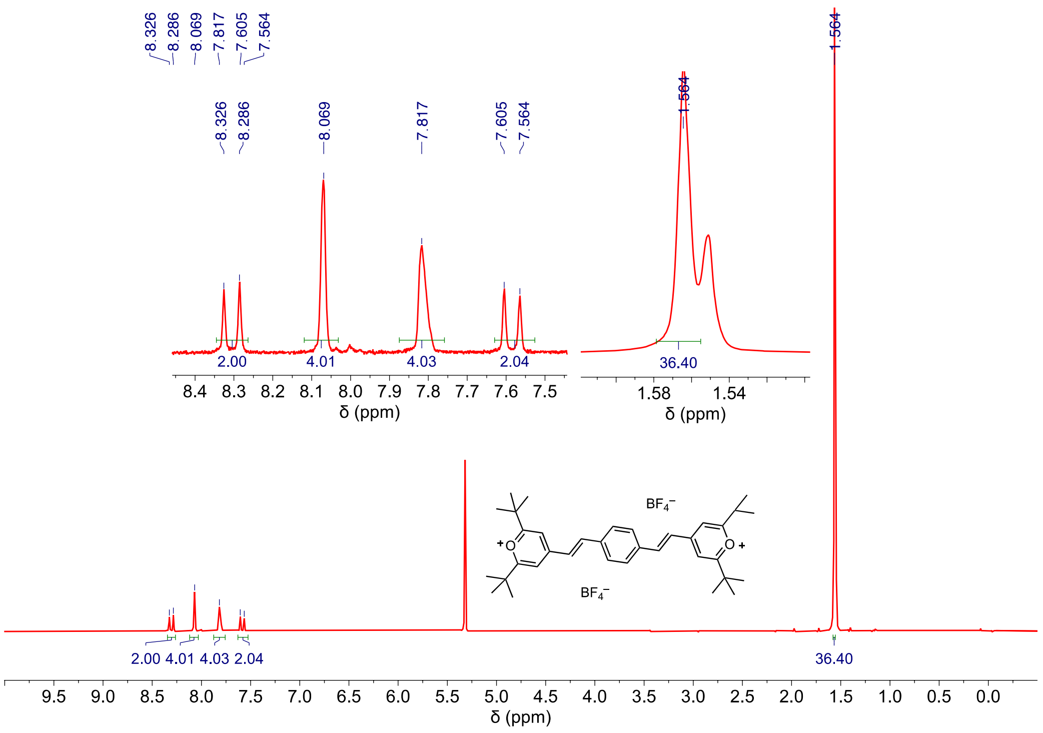

$^{13}C\{^{1}H\}$ NMR spectrum of **1**-M (201 MHz, CD_2Cl_2)

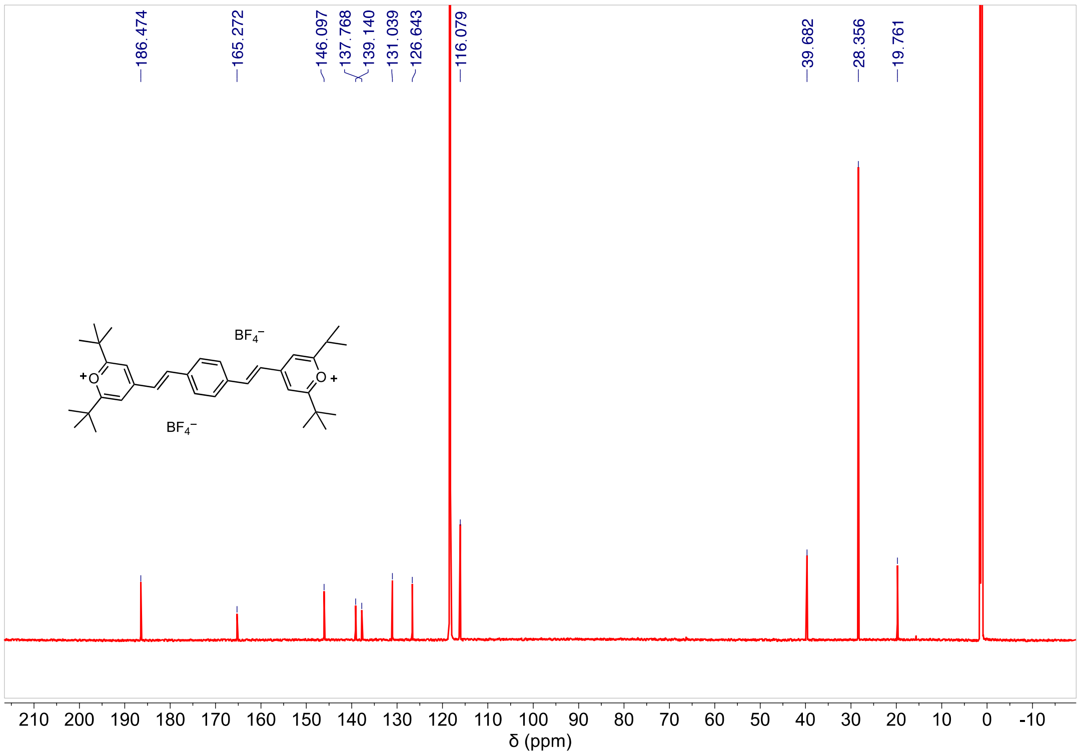

^{1}H NMR spectrum of **2**-M (400 MHz, CD_3CN)

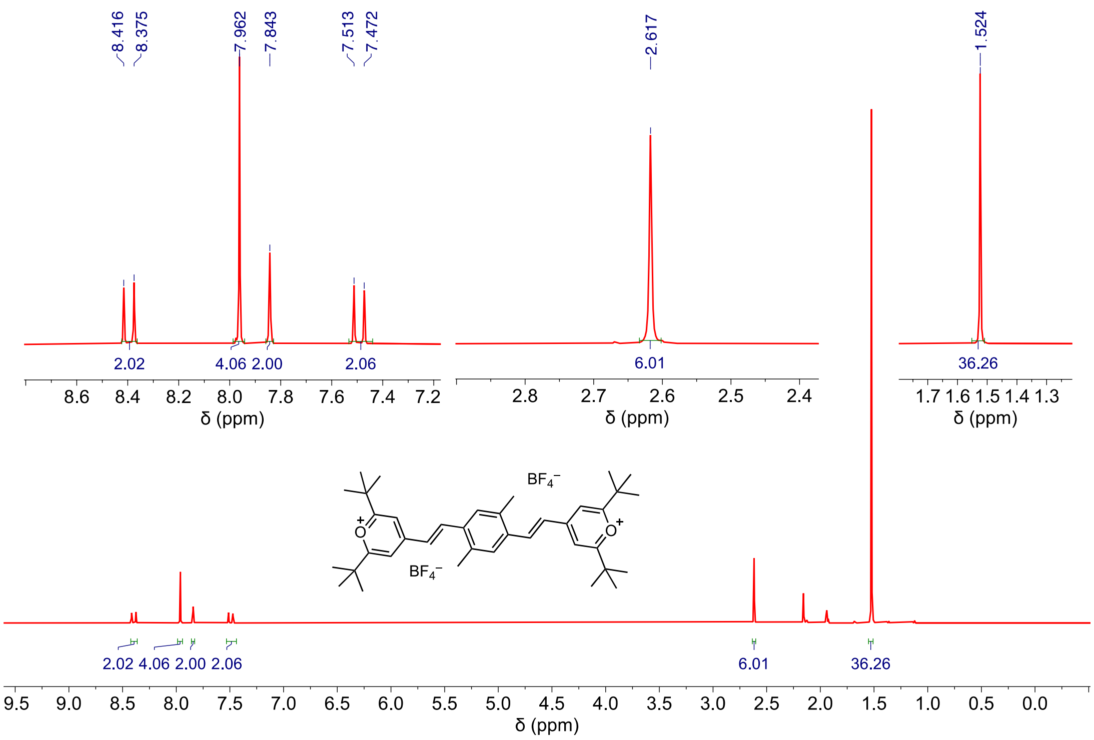

$^{13}C\{^{1}H\}$ NMR spectrum of **2**-M (201 MHz, CD_3CN)

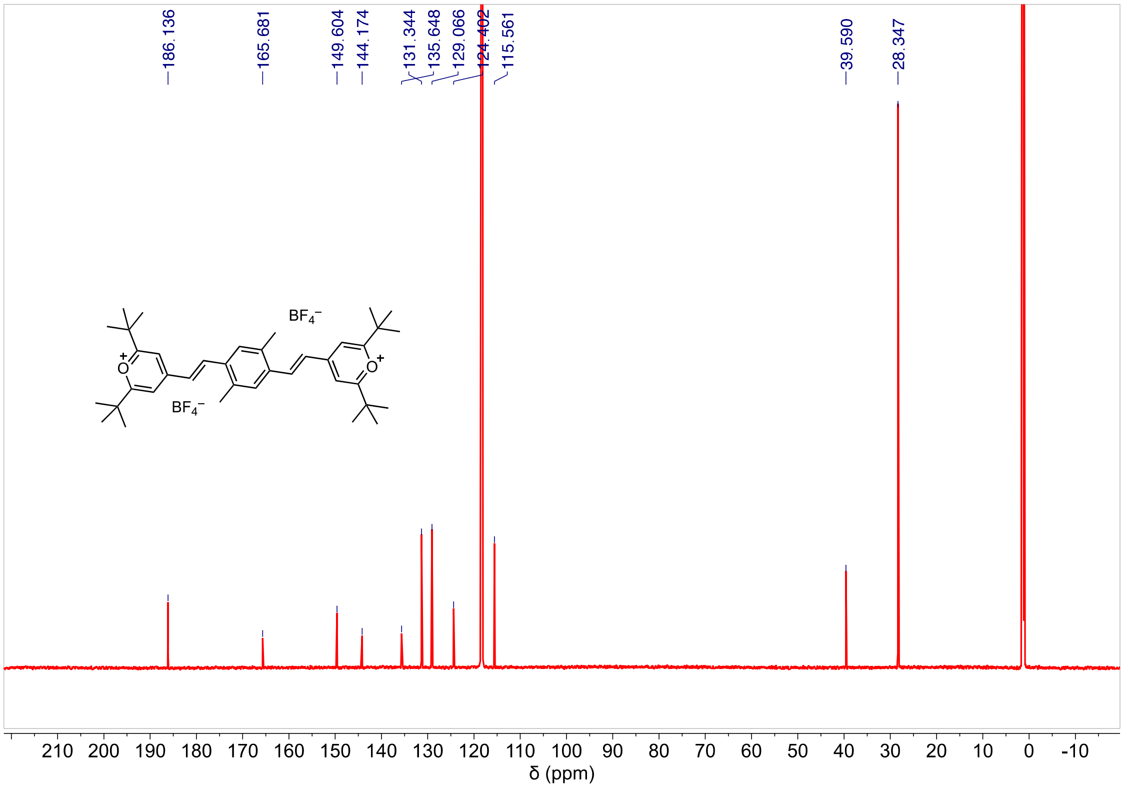

^{1}H NMR spectrum of **3**-M (400 MHz, CD_3CN)

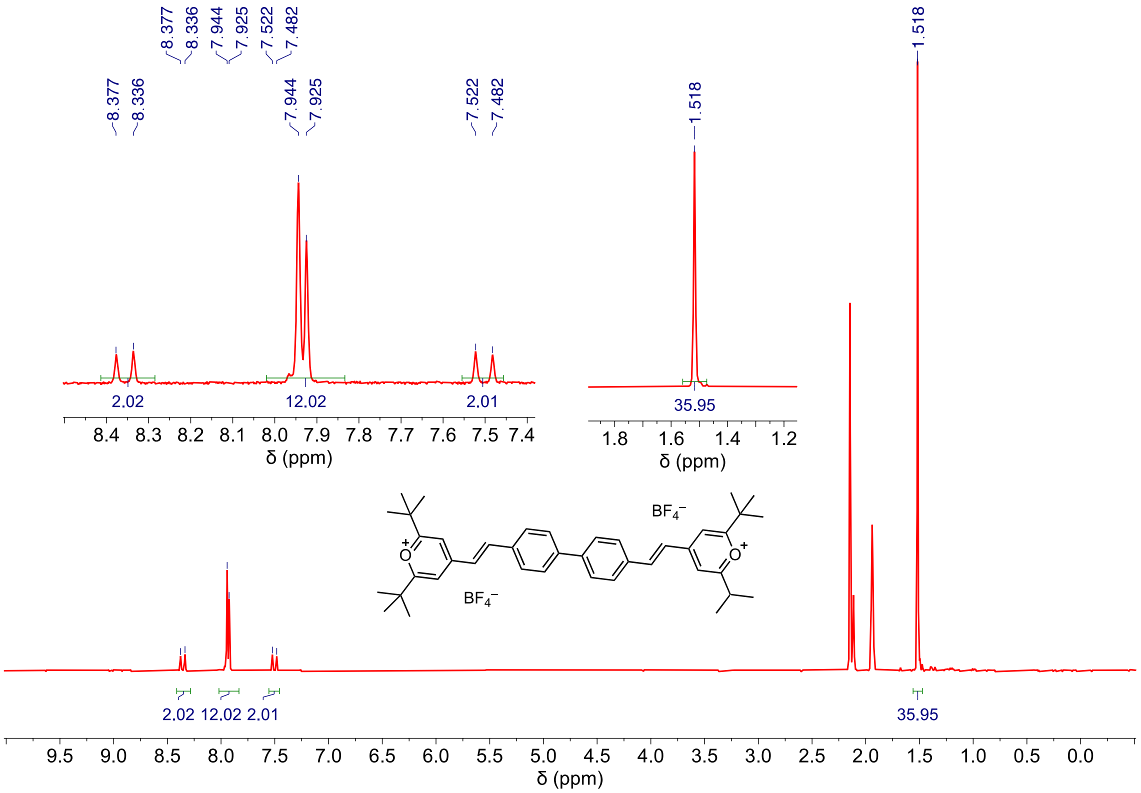

^{13}C{^{1}H} NMR spectrum of **3**-M (201 MHz, CD_3CN)

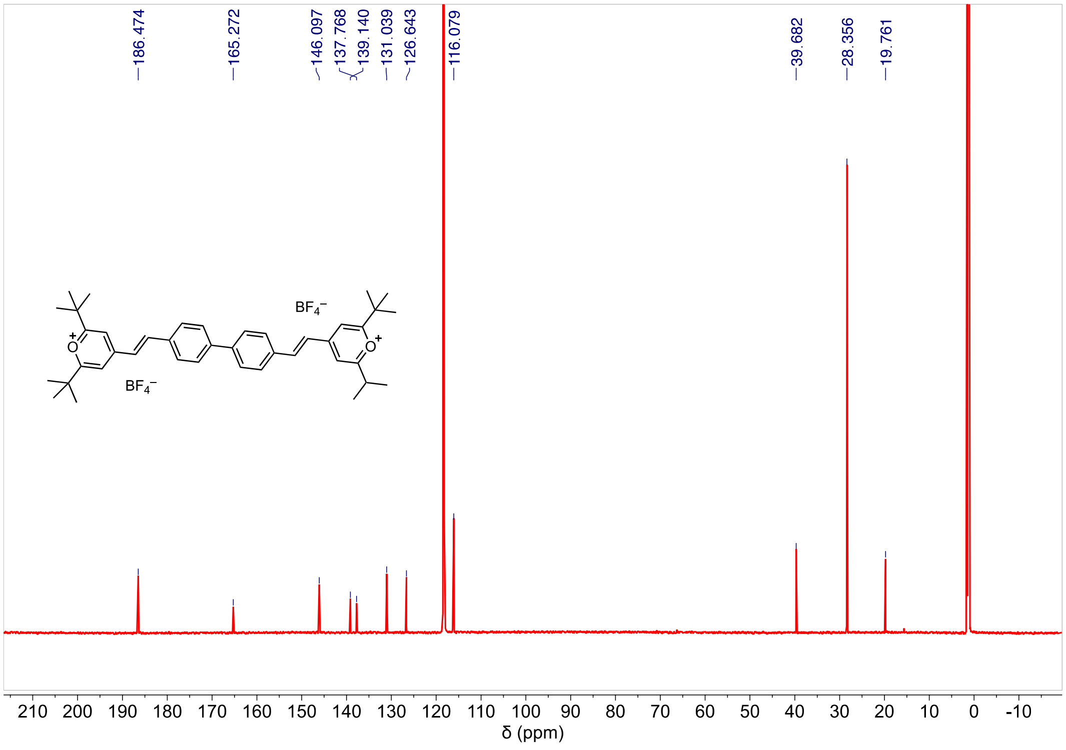

^{1}H NMR spectrum of **4**-M (400 MHz, CD_3CN)

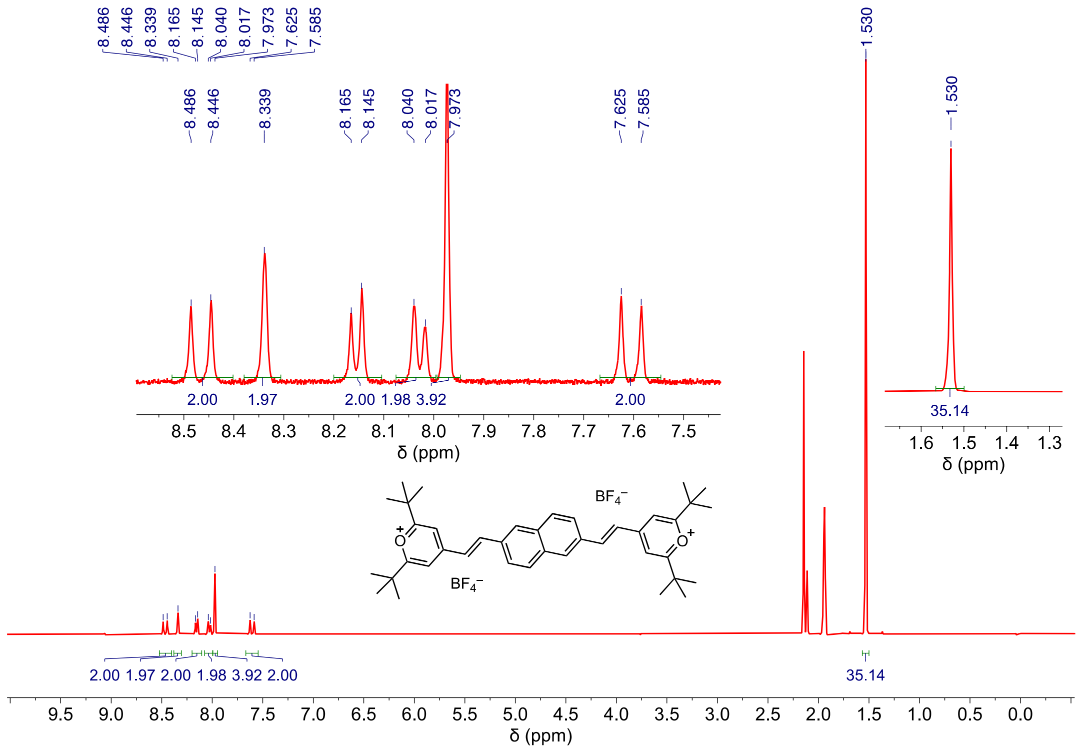

^{13}C{^{1}H} NMR spectrum of **4**-M (101 MHz, CD_3CN)

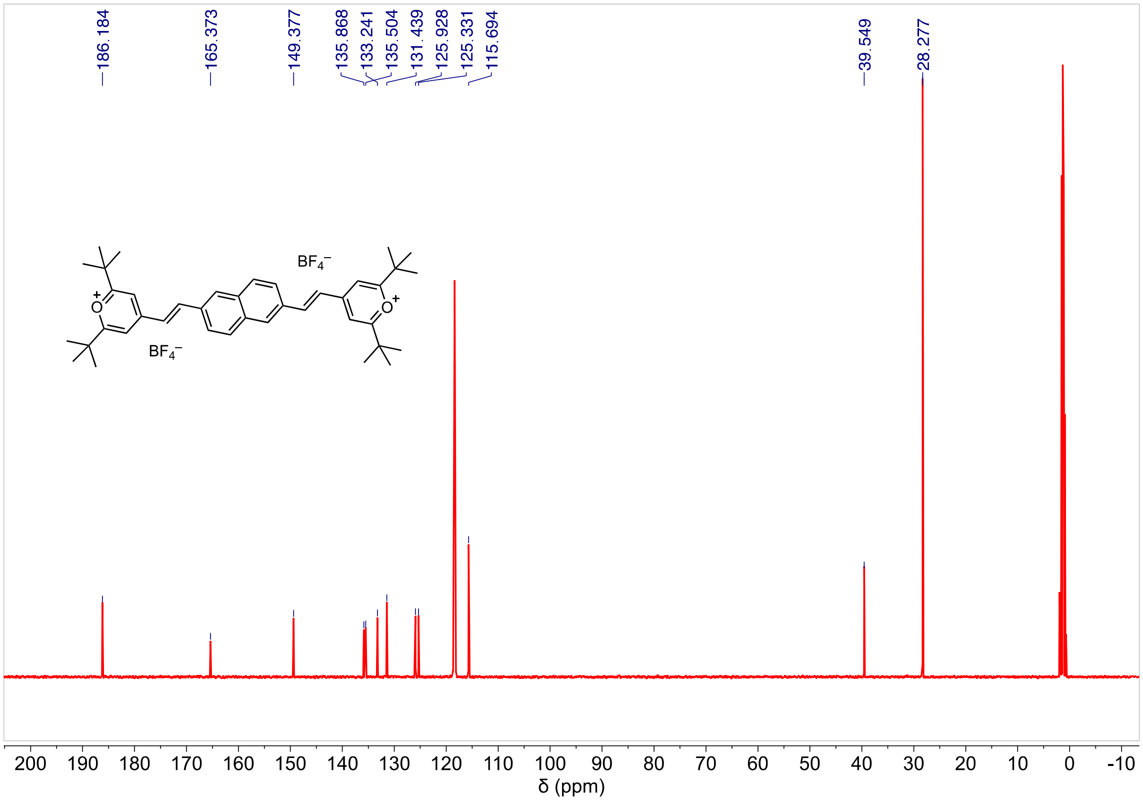

19. Supplementary References

[1] Li, Y., Wang, H. & Li, X. Over one century after discovery: pyrylium salt chemistry emerging as a powerful approach for the construction of complex macrocycles and metallo-supramolecules. *Chem. Sci.* **11**, 12249–12268 (2020)

[2] Buchholz, V. & Enkelmann, V. Photochemical Single-Crystal-To-Single-Crystal Dimerizations and Polymerizations. *Mol. Cryst. Liq. Cryst. Sci. Technol. Sect. A* **356**, 315–325 (2001)

[3] Han, G. G. D., Li, H. & Grossman, J. C. Optically-controlled long-term storage and release of thermal energy in phase-change materials. *Nat. Commun.* **8**, 1446 (2017)
