# Supplementary material for: Mechanoactivated amorphization and photopolymerization of styryldipyryliums
Source: Commun Mater. 2024 Jun 8;5(1):98. doi: 10.1038/s43246-024-00539-8 (PMC11162349; doi:10.1038/s43246-024-00539-8)
Supplement: Supplementary file 2 — Description of Additional Supplementary Files [file 43246_2024_539_MOESM2_ESM.pdf]

Description Of Additional Supplementary File

File Name: Supplementary Video 1

Description: 1-cM to 1-cP transformation in a crystal under the irradiation with a broadband tungsten-halogen lamp

File Name: Supplementary Video 2

Description: 1-M initial to 1-aM transformation by the solvent-free grinding in an agate mortar
